# Application of the OBIMAP (One-Bead Interchain Multipeptide Assembly Platform) to Long Peptide Synthesis: Liraglutide as a Case Study

**DOI:** 10.1021/acsomega.6c00648

**Published:** 2026-03-25

**Authors:** Danah AlShaer, Othman Al Musaimi, Daryl R. Williams

**Affiliations:** † Department of Chemical Engineering, 4615 Imperial College London, London SW7 2AZ, U.K.; ‡ School of Pharmacy, 12186 Newcastle University, Newcastle upon Tyne NE1 7RU, U.K.; § Orthogonal Peptides, London SW7 2AZ, U.K.

## Abstract

This paper reports the application of the recently described OBIMAP (one-bead interchain multipeptide assembly platform) to the synthesis of liraglutide, a representative long peptide (>30 residues), achieving improved yield and purity compared with conventional sequential synthesis. Two synthesis strategies were tested: resin-stapled elongation (RSE) and parallel chain ligation (PCL). RSE, in which two peptide fragments are covalently “stitched” on-resin via orthogonal protection, delivered superior results, up to ∼50% crude purity and 76% recovery versus ∼25% crude purity and 37% recovery for conventional SPPS, while maintained same solvent use as in sequential SPPS. The improved overall purity suggested a reduction in aggregation behavior resulting from the midchain “stitching” step. A current limitation of the process is the extended synthesis time, approximately three times longer than conventional sequential methods, with fragment stitching (linking) representing the most time-consuming step (9–16 h at room temperature). Integration of microwave-assisted fragment linking and automation of three out of nine steps has reduced overall synthesis time to half, while automation of the remaining steps is currently under validation and investigation. The primary challenge for full automation lies in ensuring the compatibility of automated synthesizers with the remaining synthesis steps. Subsequent studies are focused on extending the results obtained at the 0.1 mmol scale to larger production scales (1, 10, and 50 mmol). If successfully implemented at larger scales this strategy, OBIMAP-RSE, could reduce manufacturing costs, enhance accessibility, and accelerate the development of next-generation peptide therapeutics.

## Graphical abstract

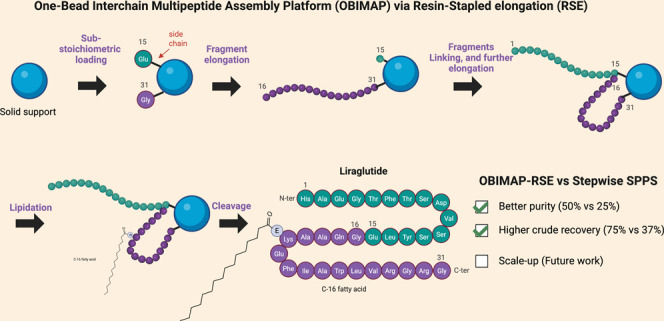

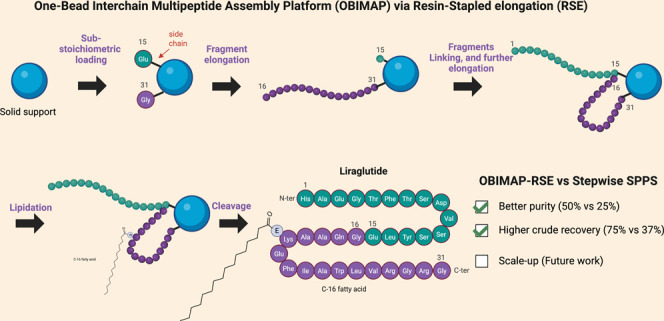

## 1 Introduction

Glucagon-like peptide-1 (GLP-1) is an insulinotropic hormone derived from the intestine that has gained significant attention for its proven ability to lower blood glucose levels. Due to its short half-life of approximately 1.5–5 min, numerous chemically modified analogues have been developed to extend its half-life and enhance its clinical utility (Figure 1).^1,2^ The development of these analogues marks a major advance in modern therapeutics. Initially introduced for type 2 diabetes, their benefits now extend to obesity and cardiovascular disease. Drugs such as semaglutide (Novo Nordisk, *Ozempic*), dulaglutide (Eli Lilly, *Trulicity*), and the dual GLP-1 GIP analogue tirzepatide (Eli Lilly, *Mounjaro*) exemplify this success, generating annual revenues exceeding $70 billion in 2025 and is projected to reach USD 201.79 billion by 2033.^3^ Their rapid adoption reflects a global shift toward GLP-1-based therapies, driven by their impact on two of the most urgent health challenges in the Western world. As a result, demand for GLP-1 analogues is expected to rise substantially, positioning peptide therapeutics to surpass monoclonal antibodies and oligonucleotides in both market value and clinical significance.

**Figure 1:**
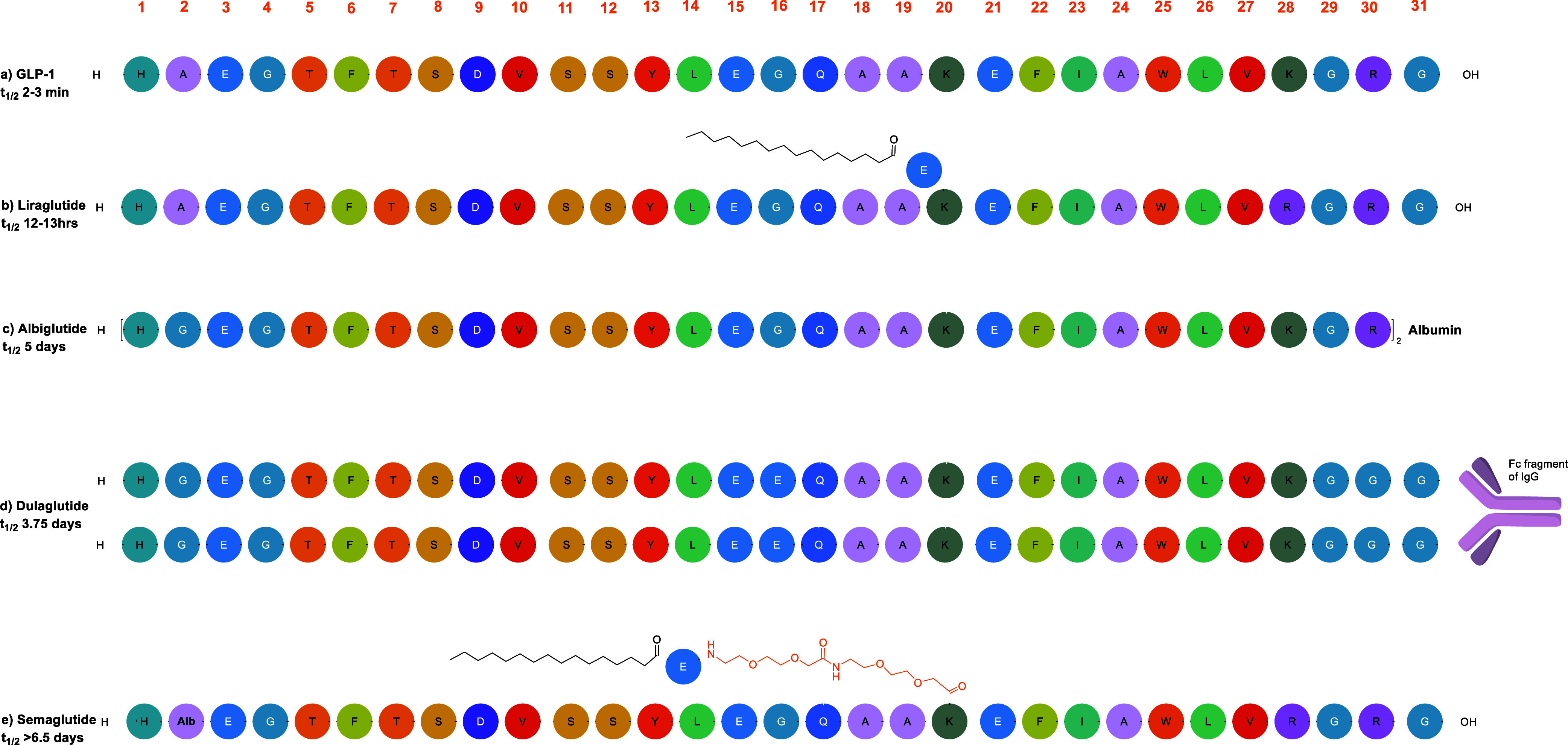
Peptide sequences of (a) GLP-1 and some of its synthetic analogues, (b) Liraglutide, (c) Albiglutide, (d) Dulaglutide, and (e) Semaglutide.

Despite these successes, manufacturing costs are a barrier to accessibility. In the USA, the monthly cost of Wegovy (semaglutide) is in the range of $1000 to $1400 per month.^4^ This cost challenge can be partially attributed to the complexity and inefficiency of current peptide manufacturing technologies. GLP-1 analogues generally consist of >30 amino acids (Figure 1) and are produced through chemical synthesis, biological fermentation, or hybrid methods combining both approaches (Figure 2).^5^

**Figure 2:**
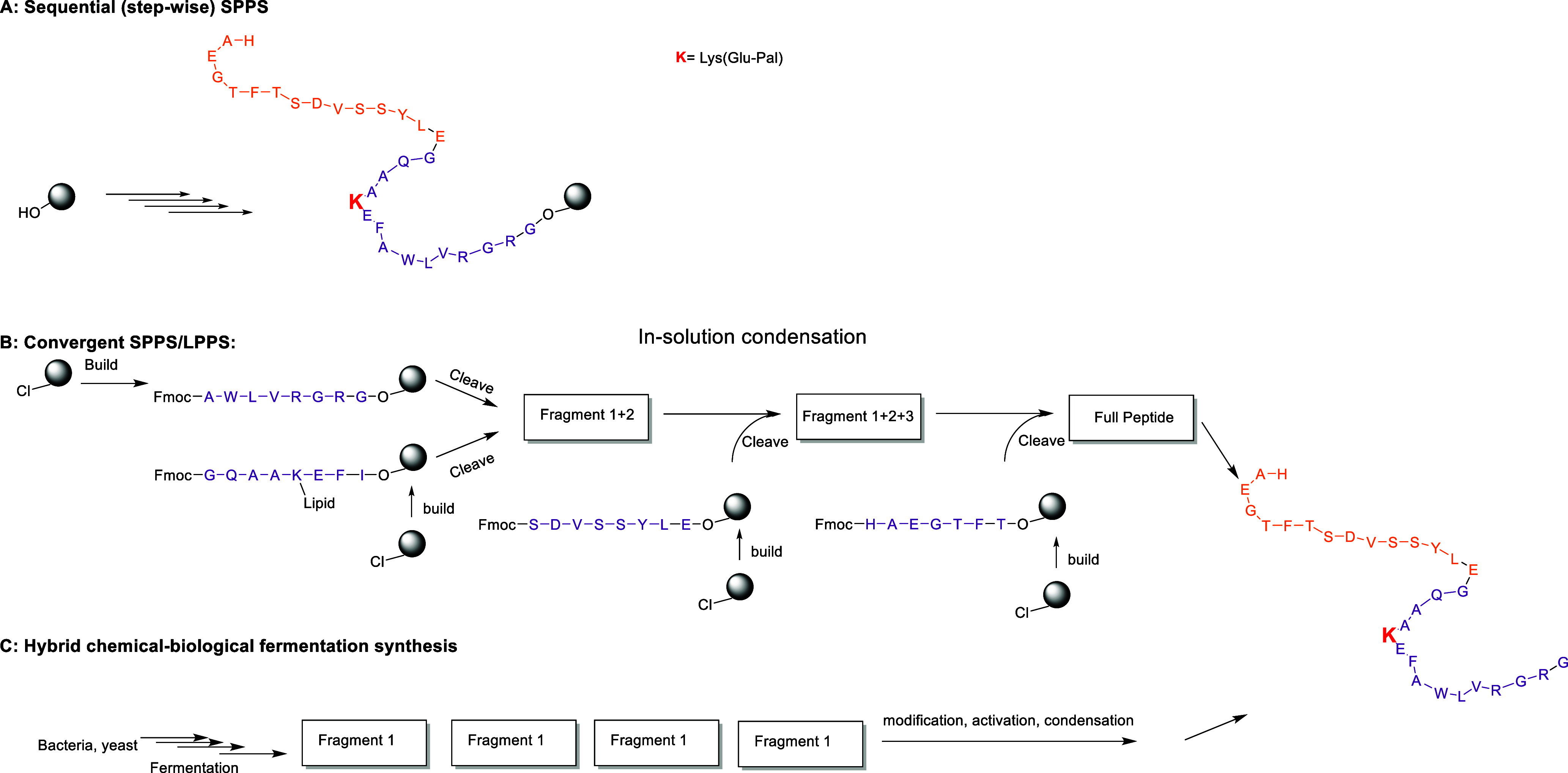
Representative synthetic routes to the GLP-1 analogue liraglutide: (A) sequential solid-phase peptide synthesis (SPPS), (B) convergent SPPS/solution-phase peptide synthesis (LPPS) approaches, and (C) hybrid recombinant–chemical synthesis strategies.

Chemical synthesis of GLP-1 analogues is normally carried out using sequential (stepwise) solid-phase peptide synthesis (SPPS, Figure 2A). Although this stepwise method remains the cornerstone of peptide chemistry, it is hampered by many factors including chain aggregation during elongation, as growing peptide sequences on resin tend to adopt β-sheet-like conformations.^6^ This aggregation reduces coupling efficiency, leading to incomplete reactions, sequence truncations, and increased side-product formation, which collectively diminish yield and purity. As a result, conventional sequential SPPS becomes inefficient and unsuitable for the large-scale manufacture of many peptides longer than 20 residues.^7^

Alternatively, for large peptides, convergent synthesis strategies are employed (hybrid SPPS/classical solution phase synthesis CSPS, Figure 2B), in which shorter protected peptide fragments (typically 5–10 residues) are first assembled by SPPS and subsequently joined through CSPS.^8–10^ However, they require multiple intermediate steps, including fragment cleavage, purification, and condensation, each of which reduces the overall yield. The use of highly reactive or unstable resins (e.g., CTC) can further contribute to yield losses caused by premature cleavage. Additionally, the protected fragments released from the resin must be sufficiently soluble for subsequent in-solution condensation, while the extra purification and ligation steps increase solvent consumption and environmental burden, ultimately reducing the scalability and sustainability of convergent synthesis methods.^11^ Other chemoenzymatic approaches have also been reported, in which peptide fragment coupling is performed enzymatically using a ligase to synthesize liraglutide. While this method provides a greener alternative for the condensation step, it still retains the same limitations in the remaining steps of the process.^12,13^

In addition to chemical methods, recombinant approaches have also been employed to produce GLP-1. These strategies can yield either the full-length peptide or defined fragments.^14^ Although recombinant expression overcomes certain limitations of SPPS, it presents its own set of challenges, including low expression efficiency, scalability issues, batch-to-batch variability, time-consuming processes, the need for post-translational modifications, and complex downstream purification.^5^

To overcome these limitations, we employed the OBIMAP (one-bead interchain multipeptide assembly platform), previously developed by our team, which is an advanced SPPS strategy enabling the synthesis of long peptides and challenging cyclic peptide architectures.^15,16^ Herein, we demonstrate its capability to the efficient synthesis of peptides longer than 30 residues, exemplified by liraglutide, a GLP-1 analogue. The performance of OBIMAP was benchmarked against conventional sequential SPPS for the preparation of the Fmoc-protected main peptide chain of liraglutide at scale 0.1 mmol (**10**, Figure 3). This comparative analysis highlights the superior performance of the OBIMAP in terms of yield and purity (Table 1). We also report the optimization of the on-resin lipidation step which successfully incorporated the lipid moiety to the Lys side chain.

**Figure 3:**
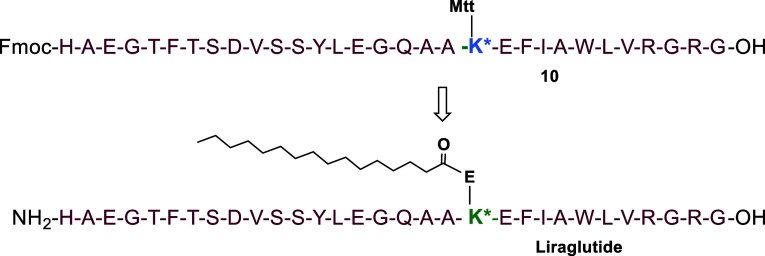
Structural sequence of liraglutide and its precursor, peptide **1**.

**Table 1:** Comparison of the 0.1 mmol Synthesis of Peptide **10** Using (1) Fully Automated Sequential SPPS, (2) Manual OBIMAP (RSE), (3) Partially Automated OBIMAP (RSE), and (4) Partially Automated Merrifield2.0 (PCL)

trial	protocol	use of automated synthesizer	total waste volume (L)	peptide **10** purity (%)^a^	crude recovery (%)	time of synthesis (h)
1	sequential Merrifield 1.0	fully automated	∼1.2	∼25	37	8
2	RSE OBIMAP	manual	∼1	∼50.4	76	41
3	RSE OBIMAP	2/9 automated steps	∼1.1	∼36	44	23
4	PCL OBIMAP	2/9 automated steps	N.A.	<10	N.A.	23

a Product purities were determined from UV–visible traces and analytical chromatograms of the cleaved crude peptides (Figures S5–S7, Supporting Information).

**Table 2:** Calculated Loading for Each Experimental Trial in Scheme 2 and the Corresponding Main Conclusions

trial	loading (mmol/g)	conclusion
A	1.33655	full occupancy
B	1.16957	binding sites available after Gly loading are >50%. Means that pNZ–Cl occupies <50%
C	1.40733	pNZ removal is efficient, Fmoc-Gly-active ester is more reactive than pNZ–Cl
D	0.65404	Fmoc-Gly-active ester is more reactive than pNZ–Cl
E	0.99052	pNZ–Cl occupies <50%
F	0.33540	loading of Fmoc-Gly-OH varies from batch to batch
G	0.89044	**double incorporating pNZ after Gly is more efficient in achieving the desired occupancy**

On the other hand, an initial 1 mmol scale-up trial was also attempted, but the crude purity was similar to that obtained with sequential synthesis at the same scale, due to method-related impurities that became more pronounced at this scale (Figure 5), as will be discussed during the manuscript. Scale-up remains an area for future optimization and validation.

## 2 Results and Discussion

Several approaches have been reported for the traditional sequential SPPS of liraglutide (Figure 2A), employing orthogonally protected lysine residues (e.g., Alloc, ivDde, or Mtt) to allow subsequent palmitoyl conjugation, or by introducing the Lys–Glu–palmitoyl moiety during peptide elongation.^6,17–19^ Reported outcomes vary widely, with crude purities ranging from 27% and overall yields from 220 mg of liraglutide API per 50 g of Wang resin^18^ to approximately 31% purity with recoveries as low as 8.6%. These values were improved to about 50% purity and 18% yield through the addition of inorganic salts such as MgCl_2_ or CaCl_2_, which help reduce peptide aggregation.^6^

OBIMAP integrates the strengths of sequential and convergent SPPS into a single on-resin platform. It enables full chain assembly directly on solid support, eliminating repeated cleavage and ligation steps while reducing aggregation-induced defects. The core innovation lies in synthesizing fragments in parallel on the same resin bead, followed by selective deprotection and in situ assembly. This strategy improves chain elongation process, translating into enhanced yield and purity.

In this strategy, two medium-length peptide segments were synthesized directly on the resin support (Scheme 1). Fragment 1 (residues 16–31, purple in Scheme 1) was anchored via the carboxyl group of Gly 31, while fragment 2 (residues 1–15, orange in Scheme 1) was immobilized through the side chain of Glu 15. Both fragments were subsequently joined by coupling the N-terminus of residue 16 to the C-terminus of residue 15.

**Scheme 1:**
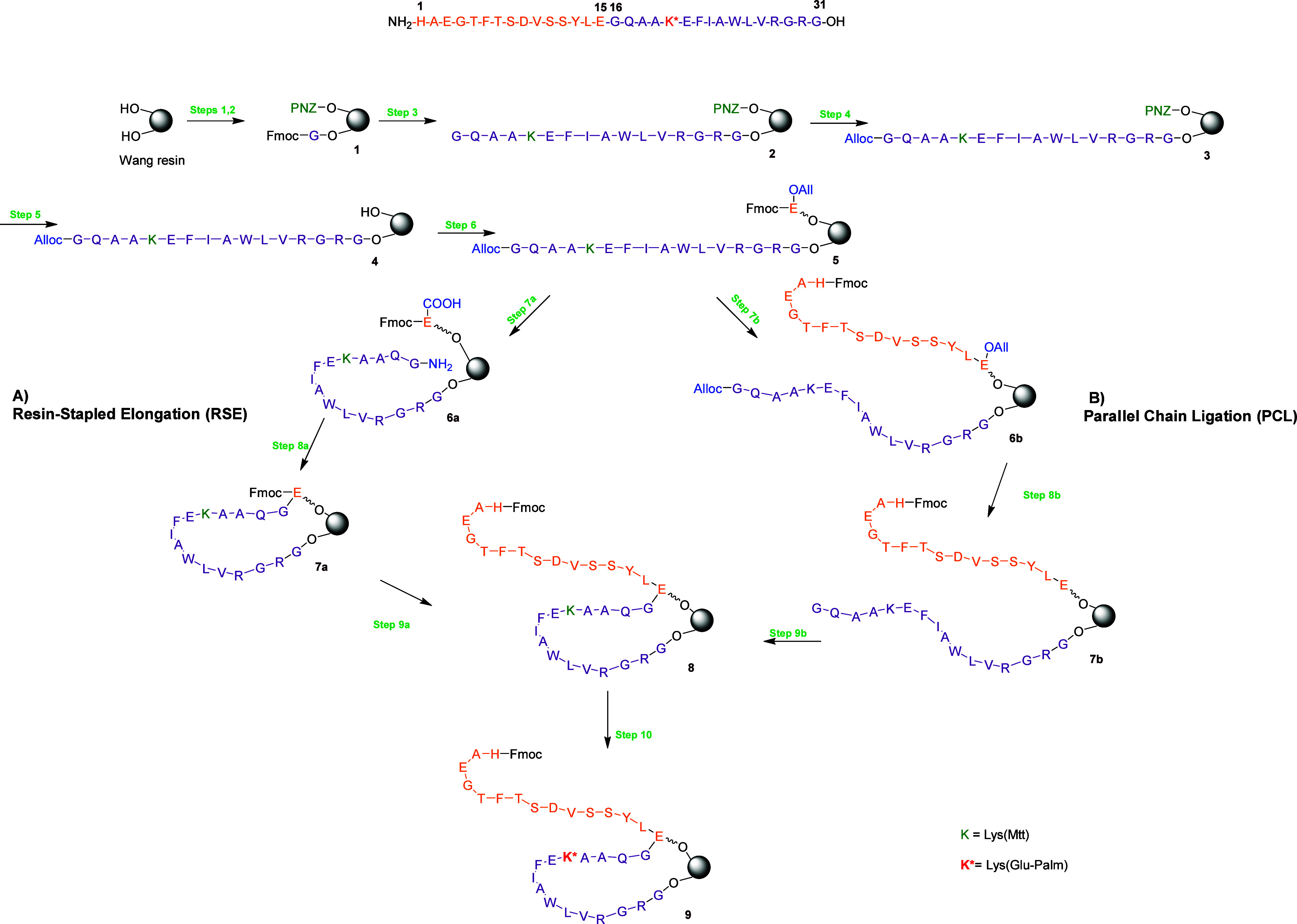
Synthetic Routes to the Liraglutide Main Peptide Chain via the OBIMAP Platform, Showing (A) resin-stapled elongation (RSE) and (B) parallel chain ligation (PCL)^a^

a Step 1: AA (0.5 equiv), DIC (0.5 equiv), DMAP (0.05 equiv) in DCM (*equivalents relative to binding sites; for subsequent steps, equivalents are relative to the reaction scale*). Step 2: pNZ–Cl, DIPEA in DCM. Steps 3, 7b, and 9a: standard peptide chain elongation using AA:DIC:OxymaPura, with Fmoc deprotection using piperidine in DMF. Step 4: allyl chloroformate, DIPEA in DMF. Step 5: 3 M SnCl_2_ in DMF with 64 mM HCl. Step 6: Fmoc-Glu-OAll anchored to the resin via its side-chain carboxyl group (3 equiv DIC, 3 equiv DMAP, 0.1 equiv). Steps 7a and 8b: [Pd­(PPh_3_)_4_] (0.1–0.2 equiv), PhSiH_3_ (10–20 equiv) in DCM, 1 h. Steps 8a and 9b: DIC (5 equiv), OxymaPure (5 equiv), 9–16 h. Step 10: Palm-l-Glu-OH (5 equiv), DIC (5 equiv), OxymaPure (5 equiv), 2–16 h.

### 2.1 Resin-Stapled Elongation (RSE) vs Parallel Chain Ligation (PCL)

Two alternative fragment-assembly strategies were examined. The first, termed resin-stapled elongation (RSE), followed a *build–link–build* process: the C-terminal residues of both fragments were initially installed on the resin in approximately 1:1 ratio. Using orthogonal protection/deprotection steps, fragment 1 was extended up to residue 16, coupled to residue 15 of fragment 2, and then elongated further from residue 14 to residue 1 (Scheme 1a). This approach mimics sequential synthesis of the full chain but incorporates a midchain “stitching” step (Scheme 1-step 8a) to restrict conformational freedom and mitigate aggregation.

The second approach, designated parallel chain ligation (PCL), followed a *build–build–link* pathway, in which both fragments were elongated independently on the resin and subsequently cross-linked through orthogonal protecting groups (Scheme 1b).

To evaluate this strategy, trials were conducted on a 0.1 mmol scale using Wang resin capacity (1.48 mmol/g, supplier’s specifications) to assess both approaches. Reaction progress was monitored by mini-cleavage samples with subsequent LC–MS analysis after each critical step (i.e., step 8a). The resin was initially half-loaded with Fmoc-Gly-OH using diisopropylcarbodiimide/4-dimethylaminopyridine (DIC/DMAP), representing the C-terminal residue of fragment 1, while the remaining hydroxyl groups were temporarily protected with a pNZ group to reserve anchoring sites for fragment 2. Fragment 1 (residues Gly16–Gly31) was then assembled on the resin, after which the *N*-terminal Fmoc group was selectively replaced with an Alloc protecting group. Subsequent pNZ deprotection liberated the second half of the resin sites, which were then functionalized with Fmoc-Glu-OAll through its side-chain carboxyl group.

For the resin-stapled elongation (RSE) approach, the alloc group (at the N-terminus of fragment 1) and the allyl group (at the α-carboxyl of Glu15 from fragment 2) were simultaneously deprotected, enabling amide bond formation between the free N-terminal amine of fragment 1 and the constrained C-terminal carboxyl group of Glu15 tethered to a neighboring resin site. Coupling was performed under DIC/OxymaPure activation, representing the critical step of this strategy (Scheme 1, step 8a). Because the reaction occurred between near-equimolar quantities of the immobilized Glu carboxylate and the amine terminal, it proved the most time-intensive step. Initial conditions employed overnight coupling at room temperature, with further optimization trials discussed in Sections 2.3 and 2.4. A test cleavage confirmed the formation of the desired intermediate **7a** as the major product (Figure 4A).

**Figure 4:**
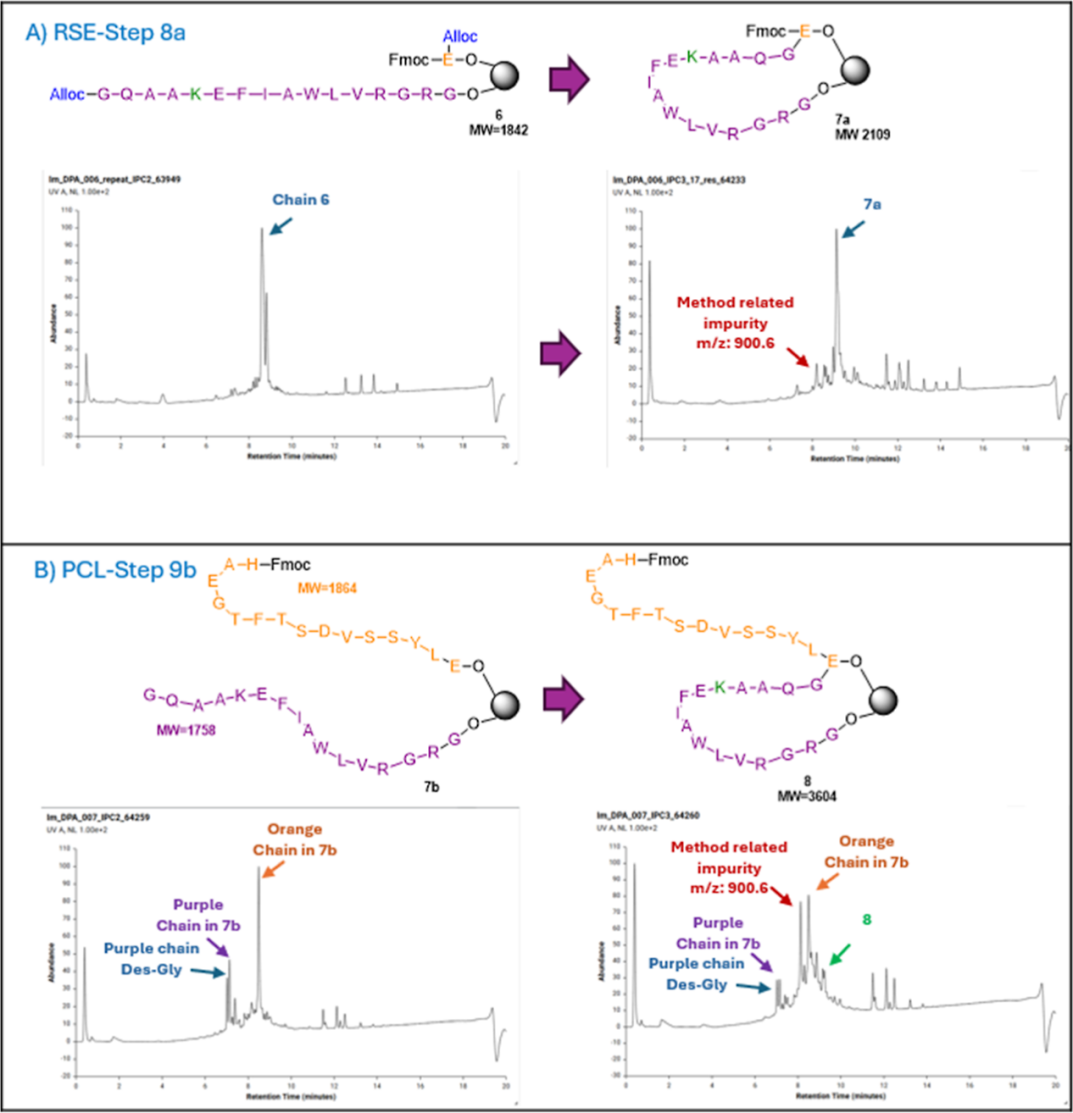
UV traces of LC–MS chromatograms of the fragment linking product via: (A) RSE approach (step 8a after 18 h) and (B) PCL approach (step 9b, after 18 h) (Figures S1–S4).

Subsequent chain extension of fragment 2 (residues Leu14–His1) delivered the full-length product 8, which was obtained in higher purity 50% compared with conventional stepwise SPPS elongation 25% (Table 1, entries 1 and 2).

In contrast, the alternative strategy, parallel chain ligation (PCL), in which both fragments were elongated independently prior to cross-linking, resulted in incomplete conversion, with unreacted starting fragments remaining. Although some target peptide **8** was formed, it exhibited lower purity (<10%) and generated additional byproducts (Figure 4B). These impurities were attributed primarily to incomplete couplings and capping events within fragment 1.

Initial results (Table 1) demonstrate the superiority of the RSE approach in terms of both crude purity and recovery when compared with PCL and conventional sequential SPPS. However, this improvement came at the expense of synthesis time. The manual RSE trial required approximately 5-fold longer reaction time (trial 2, Table 1).

In contrast, the partially automated trial, where an automated synthesizer was used to elongate both fragments in two of the nine total steps, reduced the increase in reaction time to approximately 3-fold (trial 3, Table 1). The reference sequential SPPS synthesis (trial 1, Table 1) was conducted fully on an automated synthesizer, apart from the first residue attachment, which was performed manually. Notably, solvent consumption was comparable across all approaches.

In all trials involving full-chain elongation using sequential SPPS, a byproduct corresponding to a His deletion was consistently observed. This occurred despite performing all His couplings with Fmoc-His­(Boc)-OH (rather than Fmoc-His­(Trt)-OH) and optimizing conditions on the synthesizer (50 °C for 10 min, compared to 90 °C for 2 min used for other amino acids, as recommended by the synthesizer’s manufacturer) (Figure 5a). Notably, this deletion was much lower in trials conducted using the RSE OBIMAP strategy (Figure 5c,e). This observation strongly suggests that the midchain “stitching” inherent to the RSE approach effectively mitigates one of the major drawbacks of conventional sequential SPPS, on-resin aggregation.

**Figure 5:**
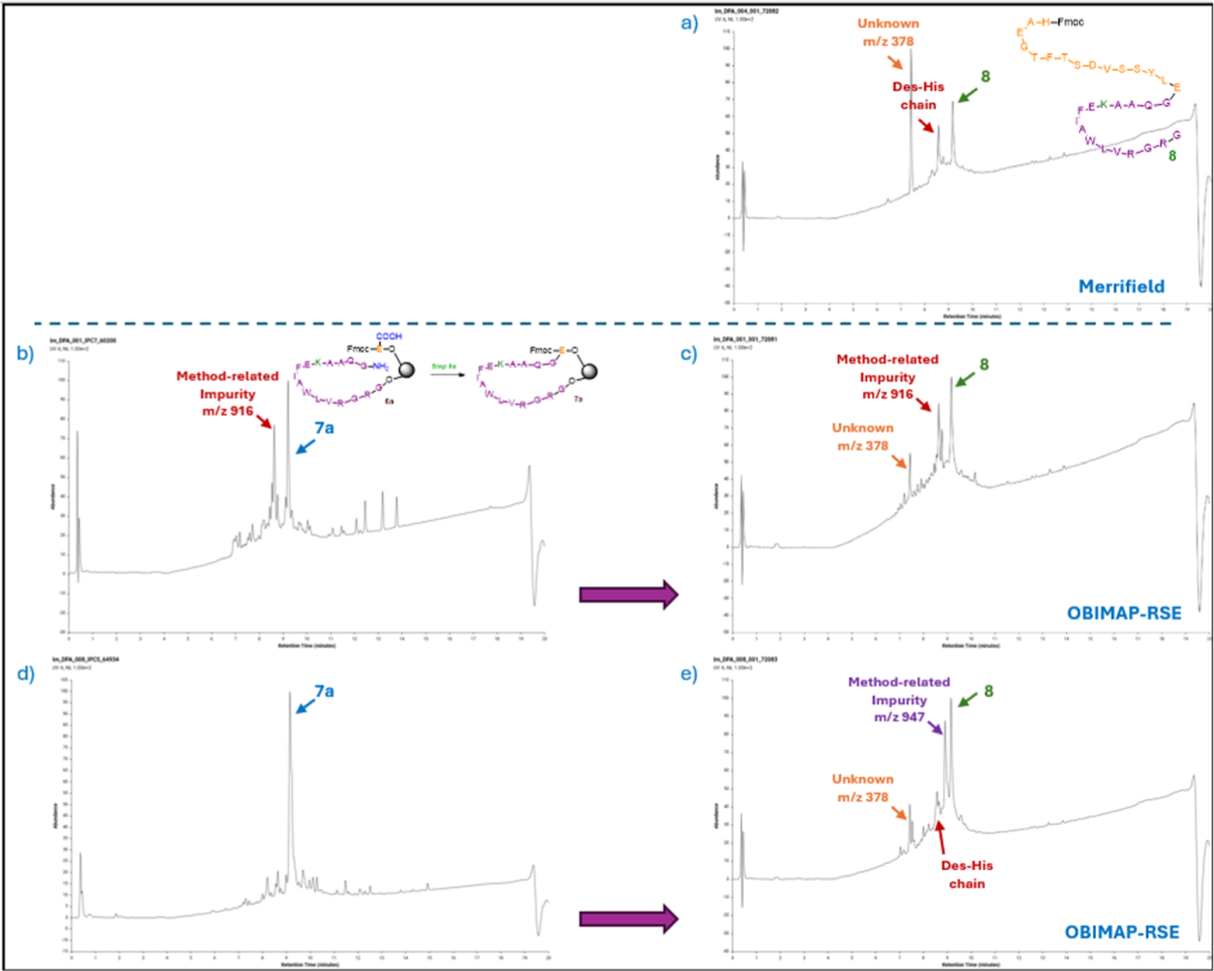
UV chromatograms (LC–MS traces) showing RSE method-related impurities observed after the on-resin cyclization step in RSE syntheses: (b) before and (d) after optimization of the loading protocol. Chromatograms after completion of the synthesis are shown for (a) sequential SPPS (automated synthesis), (c) RSE (manual synthesis), and (e) RSE (partially automated synthesis) (Figures S5–S9).

However, one or more unidentified peaks (*m*/*z* 915.8, 900.8, and 946.6) were detected at varying intensities in the RSE OBIMAP trials. These peaks are suspected to correspond to capped forms of one of the peptide chains, although they do not match any of the expected capped fragments. The peaks at *m*/*z* 915.8 and 900.8 are most likely associated with chain 1 (the purple chain), as they first appear after the on-resin cyclization step (step 8a) and persist throughout the synthesis, as shown in the final crude product of trial 2 (Table 1 and Figure 5b,c). Their formation is likely due to a slight imbalance in the abundance of the two reactive fragments, resulting in partial capping of the excess fragment, or to incomplete cyclization during the on-resin coupling of residues 15 and 16 (steps 8a/9b). Importantly, chromatographic analysis of the RSE synthesis showed no evidence of free fragment 2 at completion, indicating that all Glu residues (residue 15, the first residue of fragment 2) has been successfully coupled to the N-terminus of fragment 1.

To address this issue, loading optimization experiments were performed, which successfully reduced the formation of these species at the small scale (Figure 5b,d). These results will be discussed in the following section. The unidentified peak at *m*/*z* 946.6, observed in the final product of one of the trials (Table 1, trial 3; Figure 5e), may be associated with fragment 2, as it appeared following the elongation of the second chain, where the loading had been optimized and the other two byproducts (*m*/*z* 915.8 and 900.8) were effectively mitigated. These byproducts were observed in the PCL approach and became more pronounced when the stitching step (8a) in the RSE approach was performed under microwave heating in the synthesizer.

It is noteworthy that a secondary peak, eluting adjacent to fragment 1 (chain 6, Figure 4a), was consistently observed in all samples prior to pNZ removal. This species exhibited a mass ion of +106 Da relative to the desired product, likely resulting from partial detachment of the pNZ group during the mini cleavage with TFA, generating an iminoquinone methide (IQM) intermediate that is believed to cause the peptide chain alkylation. Importantly, this byproduct was restricted to mini-cleavage analyses and was absent in the bulk peptide, where complete pNZ deprotection with SnCl_2_ without requiring scavengers.

Despite the presence of these byproducts, OBIMAP-RSE delivered higher overall yield and purity of the target product compared to conventional sequential synthesis at the 0.1 mmol scale. As solvent consumption and waste generation were comparable in both approaches, this improvement clearly demonstrates the superior performance of OBIMAP-RSE over sequential synthesis at this scale. However, the extended synthesis time represented a notable limitation. Consequently, subsequent optimization efforts (Sections 2.6) focused on reducing the overall synthesis time of the RSE approach while addressing and mitigating byproduct formation.

### 2.2 Loading Optimization

To further investigate the origin of the unknown byproducts observed in the above trials, whether arising from a competing reaction or from excess fragment 1, we evaluated the loading protocol. Although achieving an exact 1:1 fragment ratio is practically challenging, we anticipated that an initial equimolar approach would be sensible. Scheme 2 outlines the experimental design used to assess loading efficiency, which was subsequently quantified by UV–visible spectrophotometry (see Table 2).

**Scheme 2:**
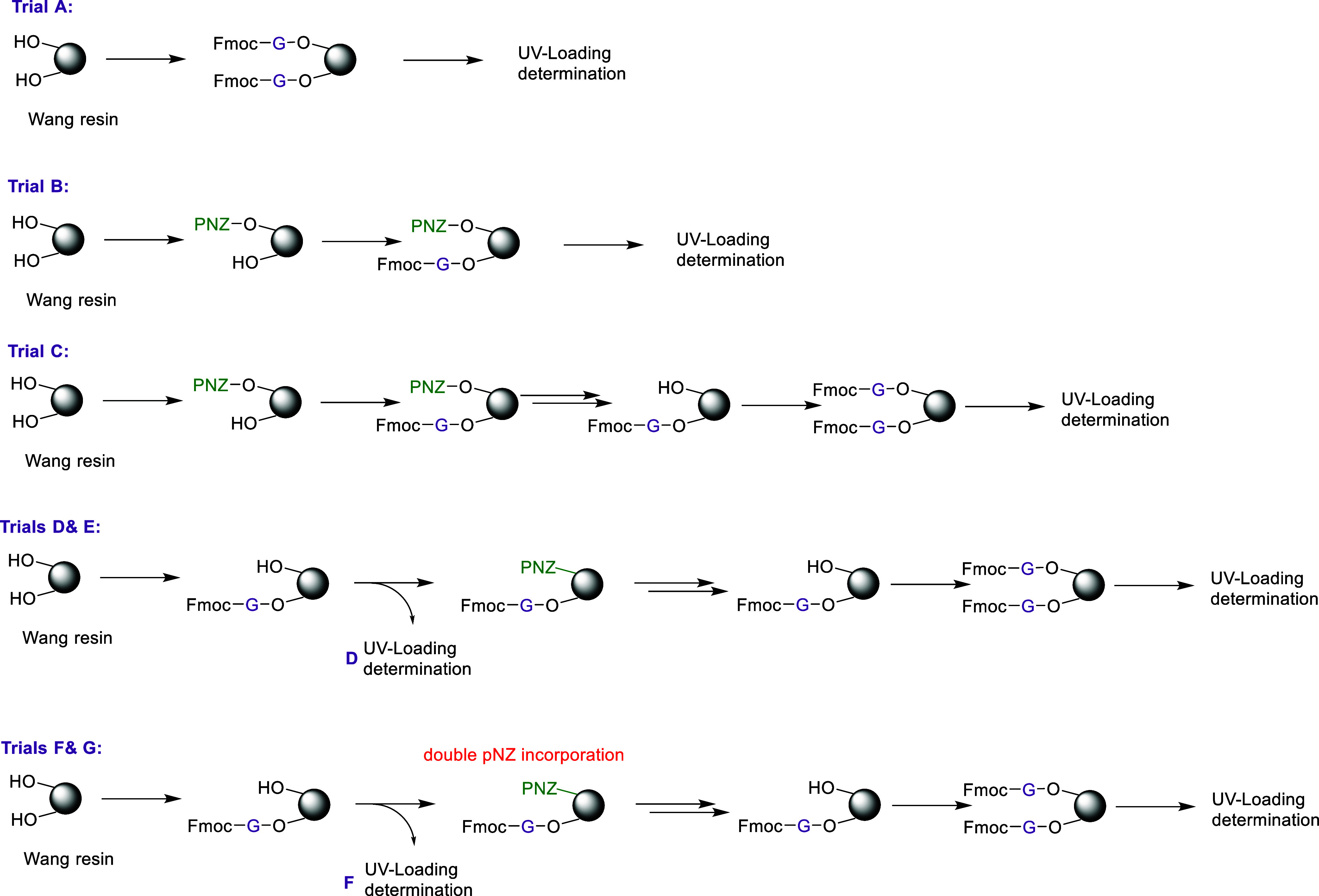
Assessment of Binding Site Occupancy Efficiency on Resin, Quantified by UV–Visible Spectrophotometry

The loading efficiency of Wang resin (1.48 mmol/g) was evaluated using different incorporation strategies of Fmoc-Gly-OH and *p*-nitrobenzyl (pNZ) groups. In sample A, the resin was fully loaded with Fmoc-Gly-OH using three equivalents under standard DIC/DMAP activation. Samples B and C examined partial site occupancy, initially blocking half of the resin sites with pNZ–Cl (0.5 equiv) followed by excess Fmoc-Gly-OH. In sample C, the reserved pNZ sites were subsequently deprotected and coupled with excess Fmoc-Gly-OH to achieve full loading. Conversely, samples D and E tested the reverse order, introducing Fmoc-Gly-OH first, followed by pNZ–Cl; in sample E, the pNZ-protected sites were later deprotected and coupled with excess Fmoc-Gly-OH. In samples C, E, and G the resins were acetylated after incorporation of both components and prior to pNZ removal. To prepare the samples for the UV experiment, they were treated with 20% piperidine in dimethylformamide (DMF) to remove Fmoc groups, the deprotection solution was then filtered and diluted in ethanol, and the released chromophore was quantified at 301 nm using UV–visible spectrophotometry, with pNZ not contributing to the signal.^20^

Comparing trials A–G (Table 2) revealed that introducing 0.5 equiv of pNZ–Cl first (trial B) occupied less than half of the available binding sites. In contrast, removal of pNZ and subsequent replacement with excess Fmoc-Gly (trial C) was quantitative, effectively freeing and refilling the sites. Because introducing pNZ first did not achieve the desired 50% occupancy, the sequence was reversed. Introducing 0.5 equiv of Fmoc-Gly-OH first (trial D) successfully achieved approximately 50% site occupancy. However, subsequent addition of excess pNZ–Cl and its replacement with excess Gly did not lead to full occupancy, likely due to limited pNZ–Cl reactivity, as also observed in trial B. Trials F and G showed some variability between batches, but overall confirmed that introducing the amino acid first, followed by double pNZ incorporation, yielded the most consistent and near-equimolar loading of the two fragments. This sequence, where fragment 1 (Gly) was slightly more abundant than fragment 2 (temporarily protected by pNZ), explained the observation of fragment1-related byproduct observed in the previous trials, and provided better control over site occupancy to minimize byproducts, and was therefore adopted in all subsequent trials (Figures 4d and 5b).

### 2.3 Optimization of On-Resin Cyclization Step (at Room Temperature)

To reduce the long synthesis time of the RSE approach, the on-resin cyclization step (step 8a, Scheme 1), the most critical and time-consuming stage of the synthesis, was monitored for completion over defined time intervals. Cyclization was initiated by adding DIC and OxymaPure (5 equiv each relative to chain loading) and agitating the resin at room temperature. At each time point, small resin samples were withdrawn, thoroughly washed, mini-cleaved, and the released peptide mixture analyzed by LC–MS. The conversion of the cyclized product **7a** relative to the starting peptide **6a** was determined from the 210 nm UV integrals. Maximum conversion (∼90%) was observed after 9 h (Table 3). This experiment demonstrated that the overall synthesis time could be reduced by 6 h, resulting in a total duration of 17 h for trial 3 (Table 1), approximately twice the time needed for the sequential synthesis in trial 1, while also providing improved purity and recovery.

**Table 3:**
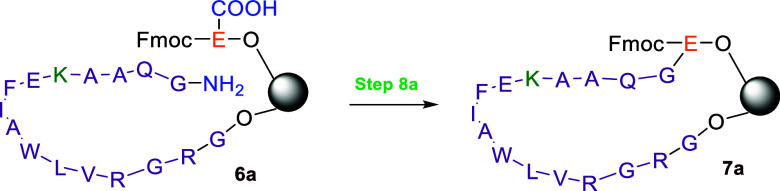
Time-Dependent Ratio of Cyclized Product **7a** to Starting Peptide **6a**, Determined from LC–MS UV Peak Areas

time (h)	pep **7a**/pep **6a** ratio (peak area at UV 210 nm)
2	3:4 (∼42%)
4	3:2 (∼60%)
9	∼90%

### 2.4 Modified RSE Route: Evaluation of Step Reduction and Automation Attempts

To further enhance the synthesis time of the RSE strategy, we investigated a modified route that reduces the number of steps required to assemble the Fmoc-protected main peptide chain (compound **8**, Scheme 3) from nine to seven. This modification allows evaluation of full automation by eliminating the allyl protection step, which otherwise requires prolonged handling of highly reactive allyl chloroformate (or diallyl dicarbonate) in solution, and by avoiding the manual incorporation of the second-chain amino acid (step 5, Scheme 1) midway through the sequence. By streamlining these operations, the synthesis can proceed seamlessly without interruptions between automated and manual steps.

**Scheme 3:**
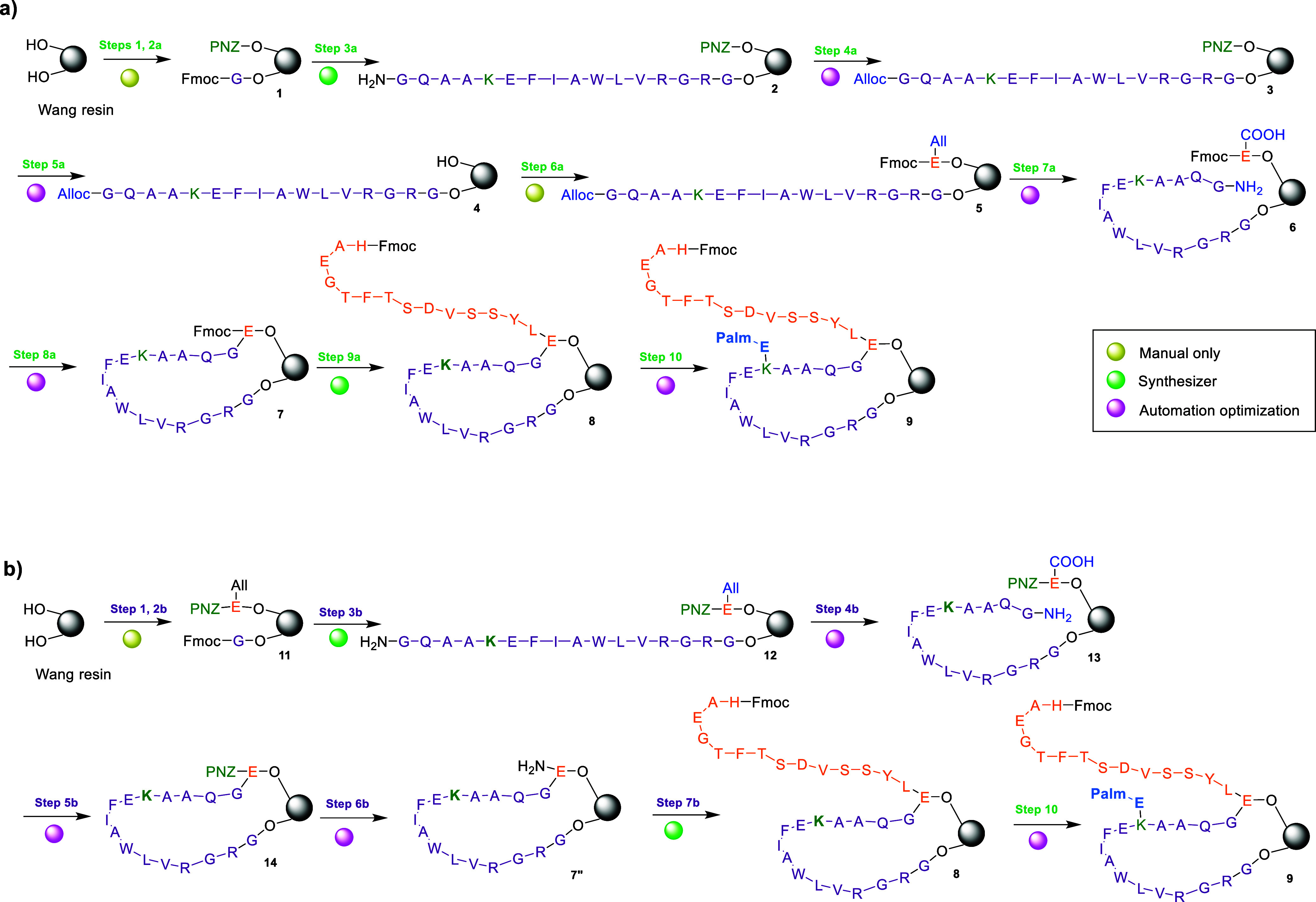
Comparison of the Original RSE Synthetic Route (a) with the Modified RSE Route (b) for the Synthesis of Liraglutide, Highlighting the Reduction in Steps and Streamlined Automation

In the original RSE route (Scheme 3a), nine steps were required to obtain compound **8**. Of these, three steps (1, 2a, and 6a), involving ester bond formation, could only be performed manually, namely the initial incorporation of the first residue or protecting group into Wang resin. Two steps (3a and 9a), corresponding to conventional fragment elongation of fragment 1 (purple chain, Scheme 3) and fragment 2 (orange chain, Scheme 3), were compatible with automated synthesis. The remaining four steps, Alloc protection (4a), pNZ removal (5a), Alloc deprotection (7a), and fragment coupling (8a), were considered for potential automation trials. These possibilities were further investigated in the modified RSE strategy (Scheme 3b).

The modified route relies on the prior preparation of pNZ–Glu–OAll, which is introduced at half of the resin binding sites to reserve positions for fragment 2 elongation. In this design, Glu16 (the first residue of fragment 2) is anchored through its side chain, while its α-carboxyl is protected with an allyl group to facilitate fragment linking on later steps. Overall, the modified approach reduces the synthesis to seven steps, with only two manual ester-formation steps retained at the beginning of the sequence. Two peptide elongation steps were carried out on the automated synthesizer, while the remaining three steps (Alloc removal, pNZ removal, and fragment linking) were examined for automation compatibility.

A preliminary trial of fully automating the modified RSE route was conducted. The on-resin cyclization step (Figure 6a) was performed at 50 °C for 60 min on the synthesizer, resulting in a good conversion (∼70%) to the desired cyclized product. This was a promising outcome, indicating a potential reduction in the duration of this typically challenging step. The observation of the cyclized intermediate as the major species also confirmed that the preceding automated allyl removal step was successful. Although the deallylation was carried out using 0.1 equiv of Pd catalyst in dry DMF instead of dichloromethane (DCM), it appeared to proceed effectively. The presence of residual compound **13** (Figure 6a) suggests that either the deallylation was incomplete or that the automated cyclization (linking) step required a longer reaction time. Nevertheless, both results were encouraging for further optimization of the automated process.

**Figure 6:**
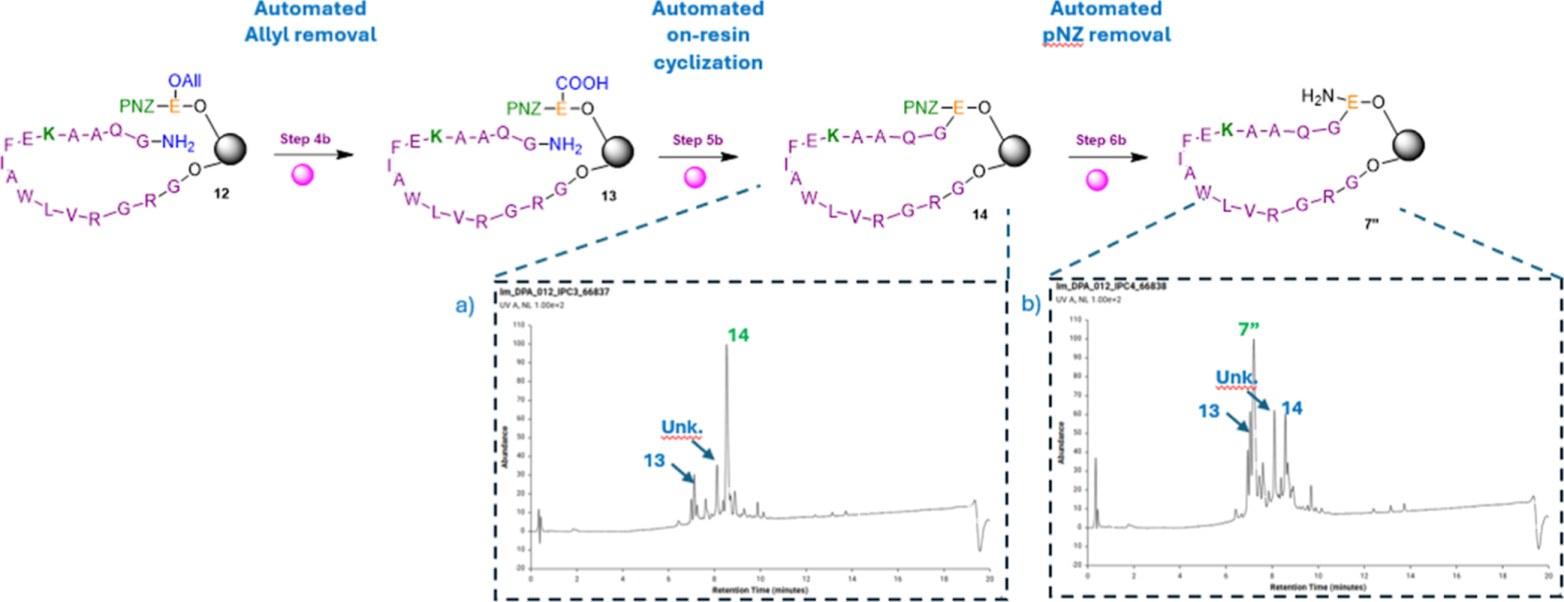
Automated trials of the modified RSE route. (a) On-resin cyclization performed at 50 °C for 60 min on the synthesizer, showing formation of the desired cyclized product (∼70%) and residual compound **13**. (b) Automated pNZ removal using 2 M SnCl_2_ 2 × 30 min, resulting in conversion of compound **14–7** (∼60%) with traces of unreacted intermediate 13 (Figures S10–S11).

Additionally, the pNZ removal trial was performed on the synthesizer using 2 M SnCl_2_, leading to a good conversion of compound **14** to **7″** (Figure 6b), with an estimated yield of approximately 60%. Some unreacted compound **13** carried over from earlier steps was still detected. Despite the incomplete conversions, these results provided a strong foundation for continued development toward fully automated synthesis with reduced overall processing time.

### 2.5 Automation Attempts of Alloc, Ally, pNZ Removal, and On-Resin Cyclization (Stitching) Step

Manual deprotection steps in OBIMAP-RSE were highly effective at room temperature. Alloc/allyl removal achieved complete conversion at room temperature using 0.1–0.2 equiv of Pd catalyst applied in two successive treatments. In contrast, automation consistently resulted in incomplete deprotection (∼35%) due to factors such as the synthesizer’s incompatibility with DCM, poor catalyst solubility in dry DMF, and accelerated catalyst oxidation at elevated temperatures. Analytical challenges in the modified RSE protocol further complicated monitoring, so Alloc removal was assessed indirectly via the subsequent cross-linking step (trials in Table S1). Similarly, pNZ removal was efficient manually, but the high SnCl_2_ concentrations required for the modified RSE protocol were incompatible with the synthesizer, and lower concentrations yielded only ∼60% deprotection (trials in Table S2). Consequently, both Alloc/allyl and pNZ deprotection steps were performed manually in all subsequent trials.

The on-resin cyclization step, critical for fragment linking, was the most time-consuming stage, requiring approximately 9 h at room temperature to reach ∼90% conversion. Microwave-assisted heating on the automated synthesizer reduced reaction time to 60 min at 50 °C but achieved only ∼70% conversion and was accompanied by method-related impurities (trials in Table S3). These results indicate that full automation of OBIMAP-RSE is not yet feasible; only conventional chain-elongation steps could be automated successfully. Partial automation reduced the total synthesis time from 23 to 17 h for the original RSE protocol, with the modified RSE protocol potentially shortening it further to 15 h, highlighting the need for further optimization of OBIMAP-specific steps.

### 2.6 On-Resin Lipidation

Palmitoylation is a critical step in the synthesis of liraglutide and is widely regarded as a challenging transformation. It is typically performed either as a postelongation modification at the end of peptide chain assembly, requiring overnight reaction,^13^ or through incorporation of a prepalmitoylated amino acid during peptide synthesis.^17^

After we completed the elongation of liraglutide’s precursor, peptide **10**, several trials were conducted to attach the lipid moiety to the side chain of Lys20 (purple chain) (Scheme 1-step10). These experiments were performed either manually at room temperature or via automated microwave-assisted double coupling at 90 °C. The results (Table 4) indicate that the automated conditions (at 90 °C) provided superior conversion efficiency (trial 1).

**Table 4:**
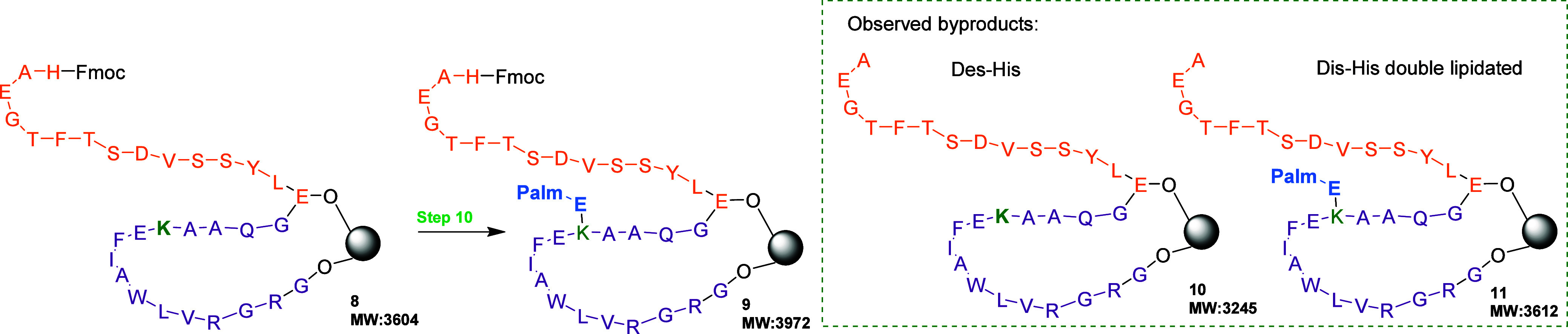
Summary of Lipidation Trials; Conditions and Outcomes for Liraglutide Synthesis (Figures S12–S16)

no.	source of reacting resin	purity of reacting resin	lipidation performed on	conditions Palm-l-Glu-OH/DIC/OxymaPure(step 10)	conversion to desired product
1	sequential SPPS	compound **8**/**10** = 4:3	synthesizer	5 equiv/5 equiv/5 equiv	9/11 (4:3), means complete lipidation
				90 °C, 2 × 2 min	
2	sequential SPPS	compound **8**/**10** = 4:3	manual	5 equiv/5 equiv/5 equiv	1st coupling <40% conversion
				RT, 2 h	2nd coupling >60%
				repeated 3 times	3rd coupling ∼100%
3	RSE	compound **8**, with unknow mass ion 916	manual	10 equiv/10 equiv/10 equiv	(∼90% conversion)
				RT, 18 h	
4	RSE	compound **8**, with unknow mass ion 916	manual	5 equiv/5 equiv/5 equiv	1st coupling
				RT, 2 h	2nd coupling
					3rd coupling (∼90% coversion)
5	RSE	compound **8**, with unknow mass ion 947	manual	5 equiv/5 equiv/5 equiv	1st coupling
				RT, 2 h	2nd coupling
					3rd coupling (∼90% coversion)

The lipidation step was initially attempted for 18 h at room temperature (Table 4, Trial 3), resulting in a conversion of around 90%. Subsequently, the reaction time was adjusted to 2 × 2 min at 90 °C on the synthesizer (Table 4, Trial 1) and 3 × 2 h for manual lipidation at room temperature (Table 4, Trials 2, 4, 5).

The resin in trial 1, derived from a fully automated sequential synthesis, contained a des-His byproduct in a 3:4 ratio relative to compound **8**. The lipidation product showed complete modification, with no unreacted starting chains detected. Although the mass of byproduct 10 indicates it corresponds to the des-His deletion sequence and has two free amine groups capable of reacting with the lipid functionality, only one peak corresponding to monolipidated byproduct was observed, and no double-lipidated byproduct was detected. This is likely due to the short reaction time or accessibility issues at the *N*-terminus of the main chain of the byproduct, rather than the efficiency of the lipidation conditions. Such accessibility limitations are also believed to be responsible for the formation of this byproduct in the first place, hindering the attachment of the terminal His residue to the main chain. Notably, this issue was observed only in samples produced via the sequential approach, as mentioned previously.

On the other hand, manual lipidation of a sample from the same origin as in trial 1 was performed at room temperature for 2 h (Table 4-trial 2). This initially yielded a mixture of all four compounds (**8–11**), with an estimated lipidation of slightly less than 40%. A second lipidation of the same sample increased the conversion to over 60%, and a third lipidation was required to achieve complete conversion. Also, the starting material used for the lipidation trials in entries 3–5, which originated from the RSE approach, contained an unknown byproduct with an *m*/*z* of 916. It is lipidation product was observed as well in the final product (mass ion 1099 observed in Figure S14). These results confirm that the lipidation conditions are effective, achieving a conversion of >90%. While the unknown impurity with an *m*/*z* of 947 in the starting material used in entry 5 does not appear to contain a Lys (Mtt) residue, only the desired lipidated product **9** was observed when the same successful conditions were applied.

Our method enabled efficient, faster postsynthetic modification despite the increased complexity associated with the simultaneous presence of two distinct peptide chains. This result highlights the flexibility of the platform, albeit requiring higher reagent equivalents.

### 2.7 A 1.0 mmol Scale-Up Trial

Although scale-up was beyond the scope of this manuscript, a single attempt was made to scale the synthesis to 1 mmol using the optimized conditions developed for the 0.1 mmol scale. This scale-up proved challenging. Increased formation of RSE-related byproducts at this scale resulted in crude product purity comparable to that obtained by conventional methods, while the overall synthesis time remained longer. These factors currently limit the applicability of the technology to small-scale syntheses.

Further investigation and optimization are therefore planned as part of future work. These include the evaluation of preloaded linkers to enable exact equimolar fragment loading rather than near-equimolar ratios, the incorporation of MgCl_2_ salts during chain elongation steps, and the exploration of alternative coupling reagents with slower kinetics for the stitching steps.

## 3 Conclusion and Future Work

This study demonstrates the successful application of OBIMAP-RSE, an enhanced SPPS platform to the synthesis of aggregation-prone peptides such as liraglutide. Through systematic comparison with conventional sequential SPPS, OBIMAP achieved considerable improvements in yield, crude purity, and indirectly process sustainability at scale 0.1 mmol.

The resin-stapled elongation (RSE) strategy, in particular, effectively mitigated aggregation-related sequence deletions, most notably histidine loss, by introducing a midchain stabilization step that maintained high coupling fidelity. The main limitation at this scale is the longer synthesis time compared to the sequential synthesis. Optimization of key steps, including on-resin cyclization and pNZ/Alloc deprotection, further streamlined the process, while partial automation successfully reduced synthesis time without compromising quality. Although full automation remains an area for continued development, the modified RSE route shortened synthesis duration by more than half compared to the original protocol and produced peptides with higher structural integrity and reproducibility.

These findings demonstrate that OBIMAP preserves the core principles of the original Merrifield solid-phase peptide synthesis while extending its capabilities to the synthesis of long-chain therapeutic peptides. In principle, the approach requires no fundamental modifications to existing manufacturing platforms, making it well positioned for seamless integration into current industrial processes. However, further efforts are required to translate the observed outperformance over conventional sequential synthesis from small-scale experiments to larger scales. Subsequent studies will then be needed to rigorously benchmark OBIMAP against other established strategies for the synthesis of long and synthetically challenging peptides.

## 4 Materials and Methods

Iris Wang resin (1.48 mmol/g, supplier’s specification), resins were used for all syntheses. All reagents and solvents were obtained from commercial suppliers and were used without further purification unless otherwise stated. Chromatographic separation (uPLC) and mass spectrometric analysis were conducted using an Agilent Infinity II 1260 UPLC-MSD-XT system equipped with a positive electrospray ionization (ESI^+^) source. The mobile-phase gradient ranged from 5% to 95% solvent B over 20 min, where solvent A was water with 0.1% formic acid and solvent B was acetonitrile with 0.1% formic acid. Separation was performed on a Thames Restek Raptor C18 column (2.7 μm, 3.0 × 100 mm). Liberty 2.0 BlueTM automated microwave-assisted peptide synthesizer (CEM) was used for peptide chains elongation.

### 4.1 Incorporation Procedure

First amino acids were incorporated onto Wang resin using DCM. Resin was swelled in DMF for 5–10 min. The Fmoc-amino acids (with the required number of equiv) were dissolved in a minimum amount of the DMF (0.5 mL/100 mg resin). *N*,*N*-diisopropylcarbodiimide (DIC) (1 equiv wrt amino acid) and DMAP (0.1 equiv) were then added to the solution, which in turn was added to the previously swelled resin and allowed to react for 1 h under mechanical shaking. Finally, the resin was washed twice with DCM and dried over vacuum.

### 4.2 Peptide Synthesis

Peptides were synthesized following the standard methodology performed in our laboratory (3 equiv of Fmoc-AA-OH, 3 equiv of OxymaPure, 3 equiv of DIC) in DMF and then shaking for 1 h. Fmoc was then removed using 20% piperidine in DMF for 1 + 7 min. All Arg and the residue that comes after were double coupled to ensure complete coupling.

### 4.3 Allyl/Alloc Protection and Deprotection

For introduction of the Alloc/OAll protecting groups, diallyl dicarbonate or allyl chloroformate (3 equiv) was dissolved in DCM and added to the peptidyl resin in the presence of DIPEA (6 equiv). The resulting slurry was agitated mechanically for 1 h to allow the reaction to proceed.

For deprotection of Asp­(OAll) or Lys­(Alloc), tetrakis­(triphenylphosphine)­palladium(0) (0.2 equiv) and triphenylsilane (20 equiv) in DCM were added to the peptidyl resin and the mixture was shaken for 1 h. The resin was subsequently washed twice with DCM, followed by three washes with *N*,*N*-diethyldithiocarbamate (0.02 M in DMF) to efficiently remove palladium residues.

### 4.4 pNZ Protecting Group Introduction/Removal

To protect half the binding sites of Wang resin with the pNZ group, the required equivalents of pNZ–Cl were dissolved in DCM and added to the resin in the presence of DIPEA (2 equiv). The reaction mixture was shaken for 1 h.

Removal of the pNZ protecting group was performed by treating the peptidyl resin with a SnCl_2_·2H_2_O solution (1–6 M, containing 64 mM HCl) at a ratio of 2 mL per 100 mg resin. The mixture was shaken for 30 min, and the treatment was repeated twice. The resin was then washed sequentially with DMF (×3), DMF/water (×3), DMF (×3), and DCM (×3).

### 4.5 pNZ–Glu–OAll Preparation

The precursor pNZ–Glu–OAll was synthesized separately on resin by incorporating Fmoc-Glu-OAll into Wang resin through its side chain. The α-amino group was deprotected using 20% piperidine in DMF and subsequently protected with a *p*-nitrobenzyl (pNZ) group using *p*-nitrobenzyl chloroformate and diisopropylethylamine (DIPEA). The protected amino acid was then cleaved from the resin, lyophilized, and characterized by LC–MS and NMR, and was used without further purification (Figures S17 and S18).

### 4.6 Mtt Protecting Groups Removal

To cleave Trt, 2% TFA in DCM was added to the peptidyl resin and allowed to react for 30 min under mechanical shaking. The resin was washed twice with DCM and then washed with 5% DIPEA in DCM to neutralize the protonated groups due to acid treatment. Finally, the resin was washed twice with DCM and dried over vacuum.

### 4.7 Interchain Assembly Reaction (On-Resin Cyclization)

Once the reactive amino acid side chains were prepared for amide bond formation, OxymaPure (5 equiv) and DIC (5 equiv) in DMF were added to the resin. The mixture was subjected to mechanical agitation for 16 h to promote on-resin cyclization via interchain amide bond formation.

### 4.8 Automatic Synthesis

In the automated SPPS workflow, at scale <0.4 mmol, amino acid coupling to the elongating peptide chain was carried out by introducing Fmoc-AA-OH (5 equiv, 0.2 M in DMF), OxymaPure (5 equiv, 0.5 M in DMF), and diisopropylcarbodiimide (DIC; 10 equiv, 0.5 M in DMF). Couplings were performed at 90 °C for 2 min (single coupling) or for 2 × 2 min (double coupling). *N*-terminal deprotection was accomplished by Fmoc removal using 10% v/v piperidine in DMF, followed by heating at 90 °C for 1 min.

For larger-scale syntheses, scale 1 mmol, the reagent concentrations and equivalents were adjusted as follows: Fmoc-AA-OH at 4 equiv (0.5 M in DMF), OxymaPure at 4 equiv (1 M), and DIC at 8 equiv (1 M). Microwave-assisted heating was applied for 10 min at 90 °C for amino acid coupling, and for 2.5 min at 110 °C for Fmoc removal. Coupling of histidine residues was conducted at 50 °C for 10 min to prevent side reactions.

### 4.9 Cleavage Protocols

The final synthesized peptide was cleaved from the resin using TFA/triisopropylsilane (TIS)/H_2_O (95:2.5:2.5) (1 mL/100 mg) under mechanical shaking for 1 h. Chilled diethyl ether was then added (5 times the cleavage solution volume), and the solution was kept in an ice bath for 30 min. The solution was then centrifuged for 5 min at 5000 rpm, and the supernatant was decanted. A new amount of the ether (5 times the cleavage solution volume) was added to repeat this step. Any remaining ether was dried under N_2_. Finally, the precipitate was dissolved in CH_3_CN–H_2_O (1:1). A small amount of the solution was injected into HPLC system to check the purity of the final product.

### 4.10 Loading Study

The peptide was synthesized on resin as shown in Scheme 2. Samples A, B, C, and D were synthesized on 50 mg resin each. Then 10 mg was taken for the loading determination part 2.

#### 4.10.1 Part 1: Sample Preparation

**A**: 50 mg of wang resin (0.074 mmol capacity, 1.48 mmol/g) were swelled in DCM, and Fmoc-Gly-OH (**66** mg, **3 equiv**) in DCM (250 μL) were added, followed with DMAP, and DIC (**34** μL). The reaction mixture was agitated for 1 h. Then washed with DCM 3×, dried and prepared for the following step.

**B**: 50 mg of wang resin (0.074 mmol capacity, 1.48 mmol/g) were swelled in DCM, and pNC-chloroformate (0.037 mmol, 0.5 equiv) and DIPEA (13 μL) and DCM 150 μL were added. The overall slurry was agitated for 1 h. The reaction mixture was then drained, washed with DCM 3×. Then Fmoc-Gly-OH (**33** mg, **1.50 equiv**) in DCM (250 μL) were added, followed with DMAP, and DIC (**17** μL). The reaction mixture was agitated for 1 h. Then washed with DCM 3×, dried and prepared for the following step.

**C**: 50 mg of wang resin (0.074 mmol capacity, 1.48 mmol/g) were swelled in DCM, and pNC-chloroformate (0.037 mmol, 0.5 equiv) and DIPEA (13 μL) and DCM 150 μL were added. The overall slurry was agitated for 1 h. The reaction mixture was then drained, washed with DCM 3×. Then Fmoc-Gly-OH (**33** mg, **1.50 equiv**) in DCM (250 μL) were added, followed with DMAP, and DIC (**17** μL). The reaction mixture was agitated for 1 h. Then washed with DCM 3×. SnCl_2_ solution (1 mL, 3 M) and agitated for 1 h. The resin was then washed with DMF 3×, DMF/H_2_O 3×, DMF 3×, DCM 3×. Then Fmoc-Gly-OH (33 mg, **1.50 equiv**) in DCM (250 μL) were added, followed with DMAP, and DIC (17 μL). The reaction mixture was agitated for 1 h. Then washed with DCM 3×, dried and prepared for the following step.

**D**: 50 mg of wang resin (0.074 mmol capacity, 1.48 mmol/g) were swelled in DCM, the Fmoc-Gly-OH (11 mg, **0.5 equiv**) in DCM (250 μL) were added, followed with DMAP, and DIC (**6** μL). The reaction mixture was agitated for 1 h. Then washed with DCM 3×, dried and prepared for the following step.

#### 4.10.2 Part 2: UV-Loading Determination

##### 4.10.2.1 Sample Preparation

Ten mg of each of the prepared loaded resins were weighed in a syringe, and 200 μL of the deprotection solution (20% piperidine/DMF) was added, and the sample was allowed to shake for 10 min. The filtrate was then collected in a 25 mL volumetric flask (another 200 μL was added to repeat this step). Finally, the volume was made up to 25 mL with EtOH. Samples were labeled **A**, **B**, **C**, and **D**.

##### 4.10.2.2 Blank Preparation

Four hundred microliters of the deprotection solution (20% piperidine–DMF) was transferred to a 25 mL volumetric flask, and the volume was made-up to 25 mL with EtOH.

##### 4.10.2.3 Standard Sample Preparation

About 3 mg of the corresponding/same Fmoc-Gly-OH being analyzed was transferred to a 25 mL volumetric flask; 400 μL of the deprotection solution (20% piperidine–DMF) was added, and the volume was made up to 25 mL with EtOH. It should be noted that Trp amino acid has a negligible absorbance at 301 nm. Thus, it is advisible to make it up in the calculations or use another amino acid as a standard with its corresponding molecular weight.

## Supplementary Material



## Notes

Chemical structures, HPLC chromatograms, mass spectrometry spectra, NMR (PDF)
