# Supplementary material for: Application of the OBIMAP (One-Bead Interchain Multipeptide Assembly Platform) to Long Peptide Synthesis: Liraglutide as a Case Study
Source: ACS Omega. 2026 Mar 25;11(13):21369–81. doi: 10.1021/acsomega.6c00648 (PMC13063175; doi:10.1021/acsomega.6c00648)
Supplement: Supplementary file 1 [file ao6c00648_si_001.pdf]

Application of the OBIMAP (One-Bead Interchain Multipeptide Assembly Platform) to Long Peptide Synthesis: Liraglutide as a Case Study

Danah AlShaer[1], Othman Al Musaimi[1,2,3*], and Daryl R. Williams[1,3*]

1. Department of Chemical Engineering, Imperial College London, London, SW7 2AZ, UK
2. School of Pharmacy, Newcastle University, Newcastle upon Tyne, NE1 7RU, UK
3. Orthogonal Peptides, London, SW7 2AZ, UK

*Corresponding authors

Table of content:

Figure	Page
**Figure S1**: UV traces (at 210nm) and mass spectra of LC-MS analysis of the cleavage product of peptidyl resin **6** Shown in Figure 3a	**2**
**Figure S2**: UV traces (at 210nm) and mass spectra of LC-MS analysis of the cleavage product of peptidyl resin **7a** Shown in Figure 3a	**4**
**Figure S3**: UV traces (at 210nm) and mass spectra of LC-MS analysis of the cleavage product of peptidyl resin **7b** Shown in Figure 3b	**5**
**Figure S4**: UV traces (at 210nm) and mass spectra of LC-MS analysis of the cleavage product of peptidyl resin **8** Shown in Figure 3b	**7**
**Figure S5**: UV traces (at 210nm) and mass spectra of LC-MS analysis of the crude product **8** Shown in Figure **4a**	**10**
**Figure S6**: UV traces (at 210nm) and mass spectra of LC-MS analysis of the crude product **8** Shown in Figure **4c**	**12**
**Figure S7**: UV traces (at 210nm) and mass spectra of LC-MS analysis of the crude product **8** Shown in Figure **4e**	**14**
**Figure S8**: UV traces (at 210nm) and mass spectra of LC-MS analysis of the crude product **7a** Shown in Figure **4b**	**17**
**Figure S9**: UV traces (at 210nm) and mass spectra of LC-MS analysis of the crude product **7a** Shown in Figure **4d**	**19**
**Figure S10**: UV traces (at 210nm) and mass spectra of LC-MS analysis of the mini-cleavage product of peptidyl resin **14**, shown in figure 5a	**20**
**Figure S11**: UV traces (at 210nm) and mass spectra of LC-MS analysis of the mini-cleavage product of peptidyl resin **7"**, shown in figure 5b	**22**
**Figure S12**: UV chromatograms (at 210, 280 nm) and mass spectra from the LC–MS analysis of the mini-cleavage product of the peptidyl resin obtained after the lipidation step (step 10), as reported in Table 4, entry 1.	**25**
**Figure S13**: UV chromatograms (at 210, 280 nm) and mass spectra from the LC–MS analysis of the mini-cleavage product of the peptidyl resin obtained after the lipidation step (step 10), as reported in Table 4, entry 2 (third coupling).	**27**
**Figure S14**: UV chromatograms (at 210, 280 nm) and mass spectra from the LC–MS analysis of the mini-cleavage product of the peptidyl resin obtained after the lipidation step (step 10), as reported in Table 4, entry 3	**29**
**Figure S15**: UV chromatograms (at 210, 280 nm) and mass spectra from the LC–MS analysis of the mini-cleavage product of the peptidyl resin obtained after the lipidation step (step 10), as reported in Table 4, entry 4 (third coupling).	**31**
**Figure S16**: UV chromatograms (at 210, 280 nm) and mass spectra from the LC–MS analysis of the mini-cleavage product of the peptidyl resin obtained after the lipidation step (step 10), as reported in Table 4, entry 5 (third coupling).	**33**
**Figure S17**: UV chromatograms (at 210) and mass spectra from the LC–MS analysis of pNZ-Glu(OAll)-OH	**36**

**Figure S18**: ^{1}H-NMR, ^{13}C-NMR, COSEY, HSQC NMR analysis of pNZ-Glu(OAll)-OH	**37**
**Table S1:** Alloc/Allyl removal trials in original and modified RSE: automation vs. manual (% conversion shown).	
**Table S2:** pNZ removal trials in original and modified RSE: automation vs. manual (% conversion shown).	
**Table S3:** On-resin cyclization (Step 5b) under RSE conditions showing reaction parameters and conversion to the cyclized product.	

Figure S1: UV traces (at 210nm) and mass spectra of LC-MS analysis of the cleavage product of peptidyl resin **6** Shown in Figure 3a

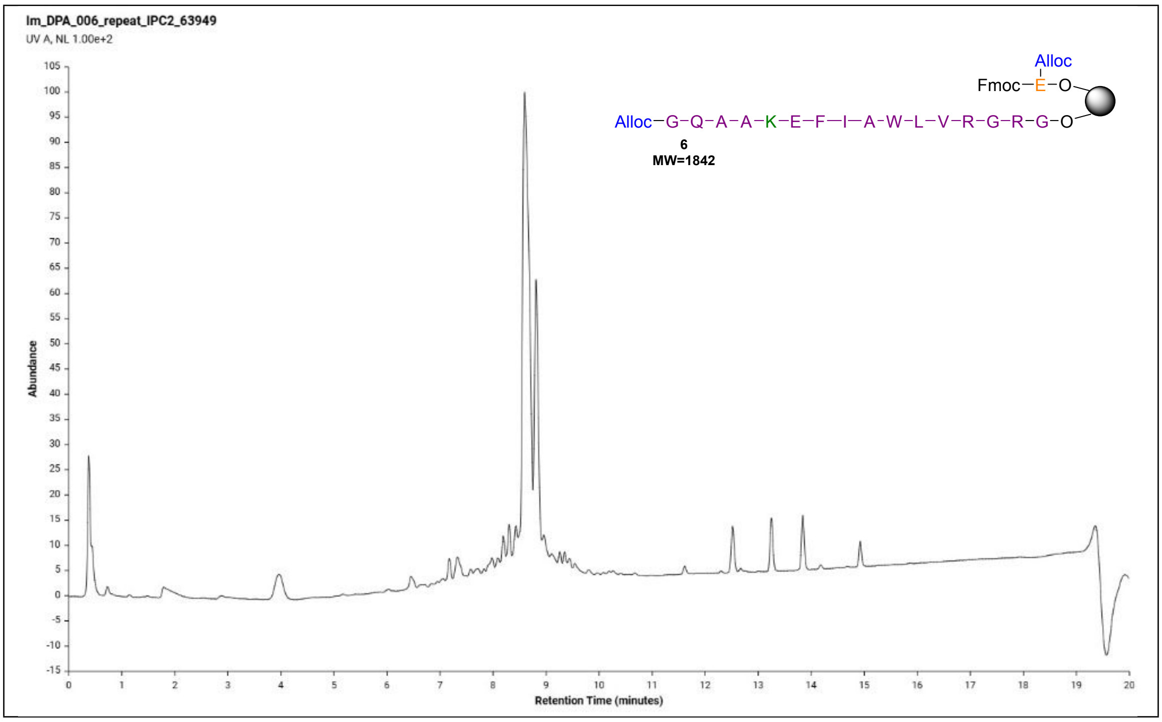

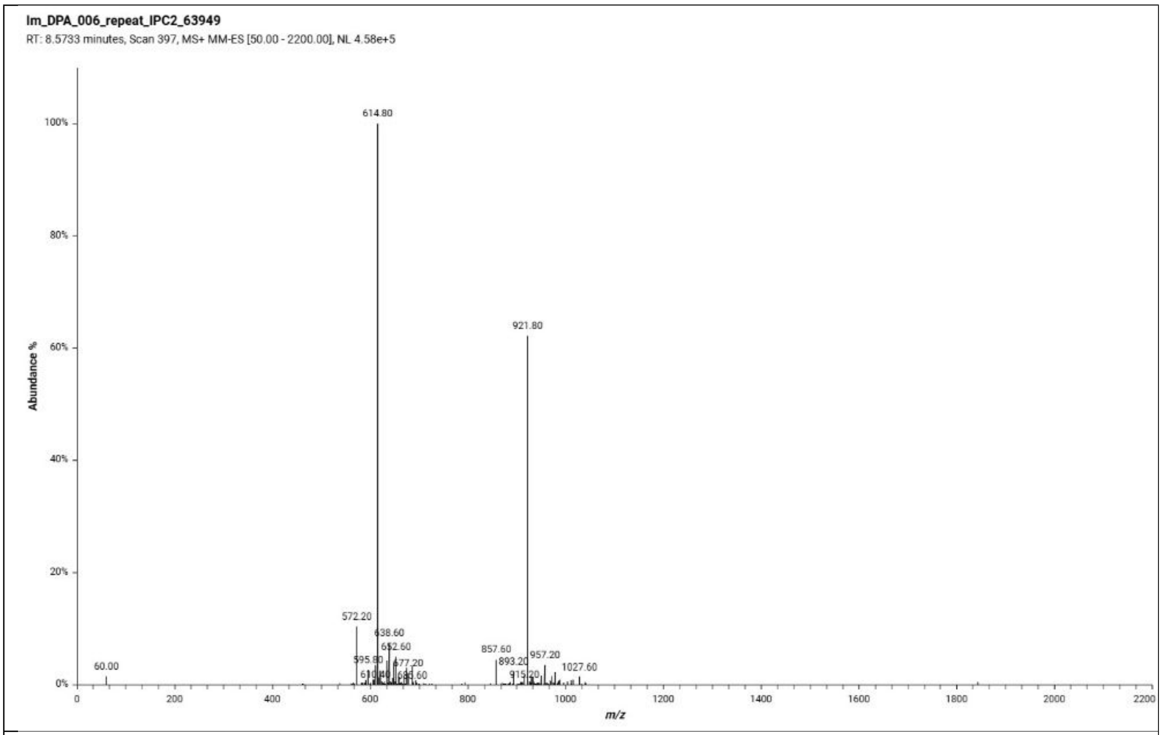
Im_DPA_006_repeat_IPC2_63949
RT: 8.5733 minutes, Scan 397, MS+ MM-ES [50.00 - 2200.00], NL 4.58e+5
Abundance %
m/z
614.80
921.80
572.20
638.60
652.60
595.80
677.20
857.60
893.20
957.20
1027.60
915.20
60.00

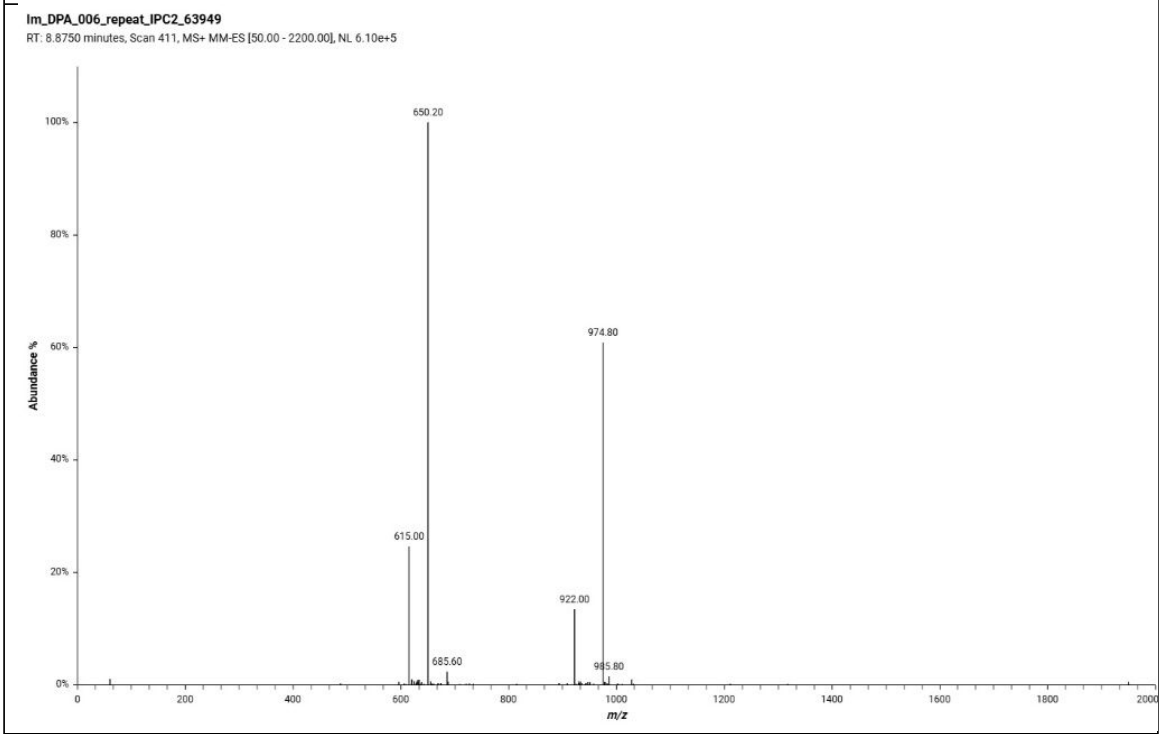
Im_DPA_006_repeat_IPC2_63949
RT: 8.8750 minutes, Scan 411, MS+ MM-ES [50.00 - 2200.00], NL 6.10e+5
Abundance %
m/z
650.20
974.80
615.00
922.00
685.60
985.80

Figure S2: UV traces (at 210nm) and mass spectra of LC-MS analysis of the cleavage product of peptidyl resin **7a** Shown in Figure 3a

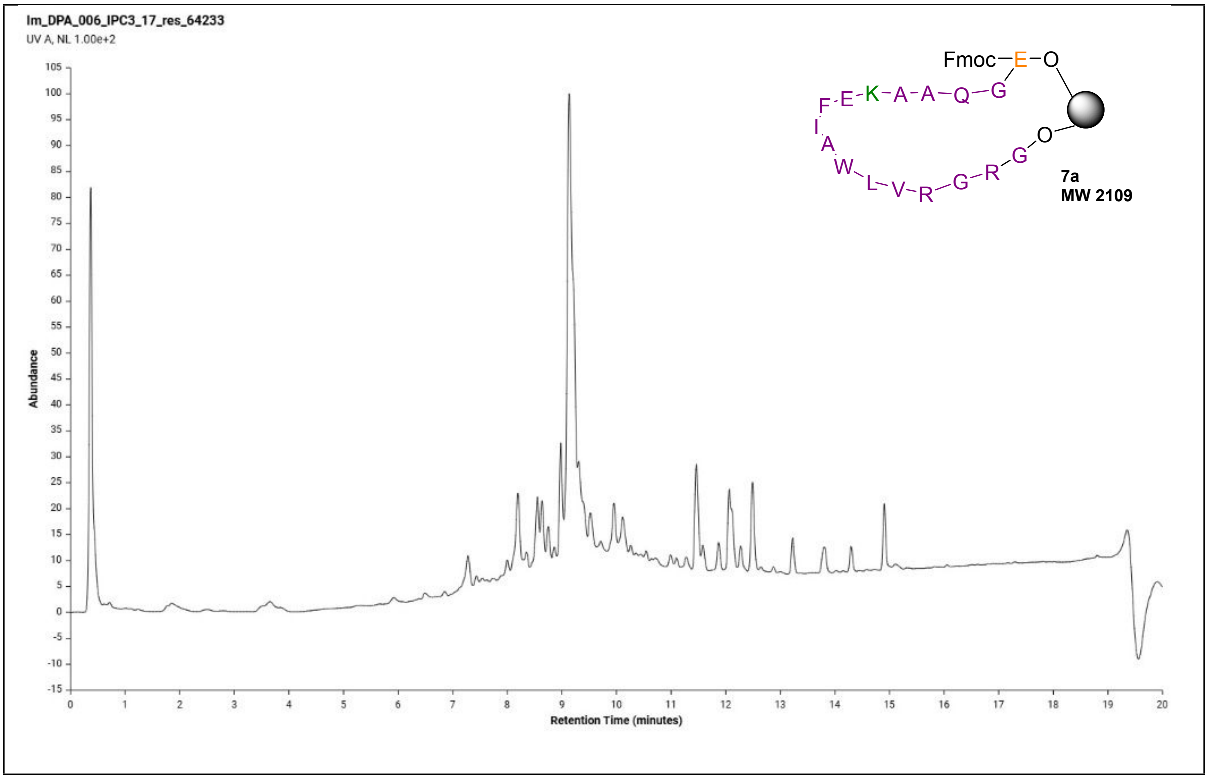

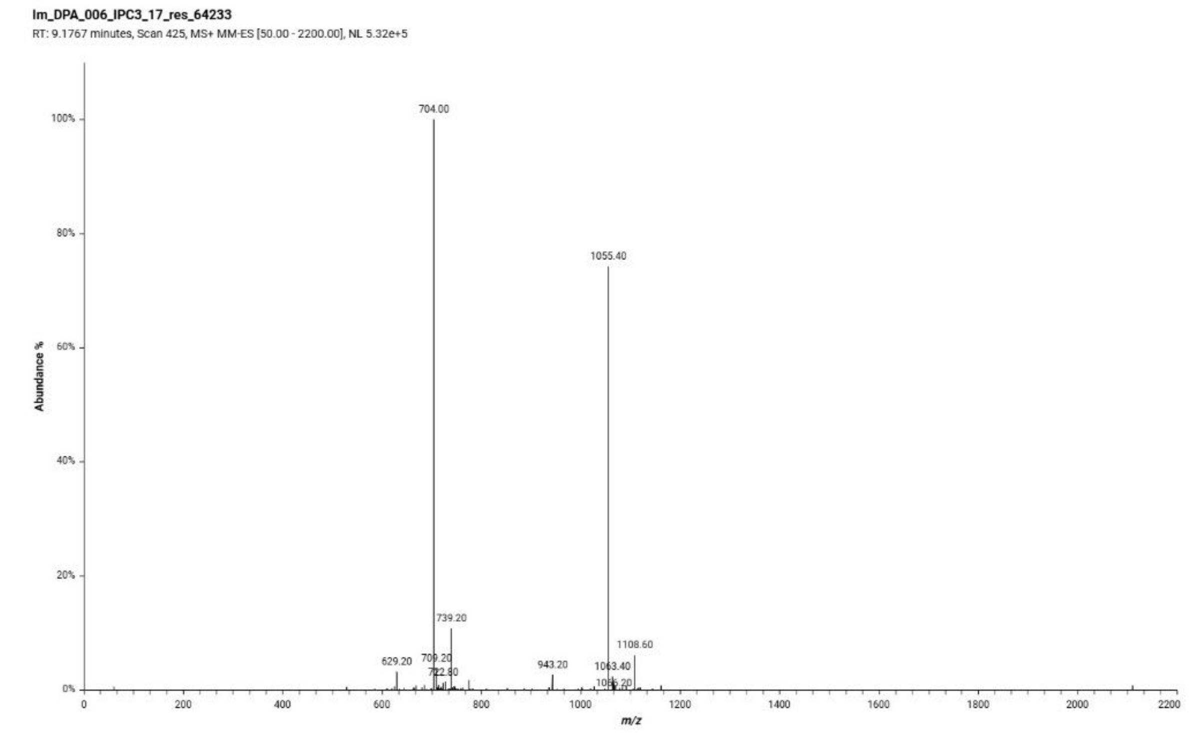

Figure S3: UV traces (at 210nm) and mass spectra of LC-MS analysis of the cleavage product of peptidyl resin **7b** Shown in Figure 3b

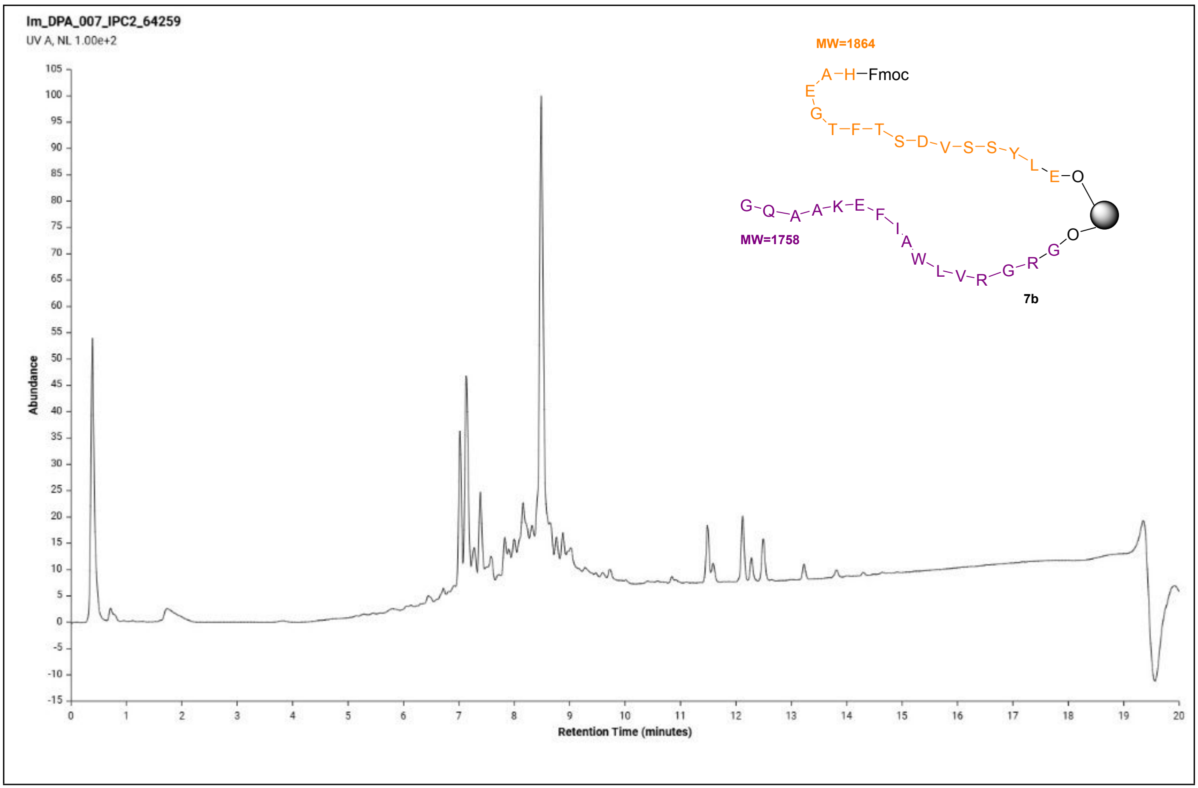

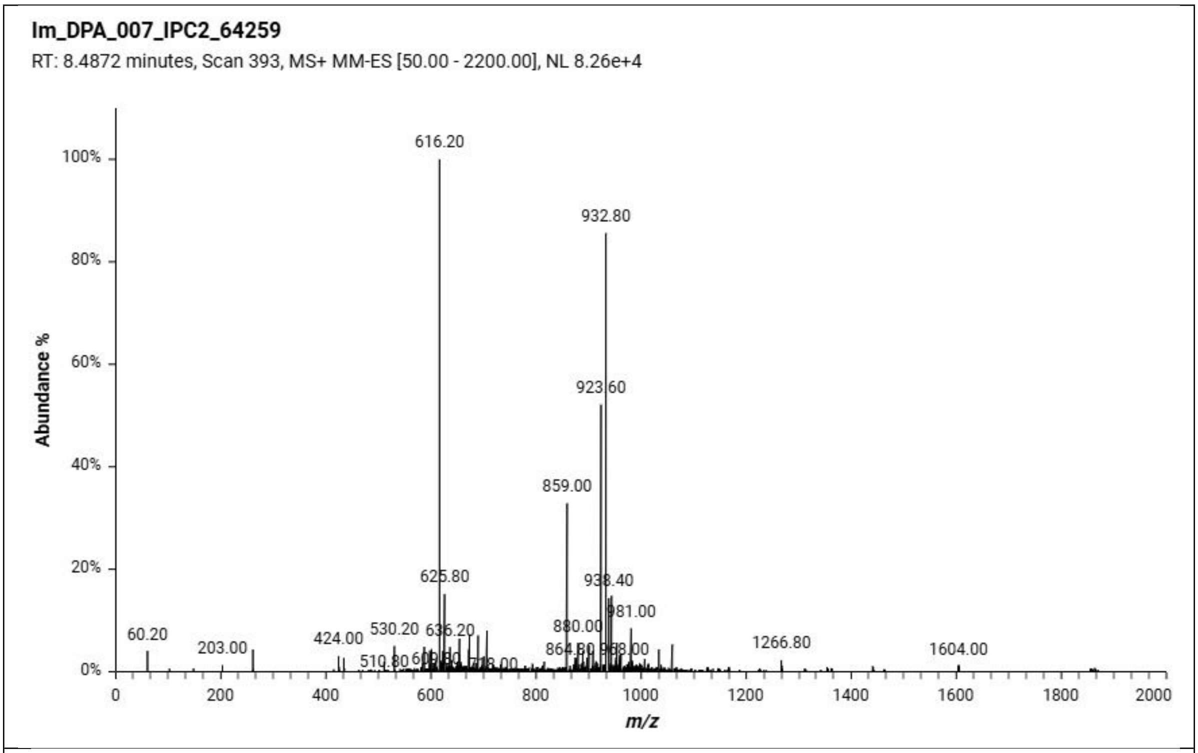
Im_DPA_007_IPC2_64259
RT: 8.4872 minutes, Scan 393, MS+ MM-ES [50.00 - 2200.00], NL 8.26e+4
Abundance %
m/z
616.20
932.80
923.60
859.00
625.80
938.40
981.00
880.00
636.20
530.20
424.00
60.20
203.00
1266.80
1604.00

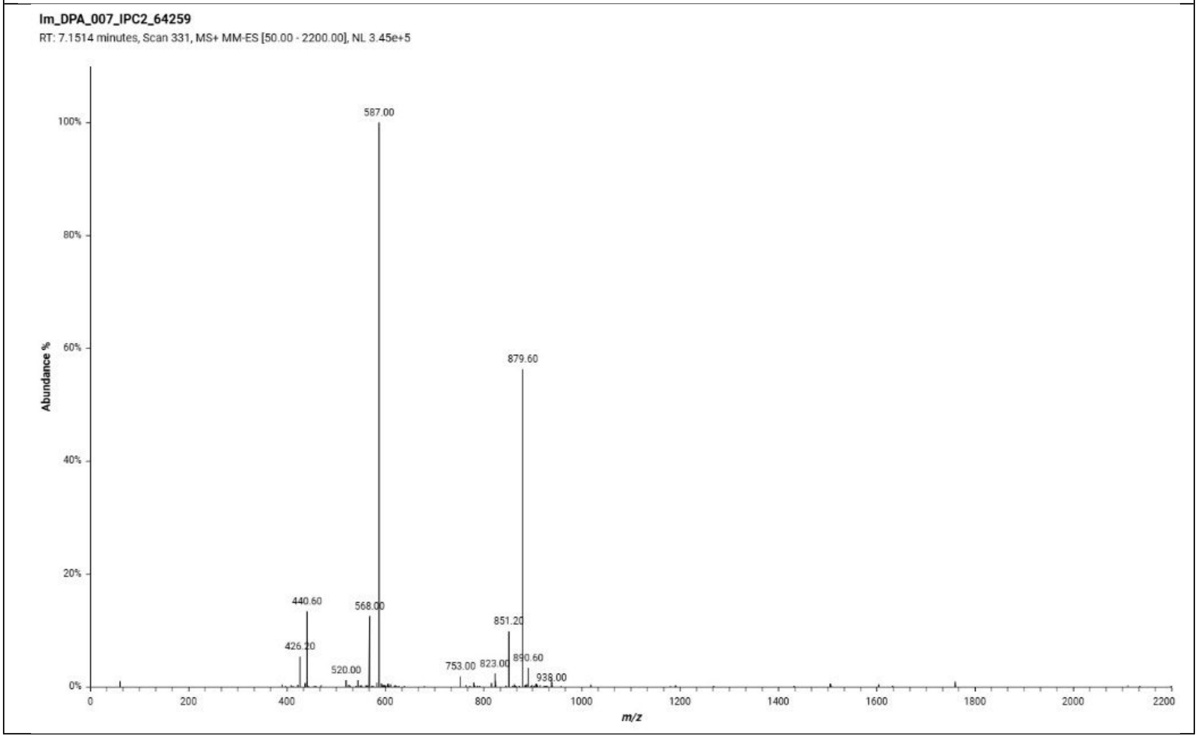
Im_DPA_007_IPC2_64259
RT: 7.1514 minutes, Scan 331, MS+ MM-ES [50.00 - 2200.00], NL 3.45e+5
Abundance %
m/z
587.00
879.60
440.60
568.00
426.20
851.20
520.00
753.00
823.00
890.60
938.00

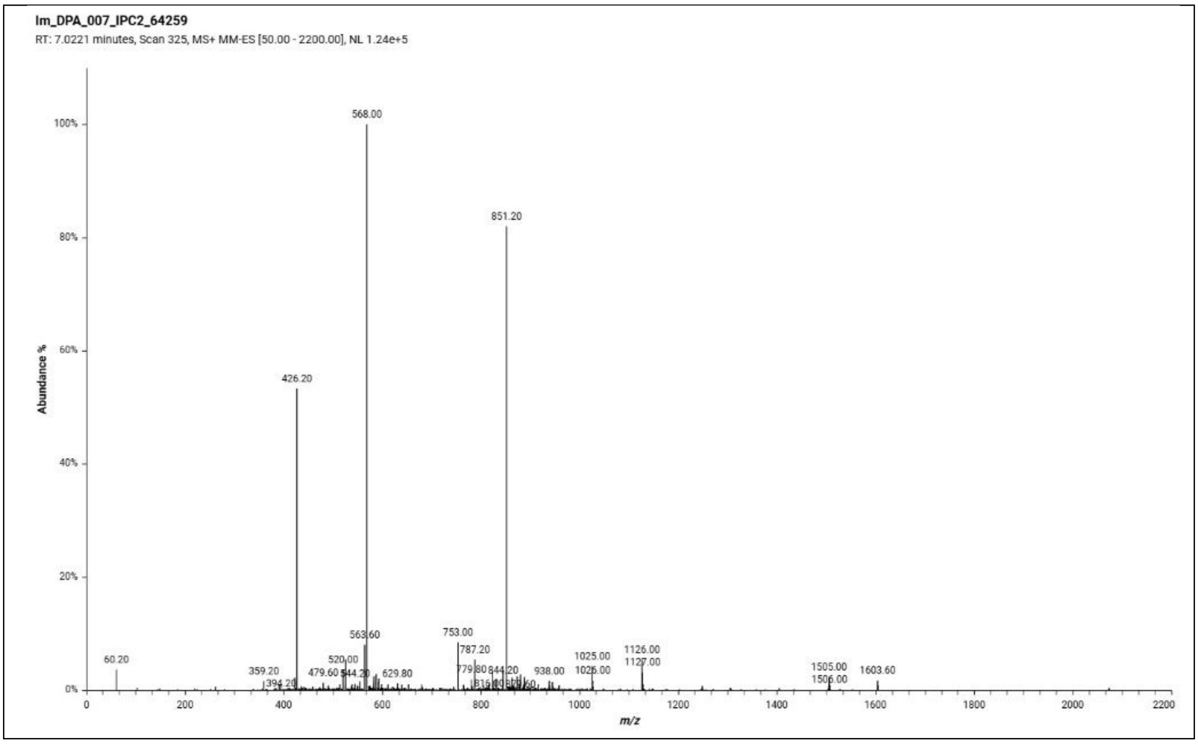

Figure S4: UV traces (at 210nm) and mass spectra of LC-MS analysis of the cleavage product of peptidyl resin **8** Shown in Figure 3b

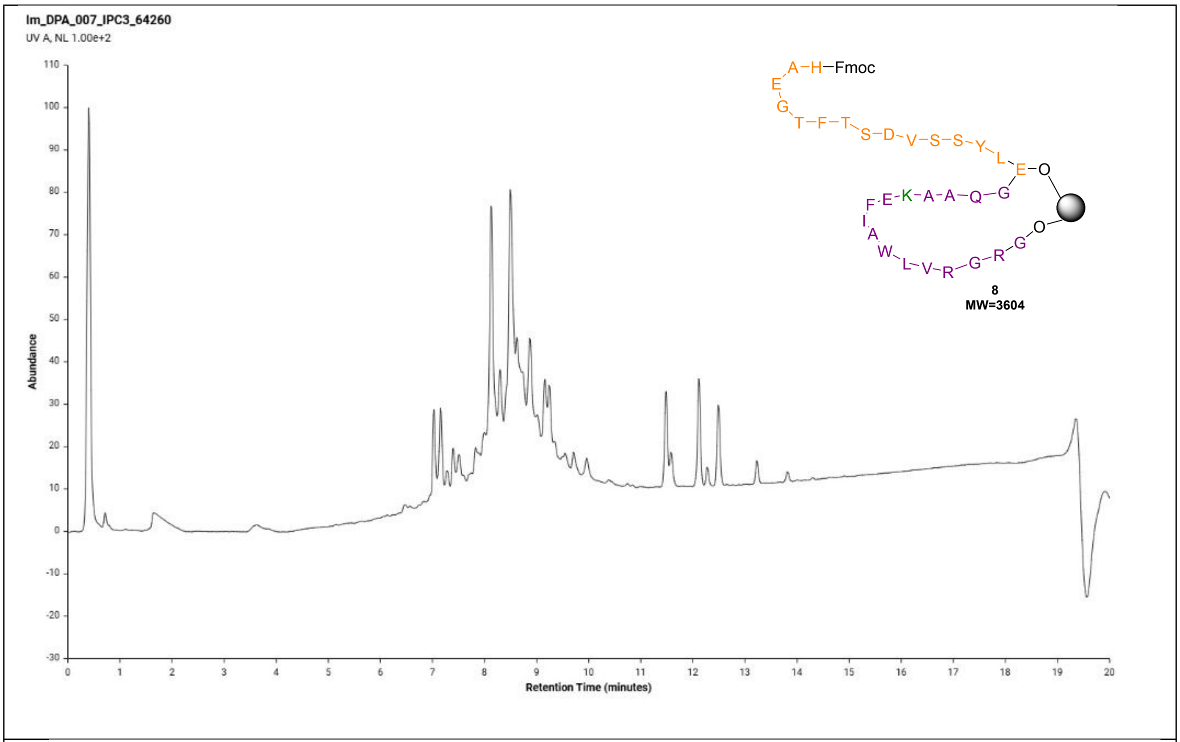

lm_DPA_007_IPC3_64260
UV A, NL 1.00e+2
Abundance
Retention Time (minutes)
Fmoc
8
MW=3604

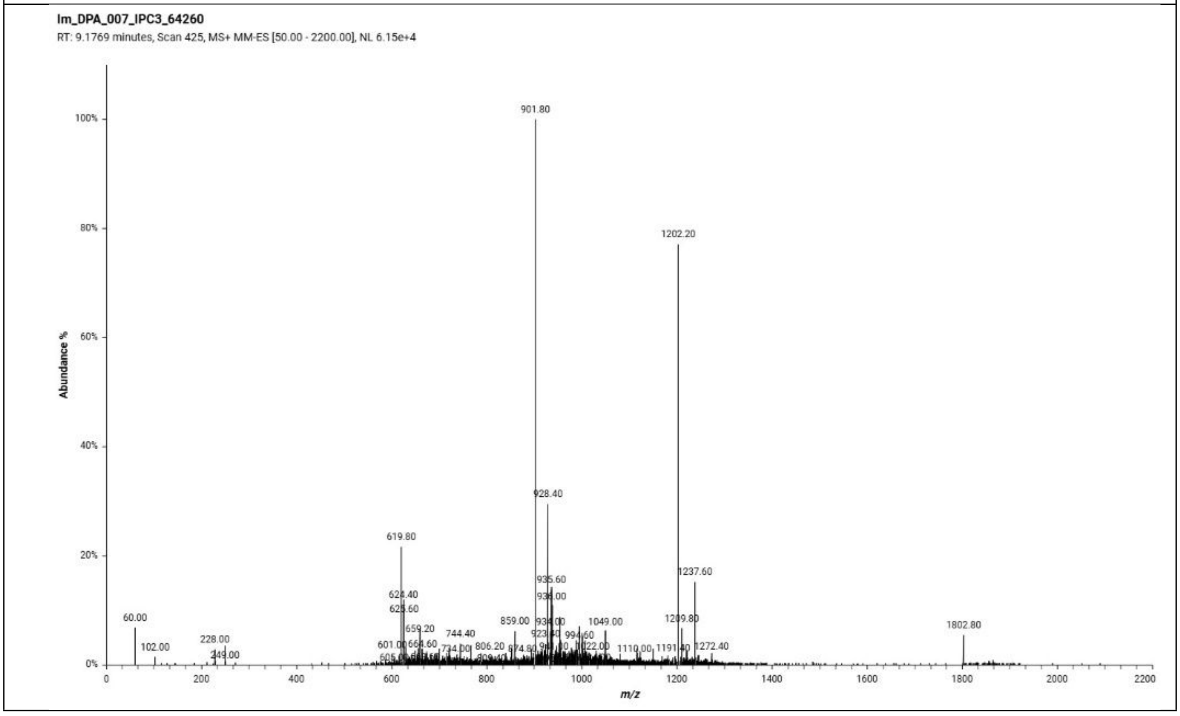

lm_DPA_007_IPC3_64260
RT: 9.1769 minutes, Scan 425, MS+ MM-ES [50.00 - 2200.00], NL 6.15e+4
Abundance %
m/z
901.80
1202.20
928.40
619.80
1237.60
1802.80
60.00
102.00
228.00
1049.00
859.00
744.40

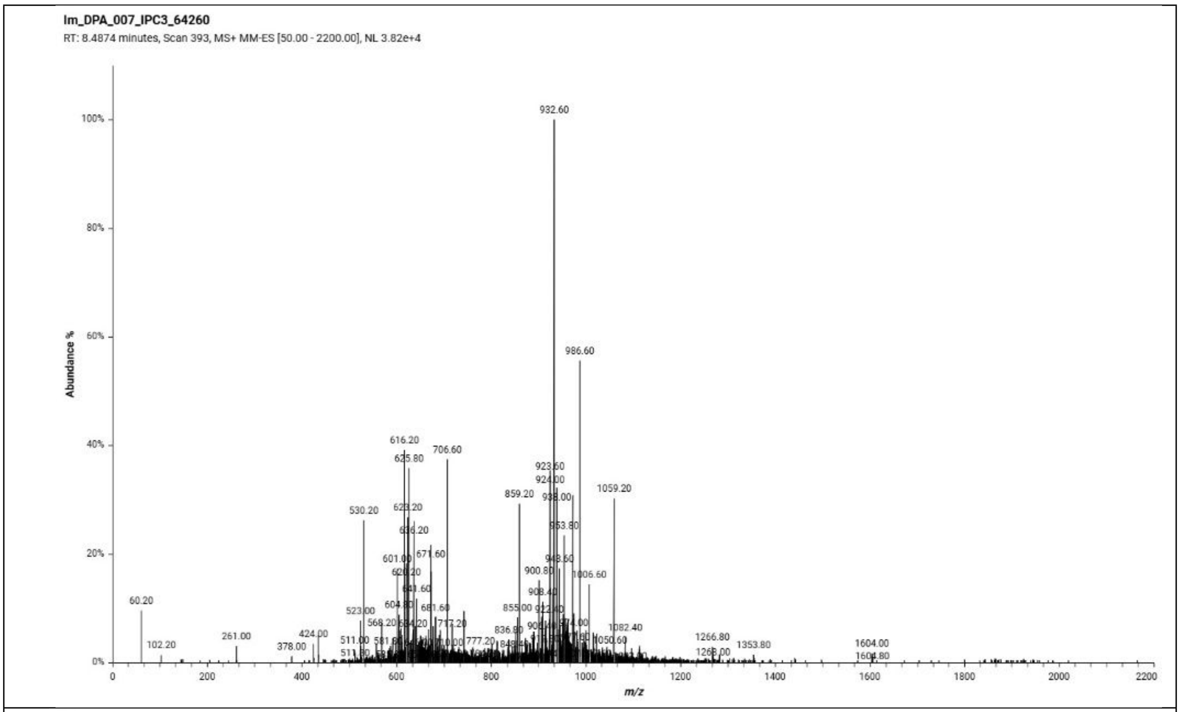
Im_DPA_007_IPC3_64260
RT: 8.4874 minutes, Scan 393, MS+ MM-ES [50.00 - 2200.00], NL 3.82e+4
Abundance %
m/z
932.60
986.60
616.20
625.80
706.60
923.60
924.00
938.00
859.20
1059.20
530.20
953.80
671.60
601.00
900.80
1006.60
60.20
523.00
855.00
1082.40
261.00
424.00
378.00
102.20
1266.80
1353.80
1604.00

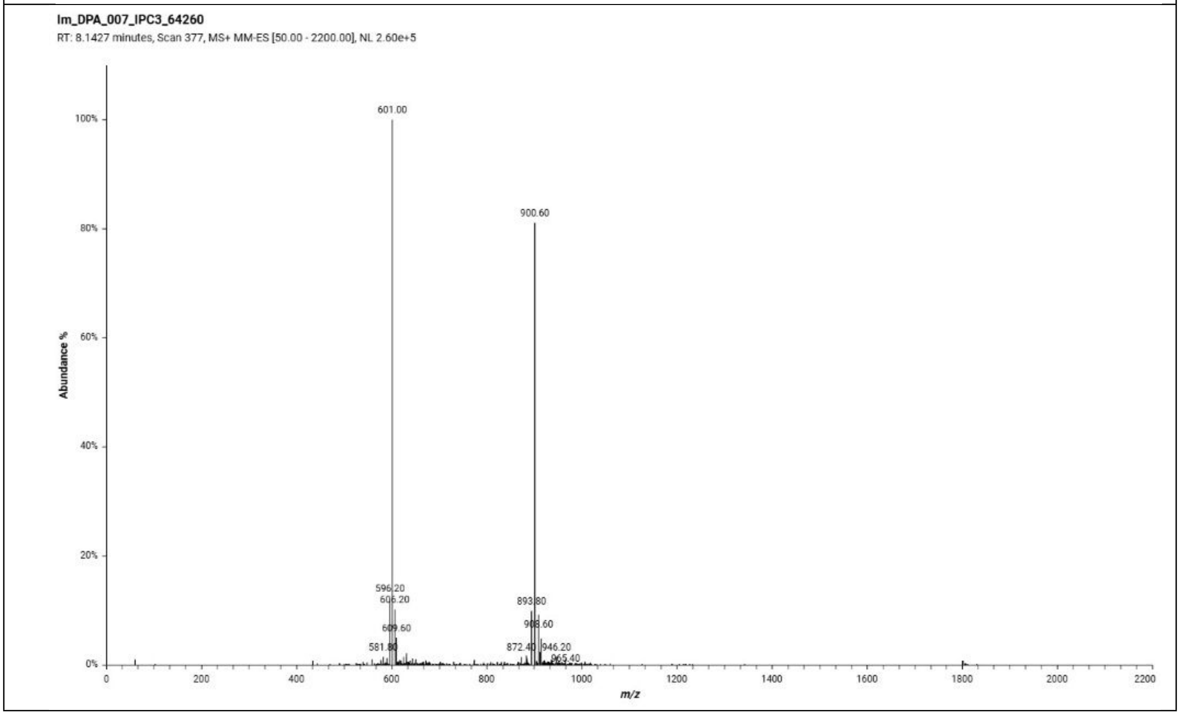
Im_DPA_007_IPC3_64260
RT: 8.1427 minutes, Scan 377, MS+ MM-ES [50.00 - 2200.00], NL 2.60e+5
Abundance %
m/z
601.00
900.60
596.20
606.20
609.60
581.80
893.80
908.60
872.40
946.20
965.40

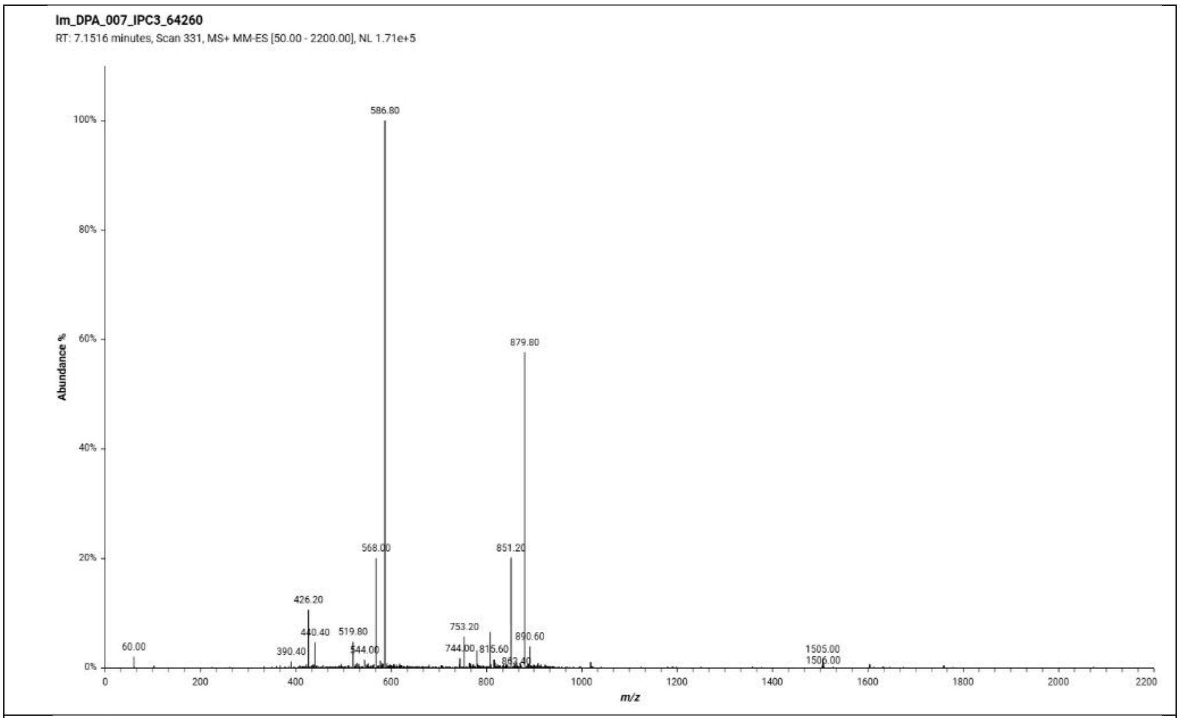
Im_DPA_007_IPC3_64260
RT: 7.1516 minutes, Scan 331, MS+ MM-ES [50.00 - 2200.00], NL 1.71e+5
Abundance %
m/z
586.80
879.80
568.00
851.20
426.20
440.40
519.80
753.20
890.60
60.00
390.40
544.00
744.00
1505.00
1506.00

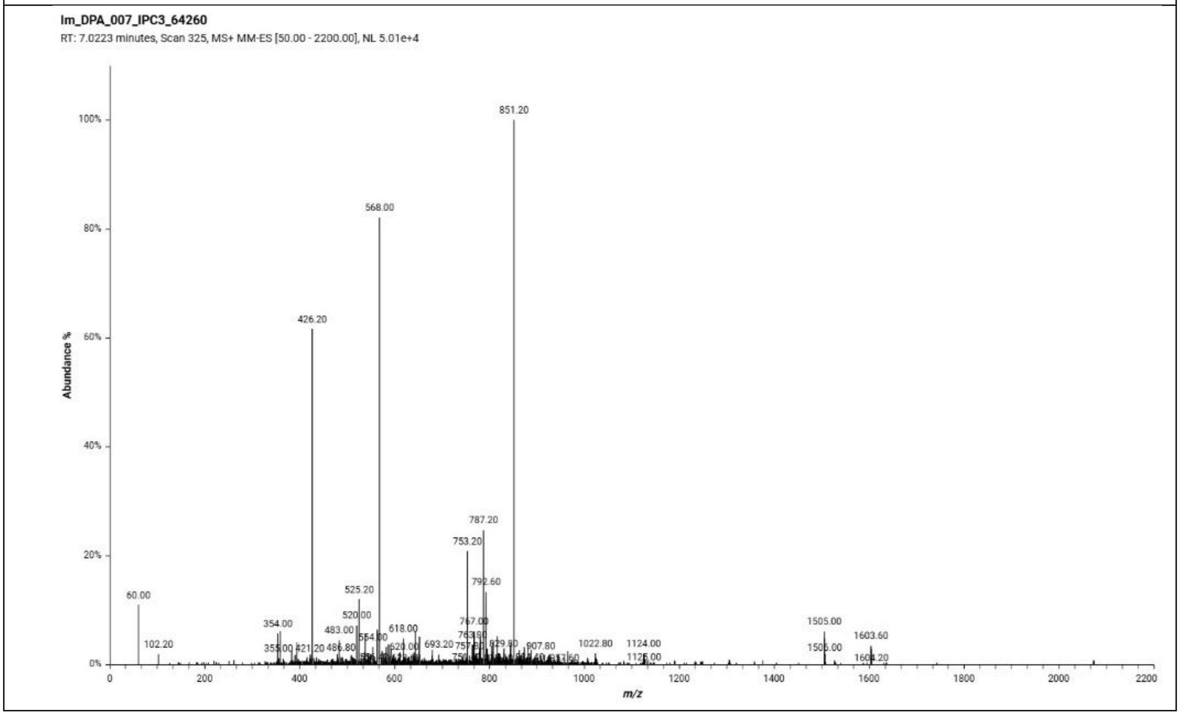
Im_DPA_007_IPC3_64260
RT: 7.0223 minutes, Scan 325, MS+ MM-ES [50.00 - 2200.00], NL 5.01e+4
Abundance %
m/z
851.20
568.00
426.20
787.20
753.20
792.60
525.20
60.00
354.00
520.00
483.00
618.00
767.00
102.20
693.20
907.80
1022.80
1124.00
1505.00
1603.60

Figure S5: UV traces (at 210nm) and mass spectra of LC-MS analysis of the crude product **8** Shown in Figure **4a**

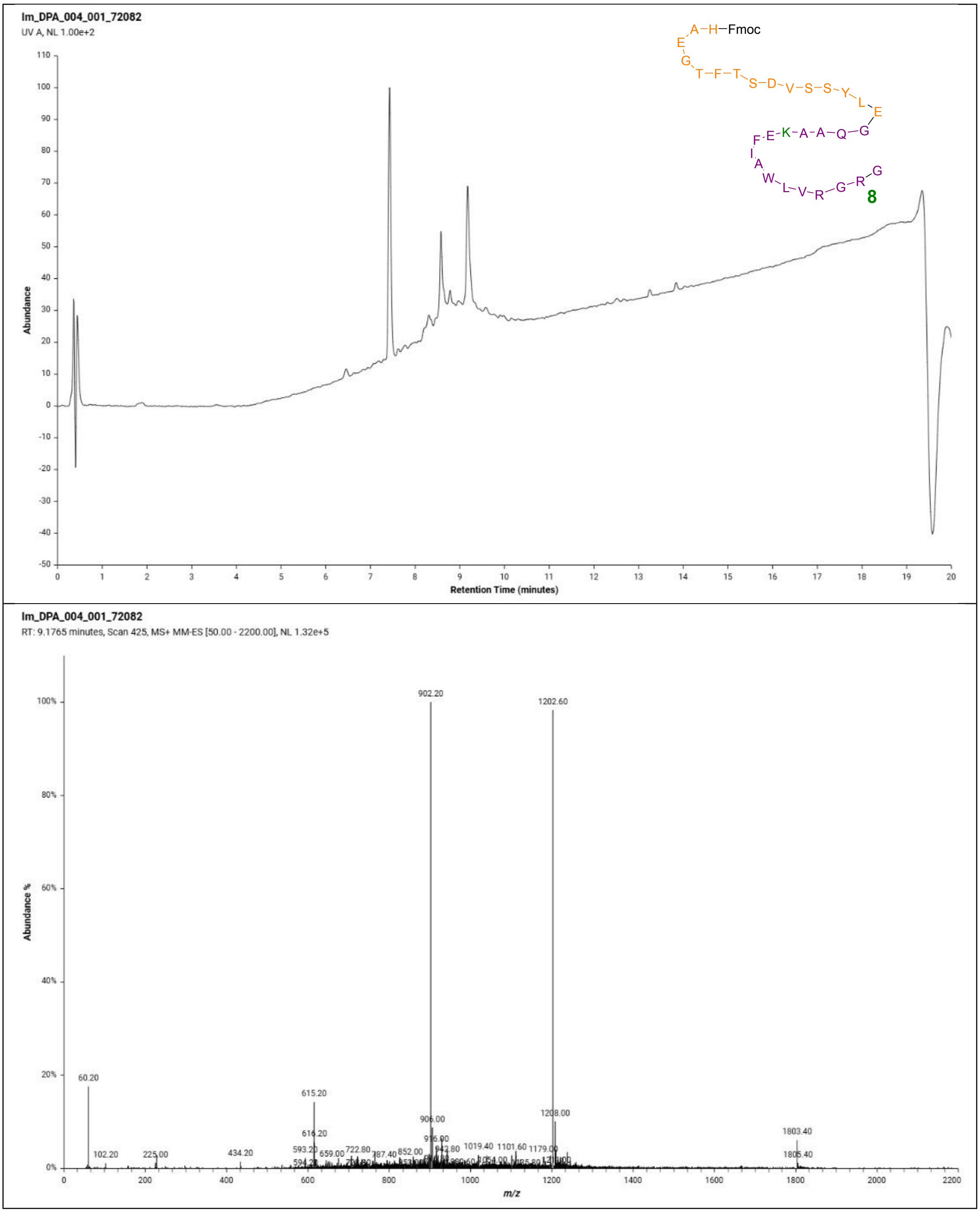

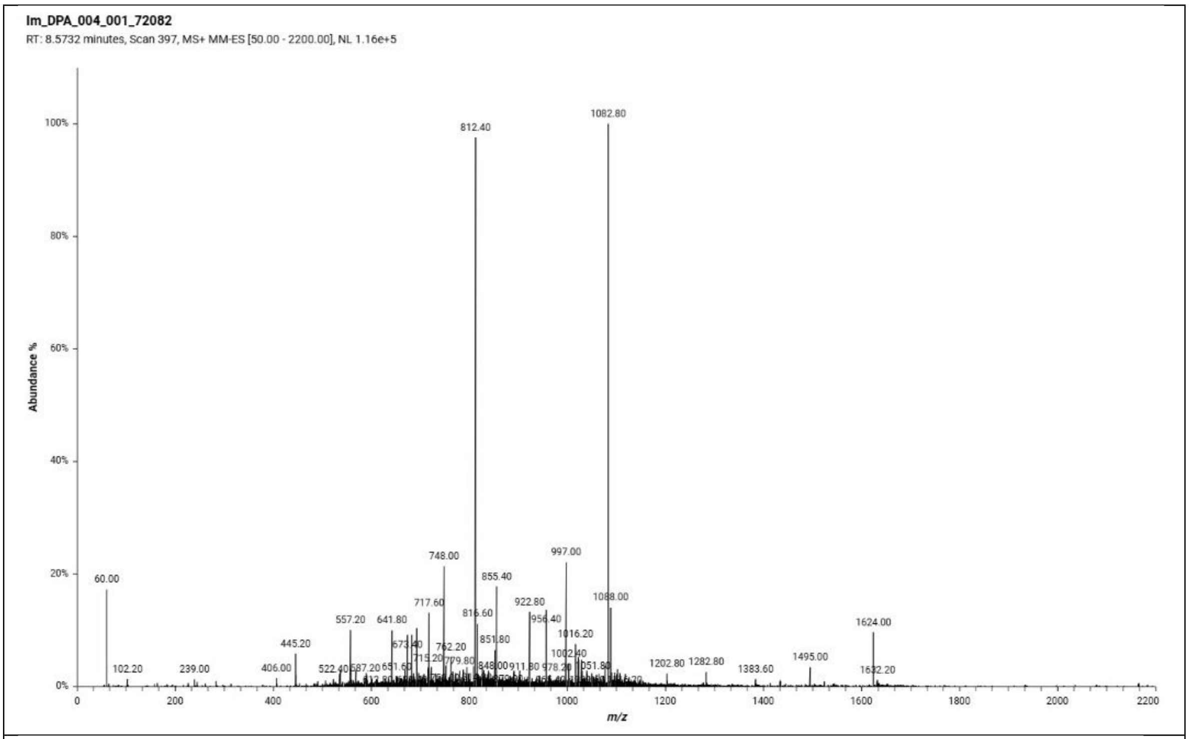
Im_DPA_004_001_72082
RT: 8.5732 minutes, Scan 397, MS+ MM-ES [50.00 - 2200.00], NL 1.16e+5
Abundance %
m/z
812.40
1082.80
748.00
997.00
855.40
60.00
1088.00
717.60
922.80
557.20
641.80
1624.00
445.20
1016.20
1495.00
1202.80
1282.80
1383.60
102.20
239.00
406.00
522.40

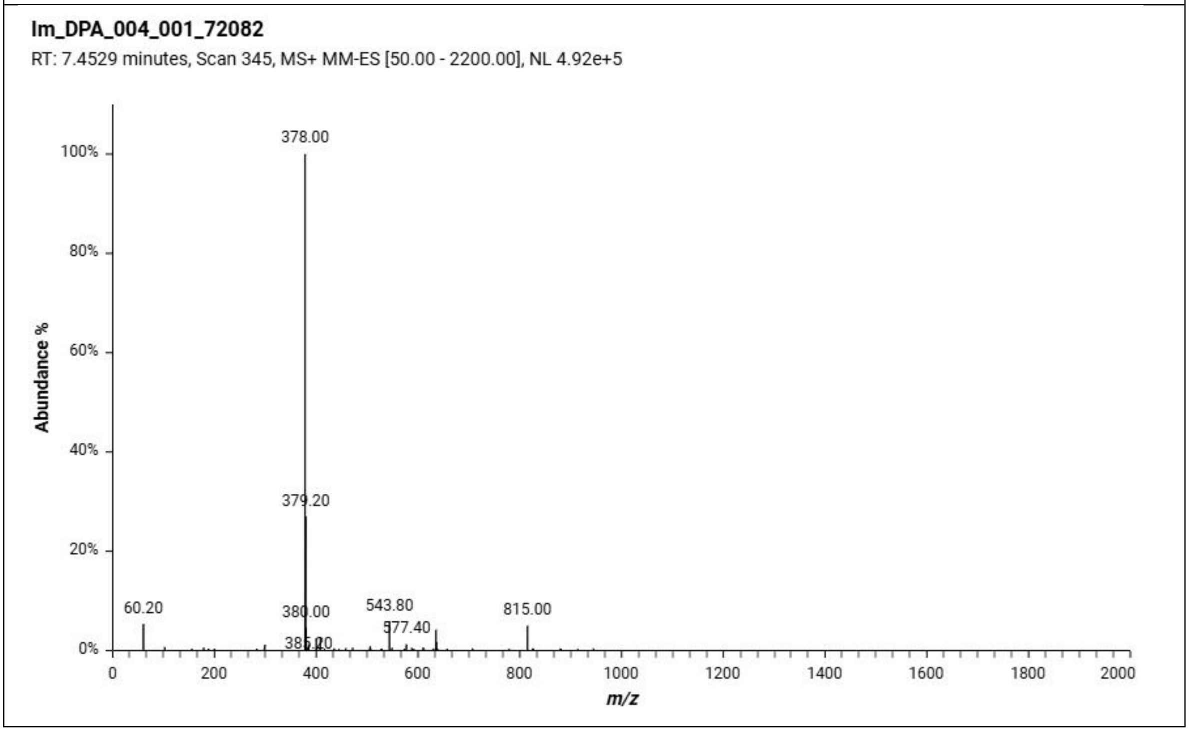
Im_DPA_004_001_72082
RT: 7.4529 minutes, Scan 345, MS+ MM-ES [50.00 - 2200.00], NL 4.92e+5
Abundance %
m/z
378.00
379.20
60.20
380.00
543.80
577.40
815.00

Figure S6: UV traces (at 210nm) and mass spectra of LC-MS analysis of the crude product **8** Shown in Figure **4c**

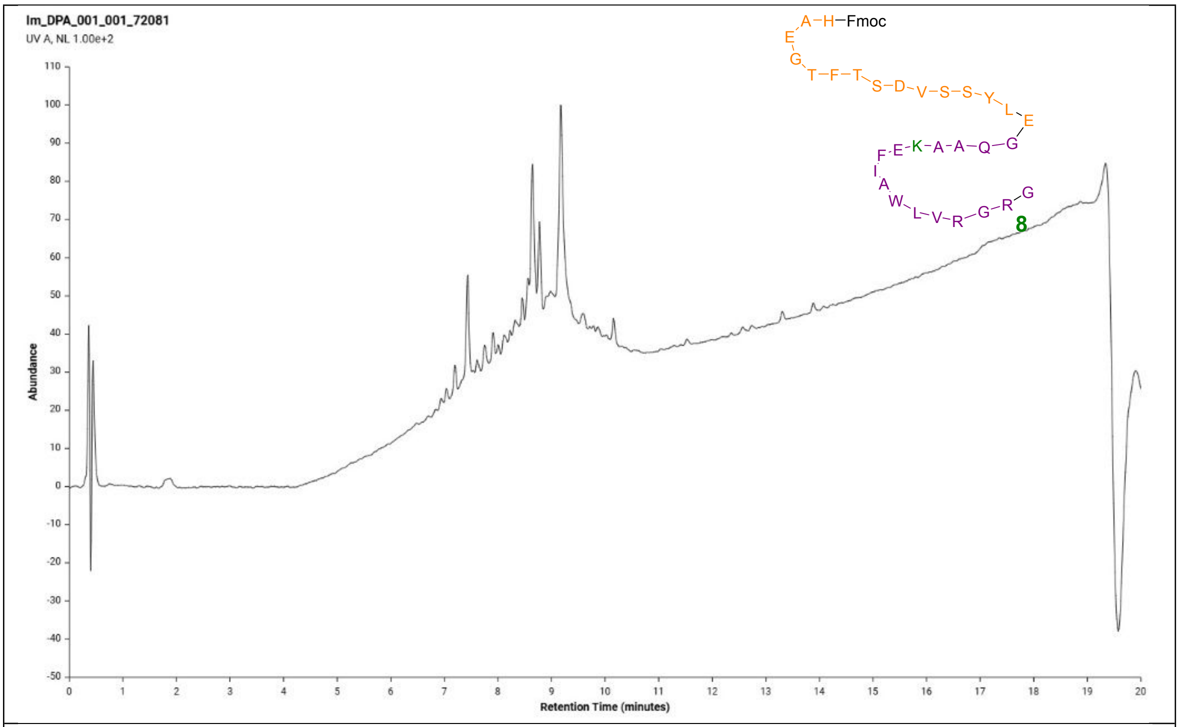

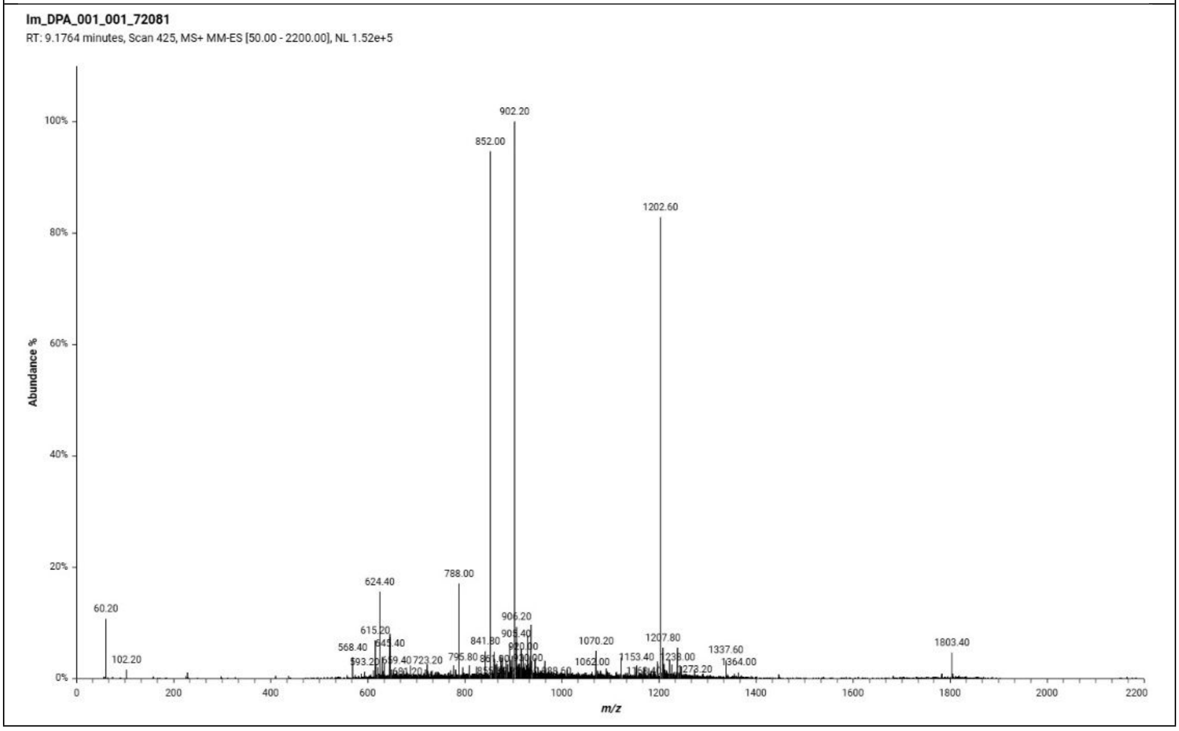

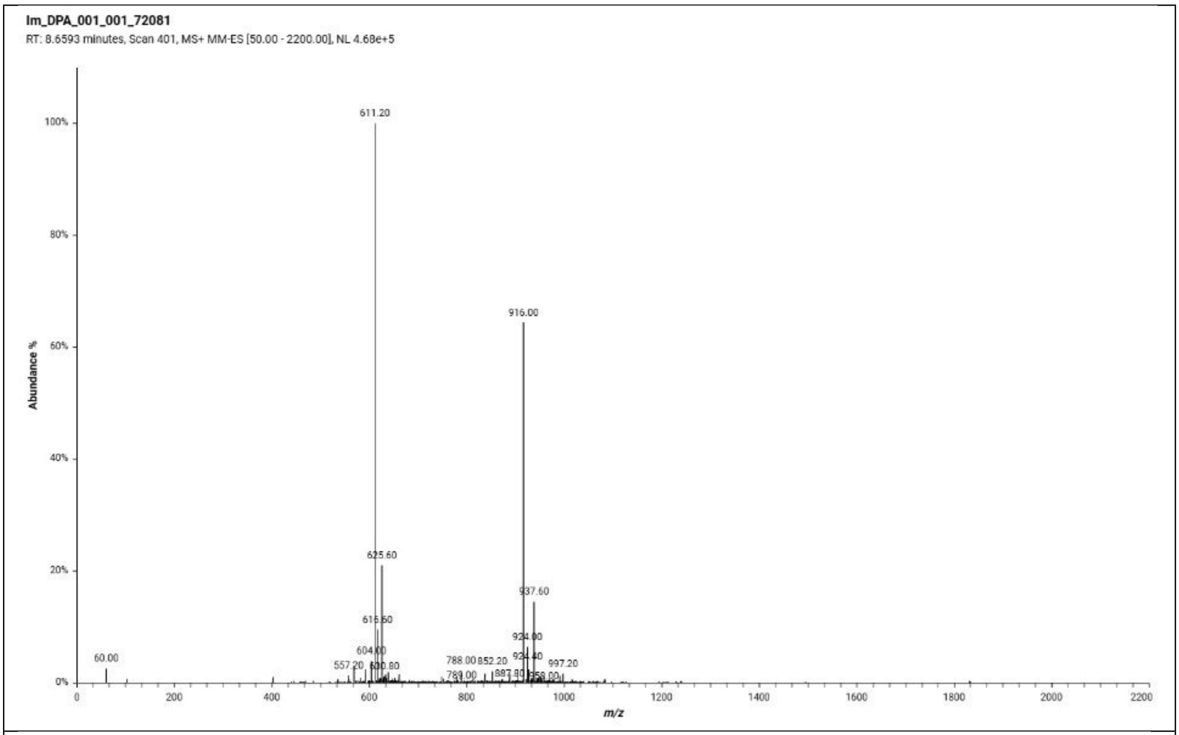
Im_DPA_001_001_72081
RT: 8.6593 minutes, Scan 401, MS+ MM-ES [50.00 - 2200.00], NL 4.68e+5
Abundance %
m/z
611.20
916.00
625.60
937.60
615.60
924.00
604.00
60.00
557.20
788.00
852.20
997.20

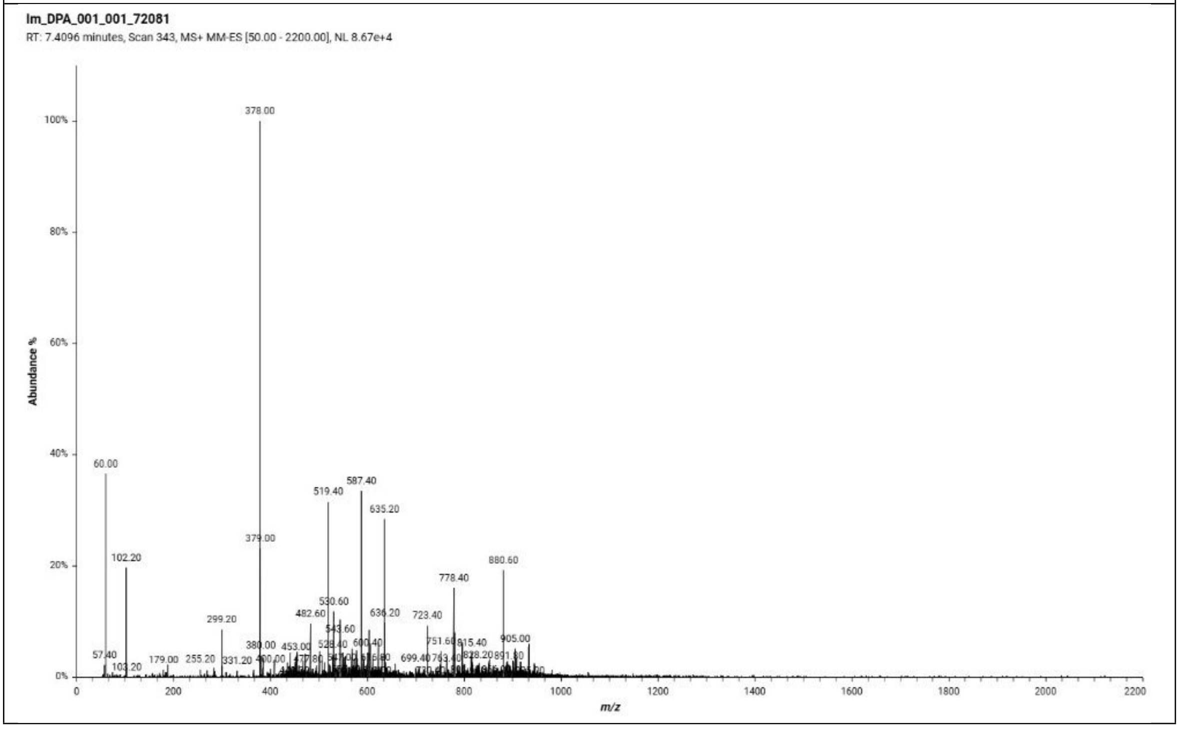
Im_DPA_001_001_72081
RT: 7.4096 minutes, Scan 343, MS+ MM-ES [50.00 - 2200.00], NL 8.67e+4
Abundance %
m/z
378.00
60.00
587.40
519.40
635.20
379.00
102.20
880.60
778.40
482.60
530.60
636.20
723.40
299.20
543.60
751.60
905.00
380.00
453.00
57.40
179.00
255.20
331.20
103.20
699.40
828.20

Figure S7: UV traces (at 210nm) and mass spectra of LC-MS analysis of the crude product **8** Shown in Figure **4e**

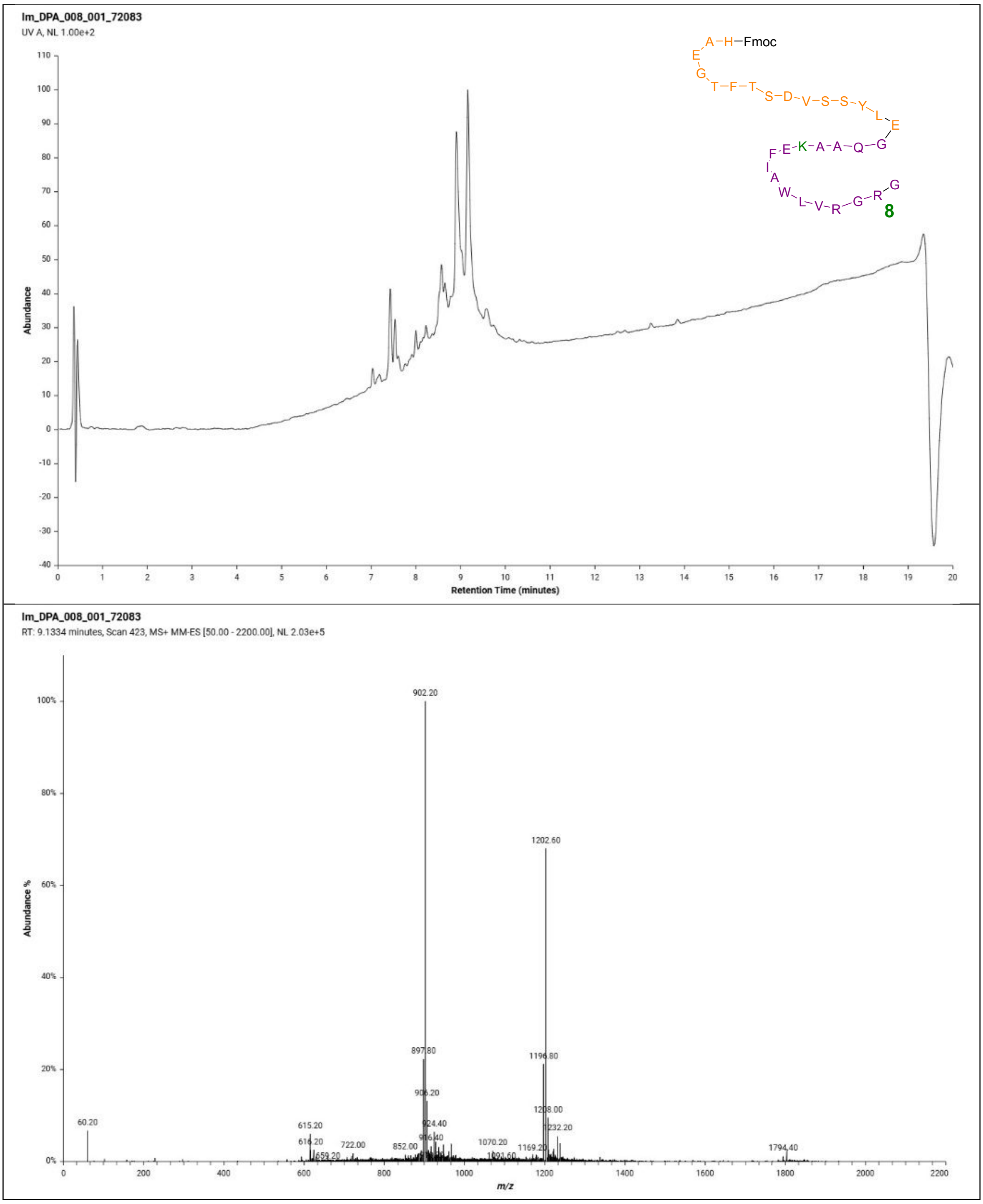

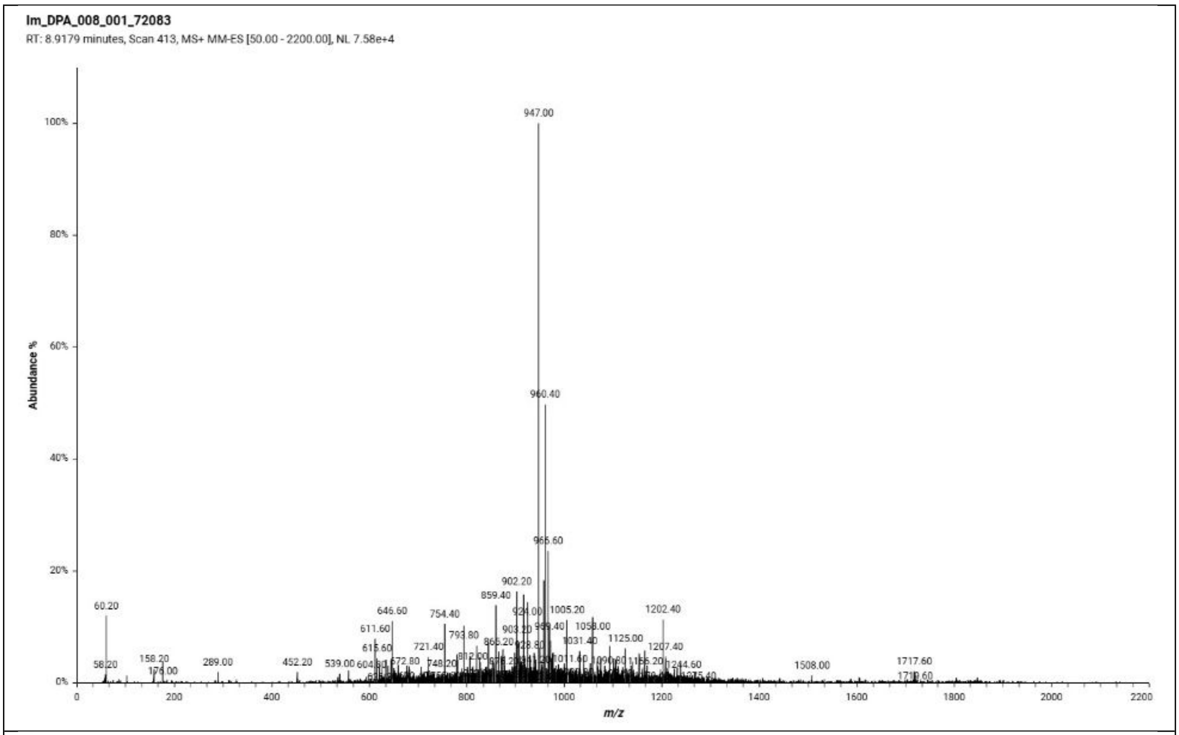
Im_DPA_008_001_72083
RT: 8.9179 minutes, Scan 413, MS+ MM-ES [50.00 - 2200.00], NL 7.58e+4
Abundance %
m/z

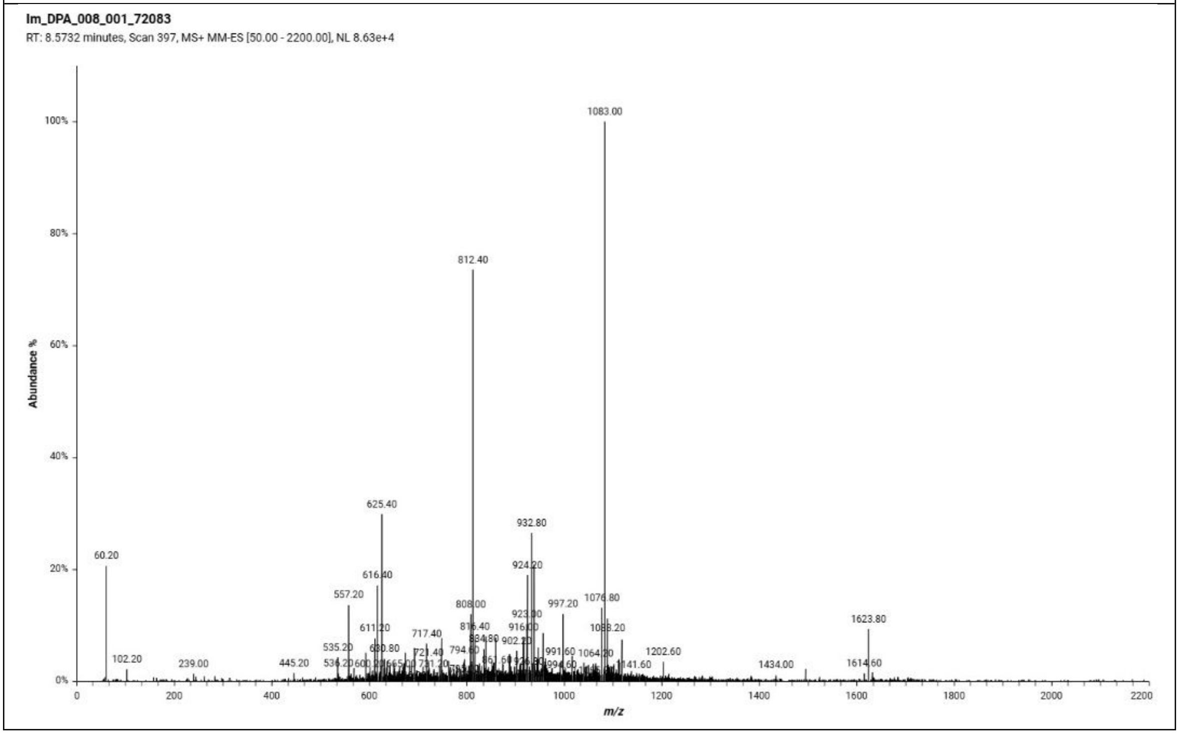
Im_DPA_008_001_72083
RT: 8.5732 minutes, Scan 397, MS+ MM-ES [50.00 - 2200.00], NL 8.63e+4
Abundance %
m/z

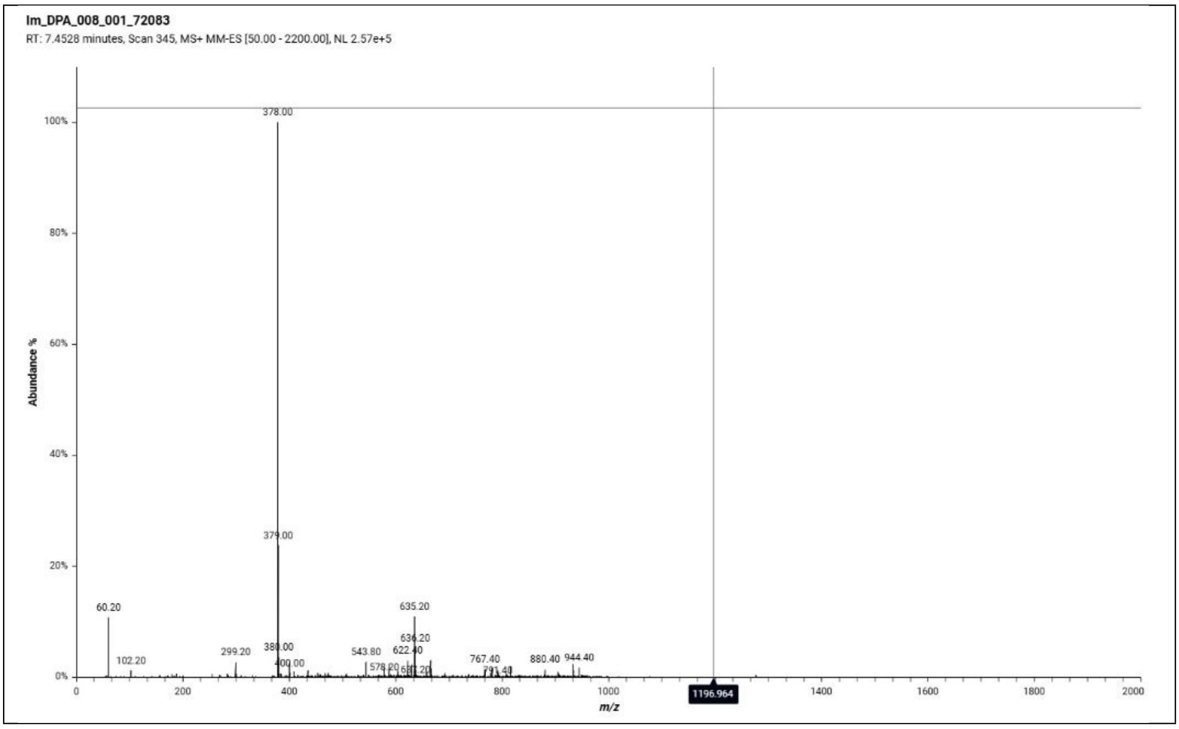
Im_DPA_008_001_72083
RT: 7.4528 minutes, Scan 345, MS+ MM-ES [50.00 - 2200.00], NL 2.57e+5
Abundance %
m/z
378.00
379.00
60.20
102.20
299.20
380.00
400.00
543.80
578.20
622.40
635.20
636.20
767.40
880.40
944.40
1196.964

Figure S8: UV traces (at 210nm) and mass spectra of LC-MS analysis of the crude product **7a** Shown in Figure **4b**

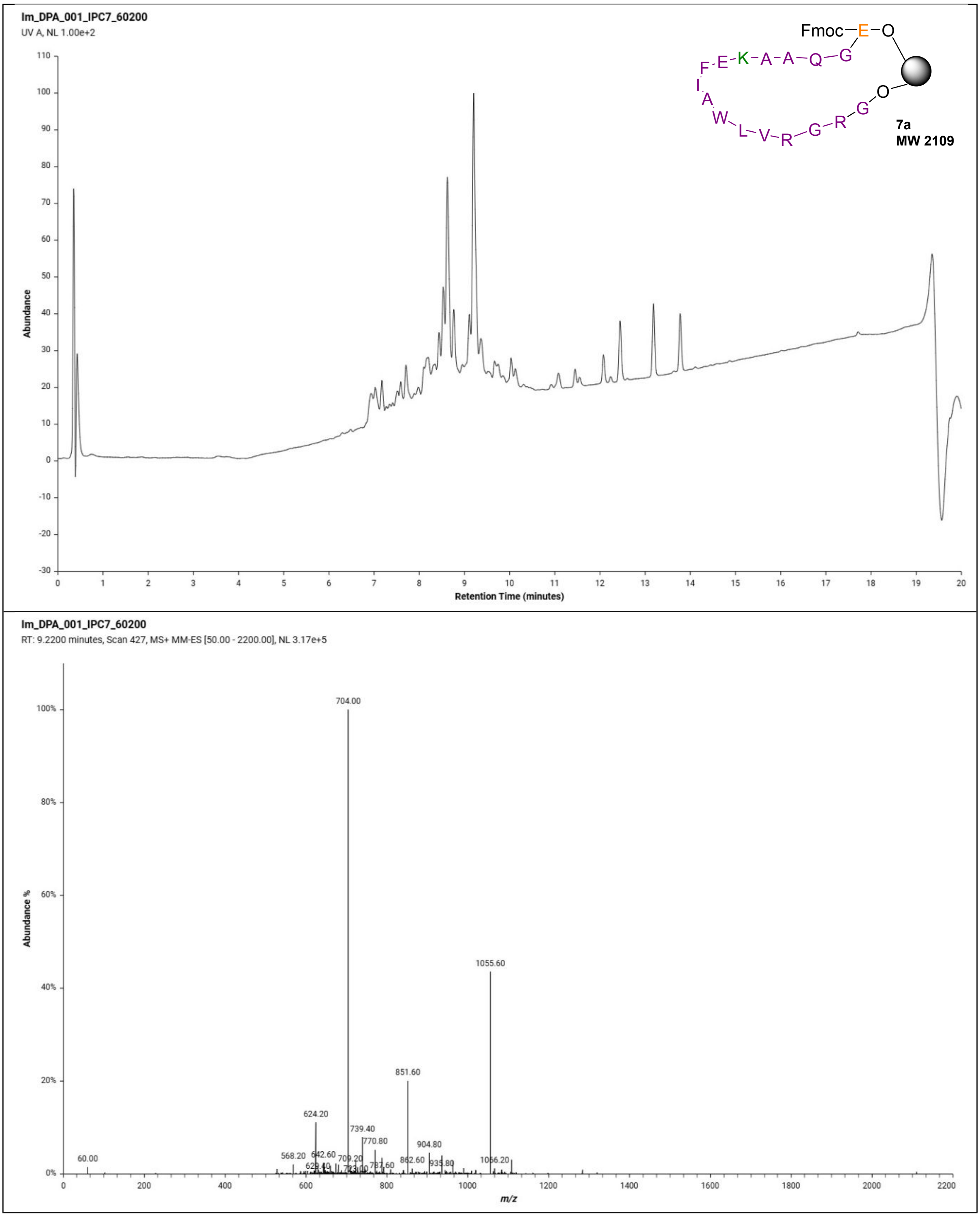

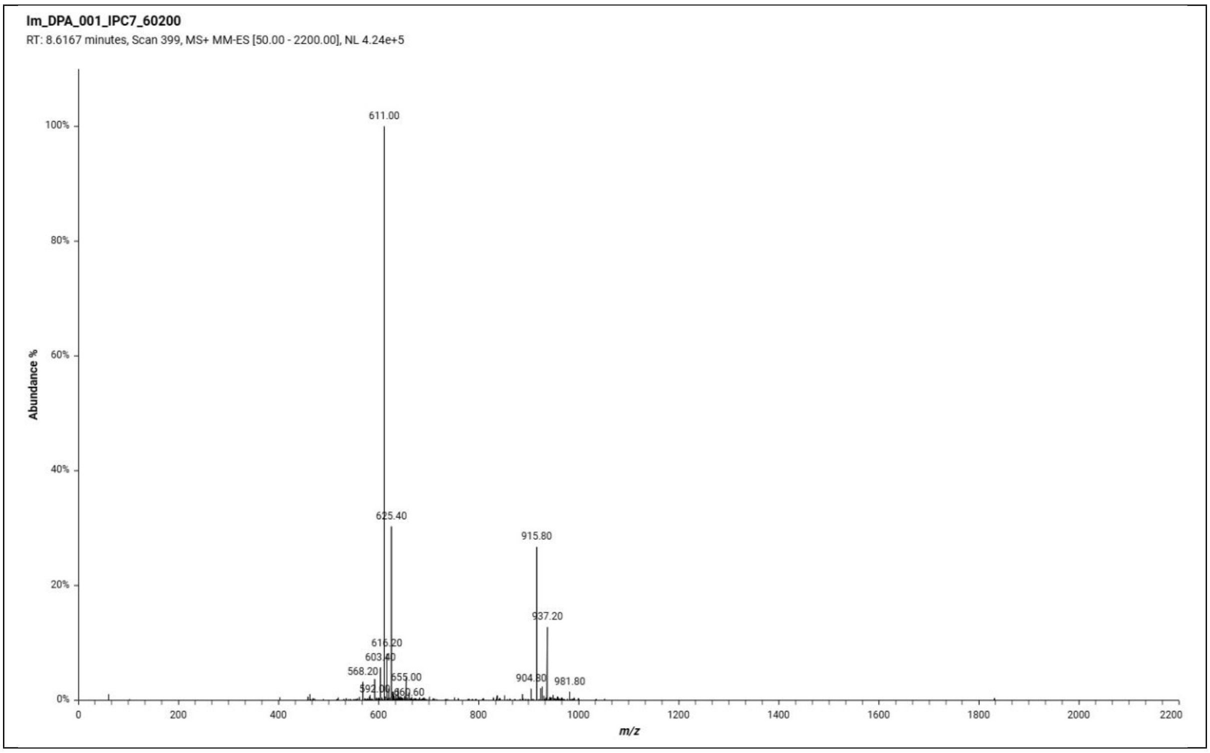
Im_DPA_001_IPC7_60200
RT: 8.6167 minutes, Scan 399, MS+ MM-ES [50.00 - 2200.00], NL 4.24e+5
Abundance %
m/z
611.00
625.40
915.80
937.20
616.20
603.40
568.20
655.00
904.80
981.80

Figure S9: UV traces (at 210nm) and mass spectra of LC-MS analysis of the crude product **7a** Shown in Figure **4d**

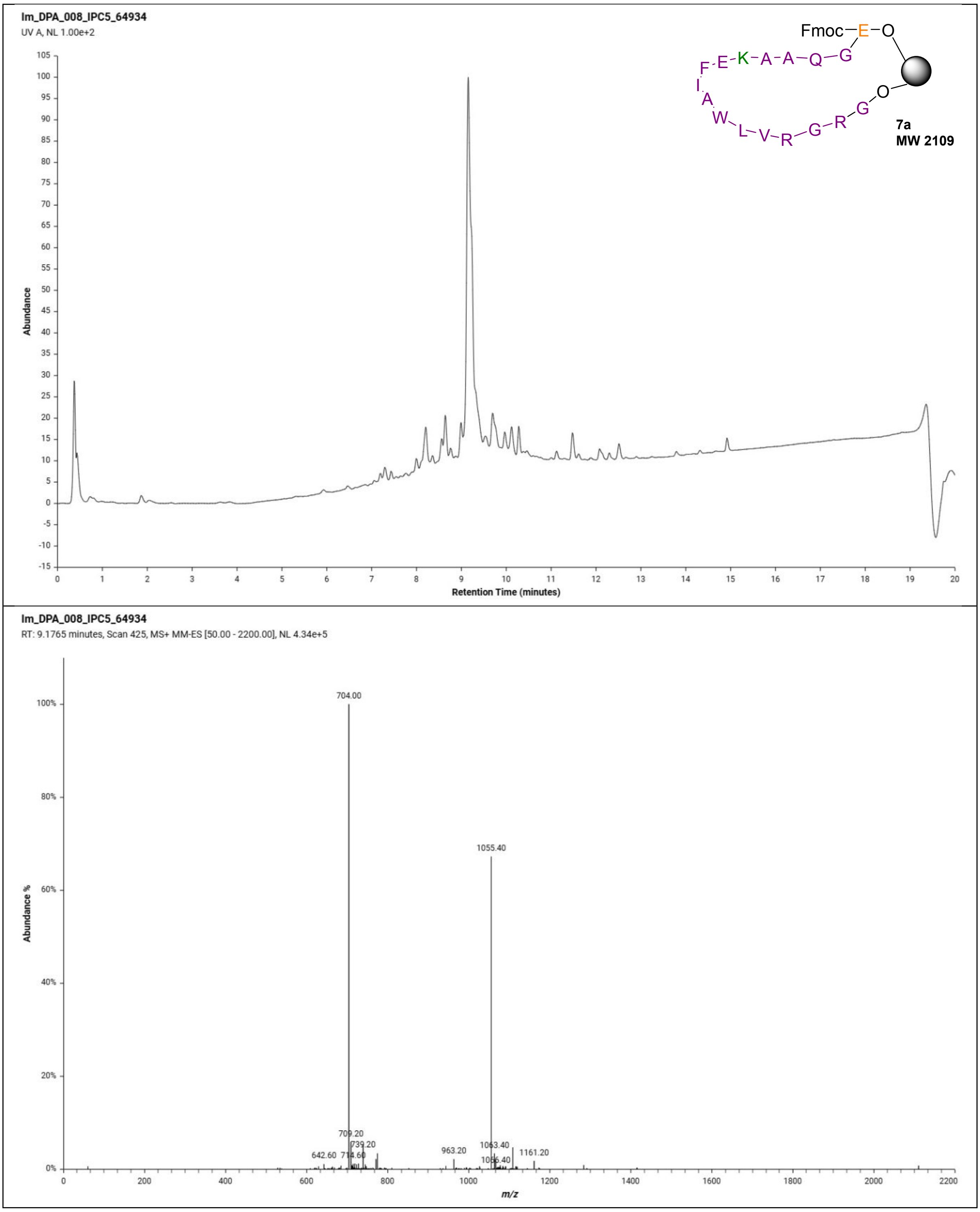

Figure S10: UV traces (at 210nm) and mass spectra of LC-MS analysis of the mini-cleavage product of peptidyl resin **14**, shown in figure 5a

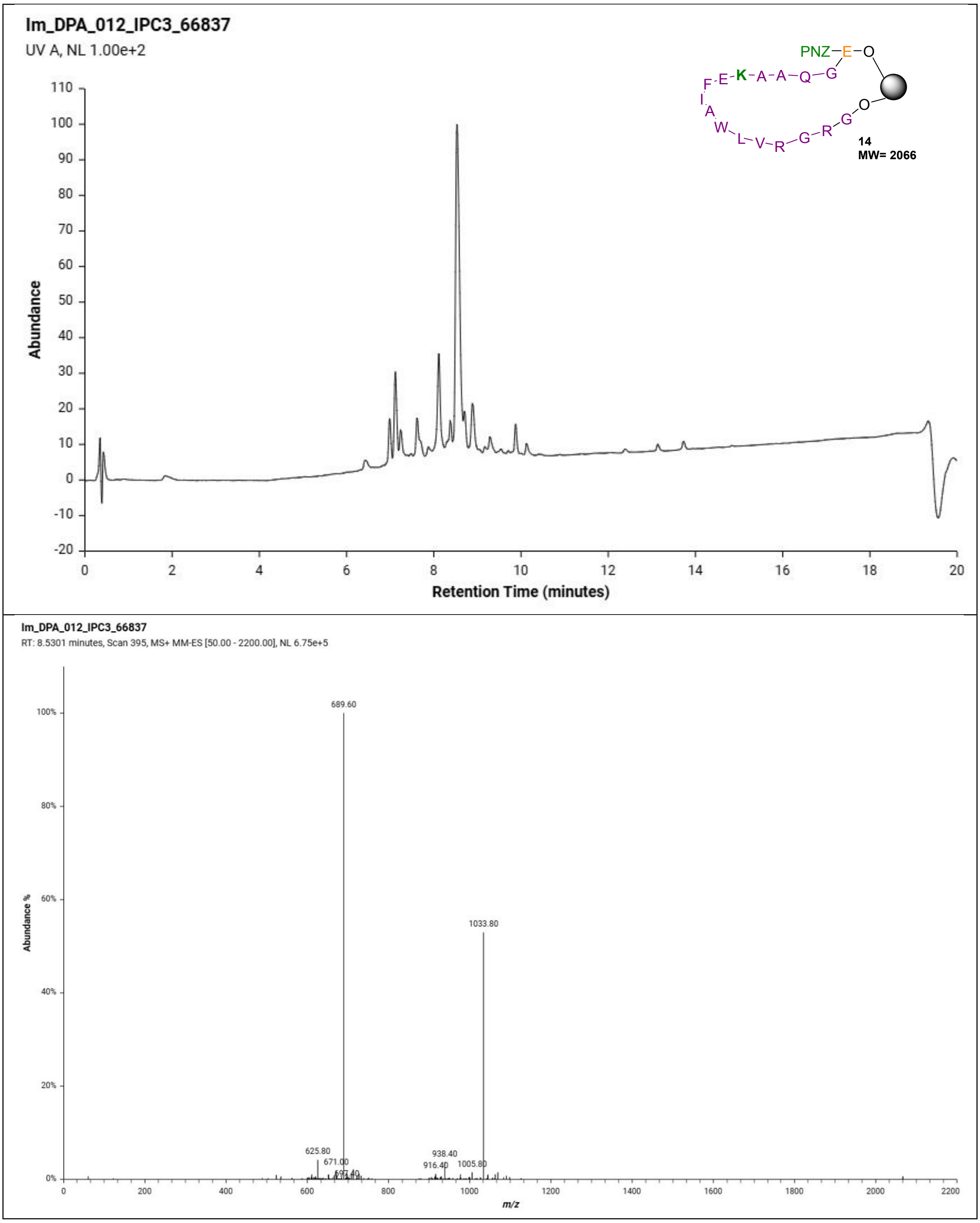

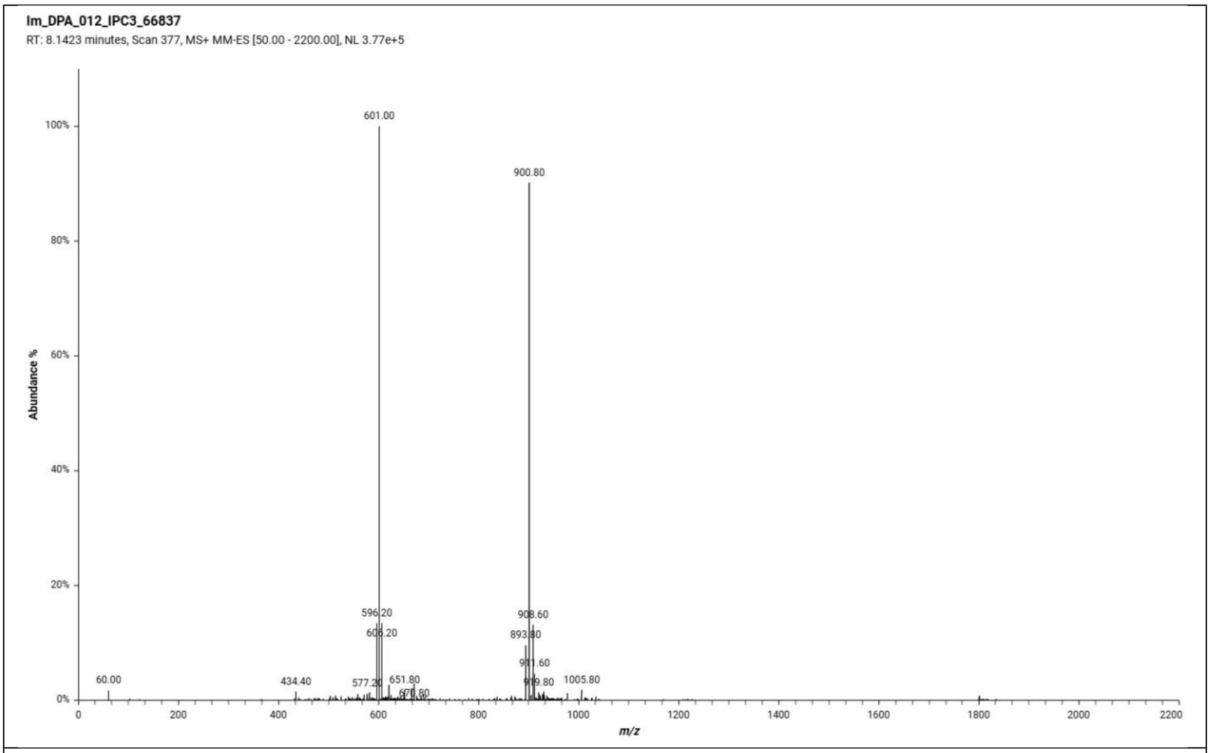
Im_DPA_012_IPC3_66837
RT: 8.1423 minutes, Scan 377, MS+ MM-ES [50.00 - 2200.00], NL 3.77e+5
Abundance %
m/z
601.00
900.80
596.20
908.60
893.80
1005.80
434.40
60.00

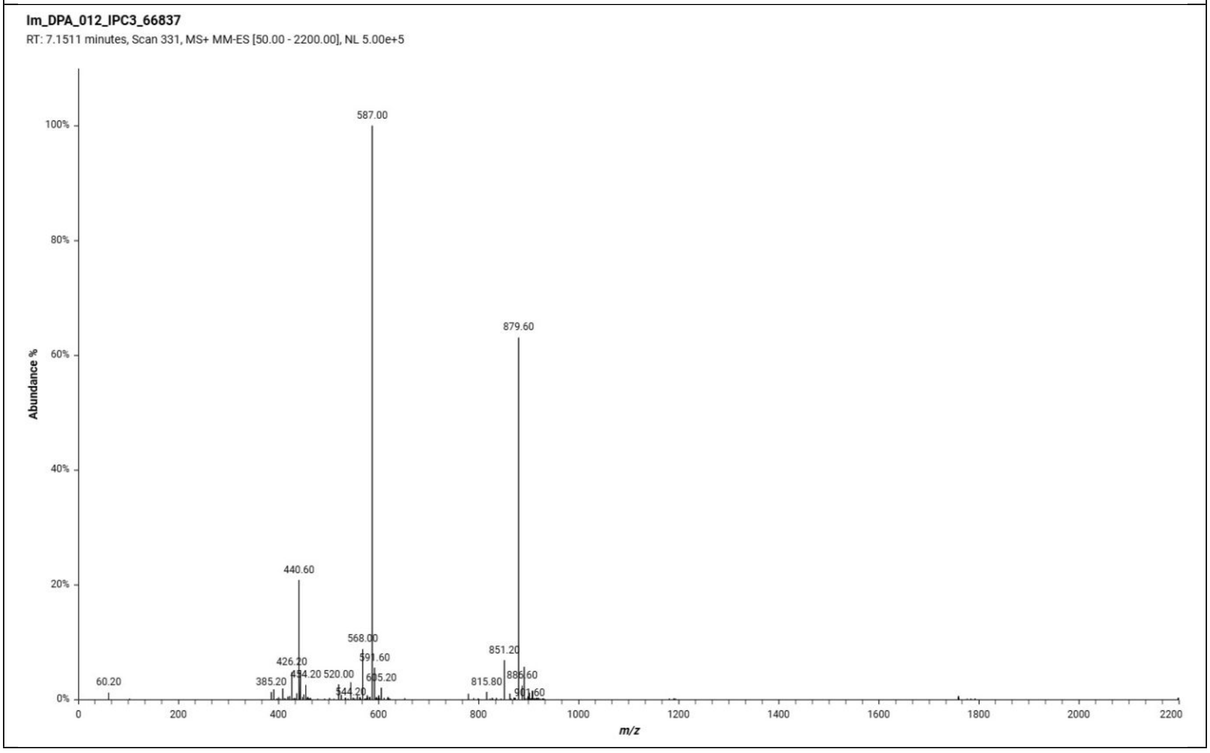
Im_DPA_012_IPC3_66837
RT: 7.1511 minutes, Scan 331, MS+ MM-ES [50.00 - 2200.00], NL 5.00e+5
Abundance %
m/z
587.00
879.60
440.60
568.00
851.20
426.20
520.00
385.20
815.80
60.20

Figure S11: UV traces (at 210nm) and mass spectra of LC-MS analysis of the mini-cleavage product of peptidyl resin **7”**, shown in figure 5b

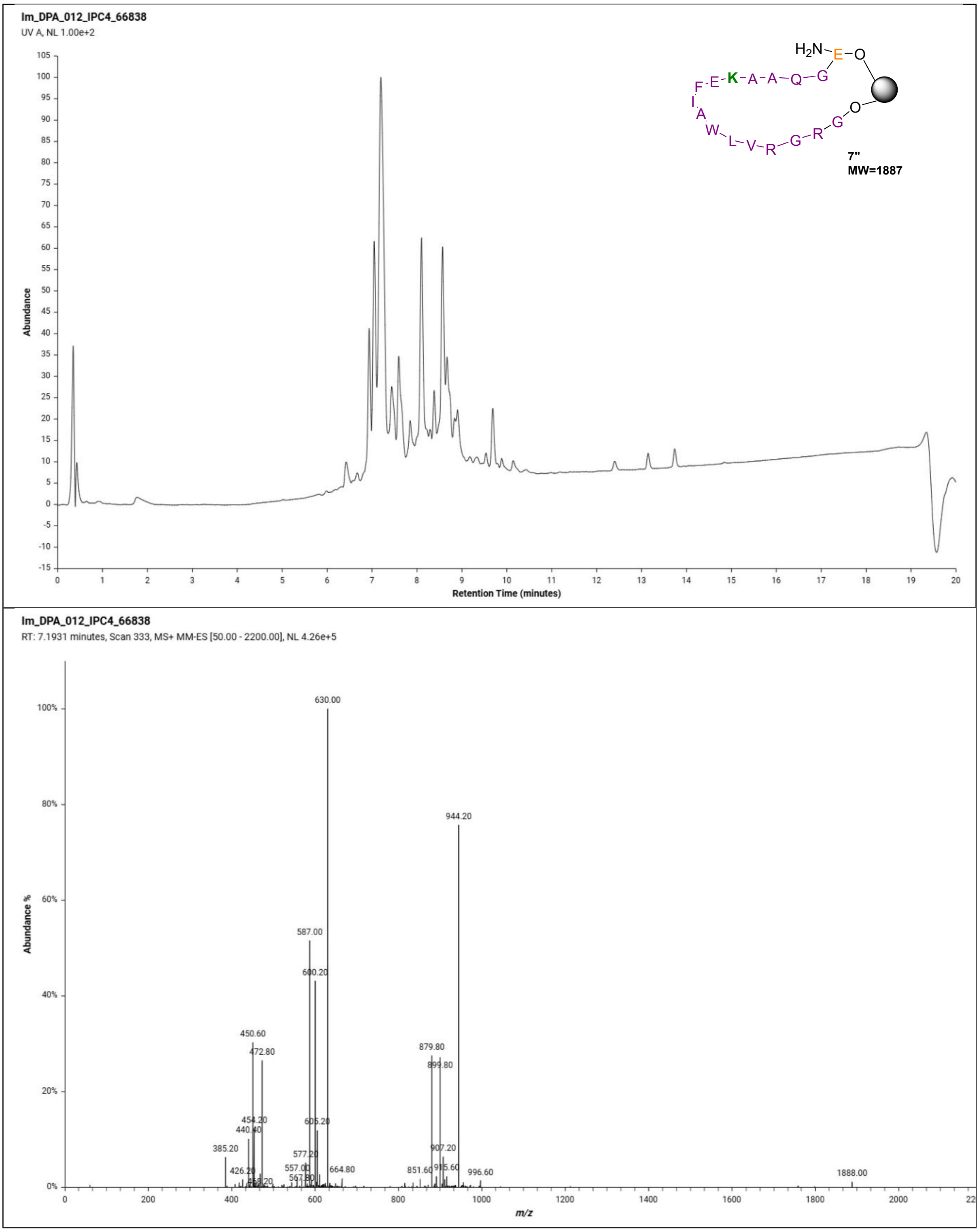

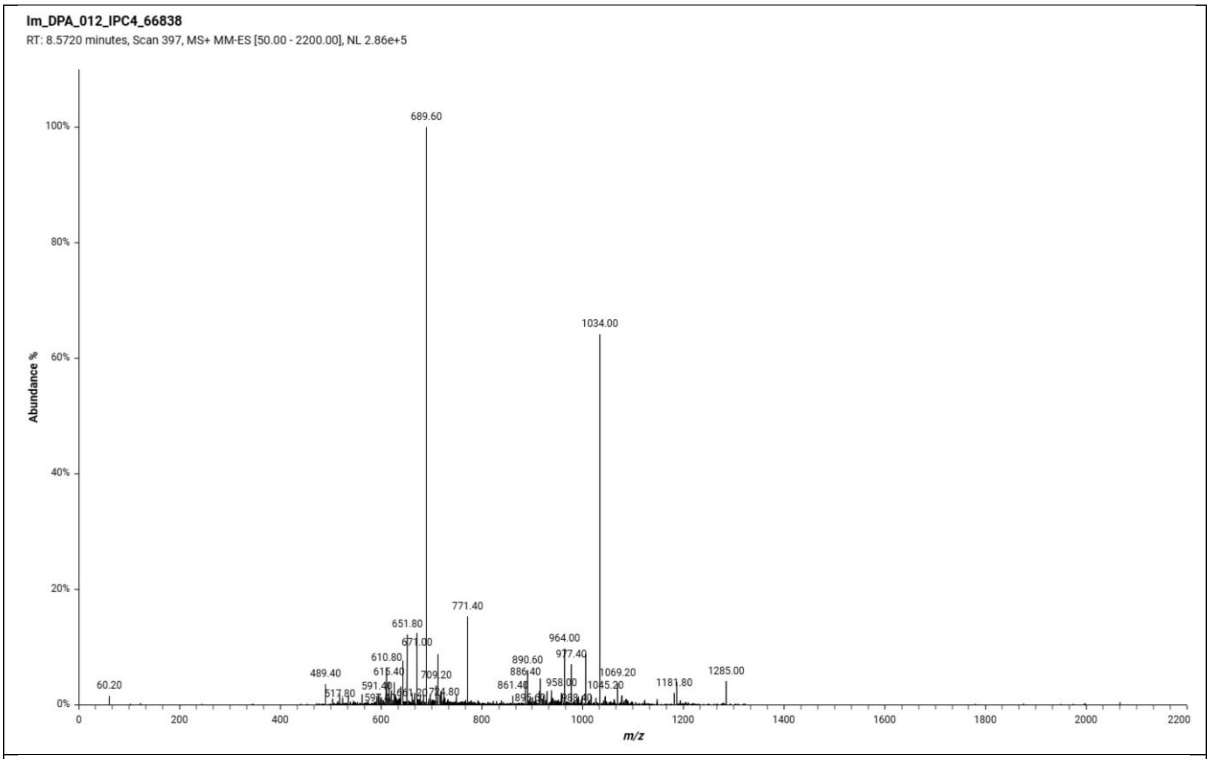

Im_DPA_012_IPC4_66838
RT: 8.5720 minutes, Scan 397, MS+ MM-ES [50.00 - 2200.00], NL 2.86e+5
Abundance %
m/z
689.60
1034.00
771.40
651.80
671.00
610.80
615.40
591.40
709.20
489.40
60.20
964.00
977.40
890.60
886.40
861.40
958.00
1069.20
1045.20
1181.80
1285.00

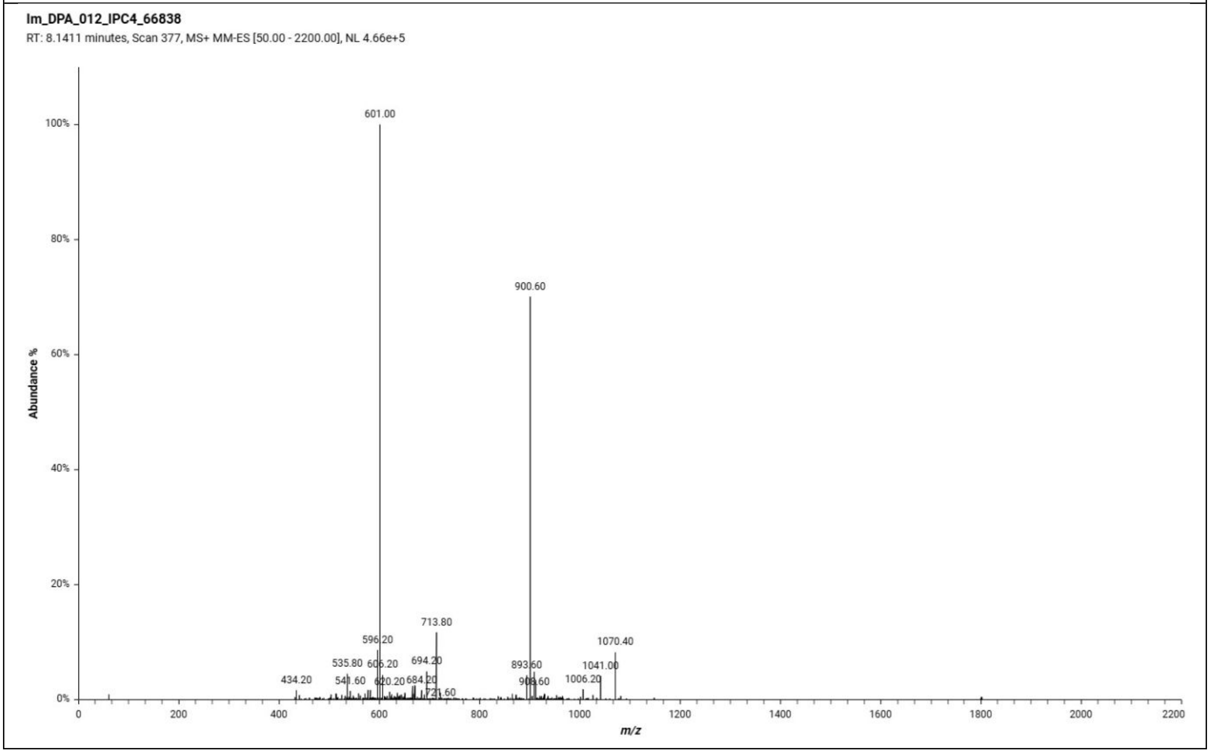

Im_DPA_012_IPC4_66838
RT: 8.1411 minutes, Scan 377, MS+ MM-ES [50.00 - 2200.00], NL 4.66e+5
Abundance %
m/z
601.00
900.60
713.80
596.20
535.80
606.20
694.20
434.20
620.20
684.20
893.60
1070.40
1041.00
1006.20

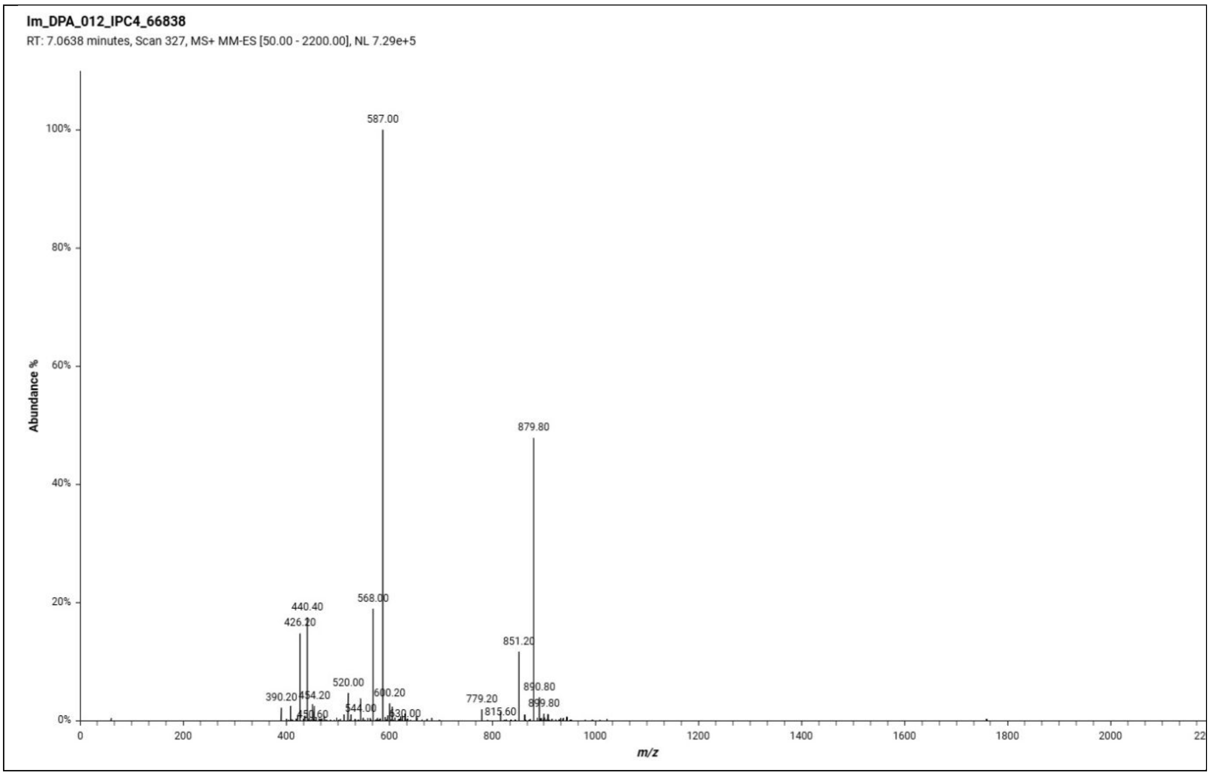
Im_DPA_012_IPC4_66838
RT: 7.0638 minutes, Scan 327, MS+ MM-ES [50.00 - 2200.00], NL 7.29e+5
Abundance %
m/z
587.00
879.80
568.00
440.40
426.20
851.20
520.00
890.80
390.20
454.20
600.20
779.20
899.80
544.00
815.60
630.00

Figure S12: UV chromatograms (at 210, 280 nm) and mass spectra from the LC–MS analysis of the mini-cleavage product of the peptidyl resin obtained after the lipidation step (step 10), as reported in Table 4, entry 1.

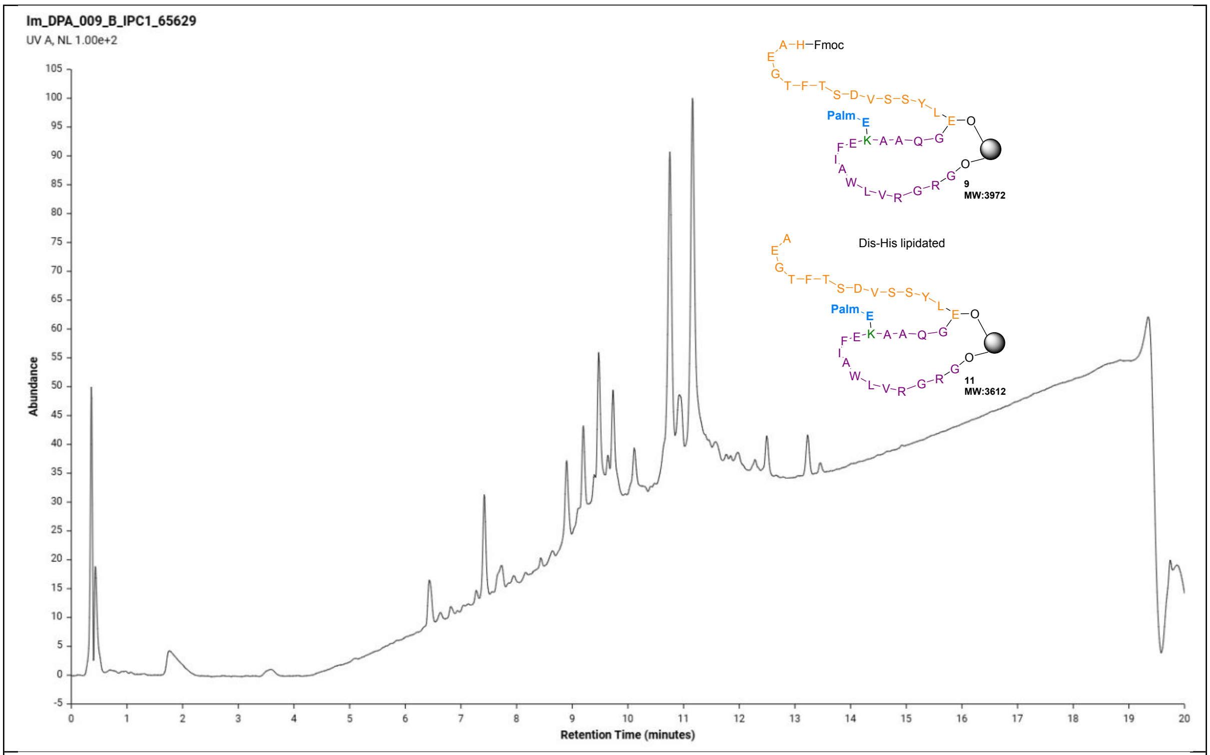

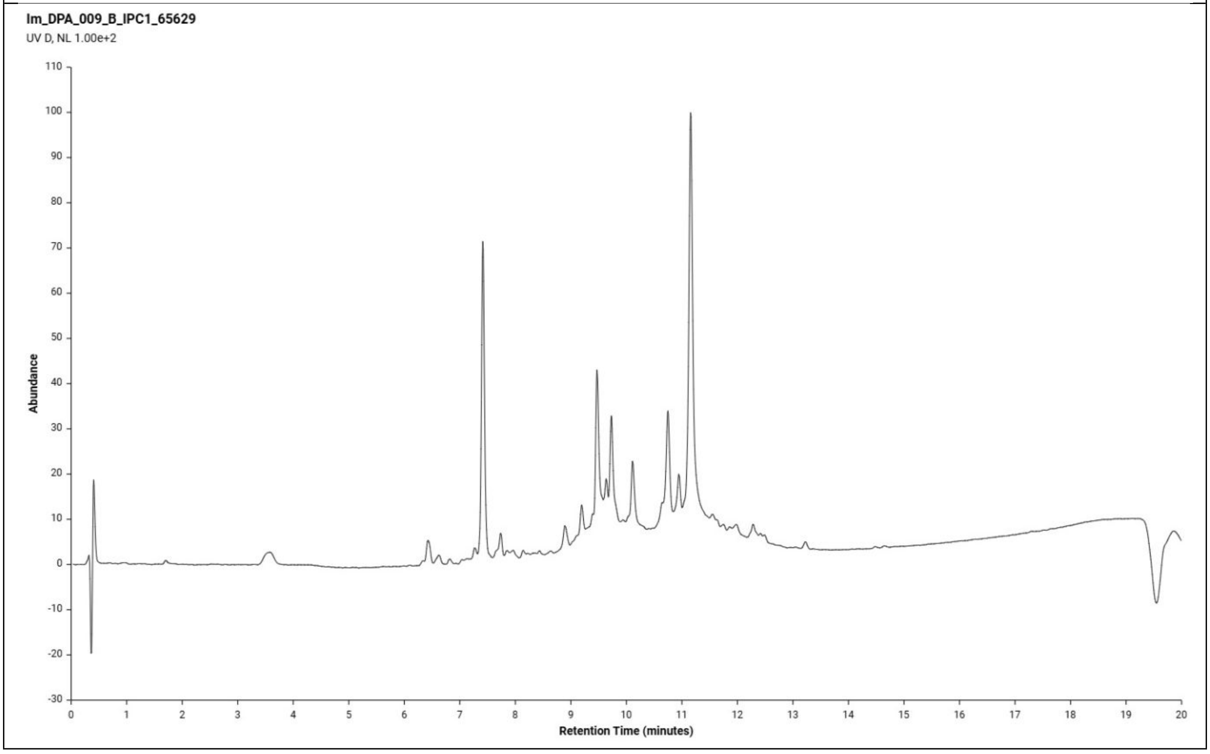

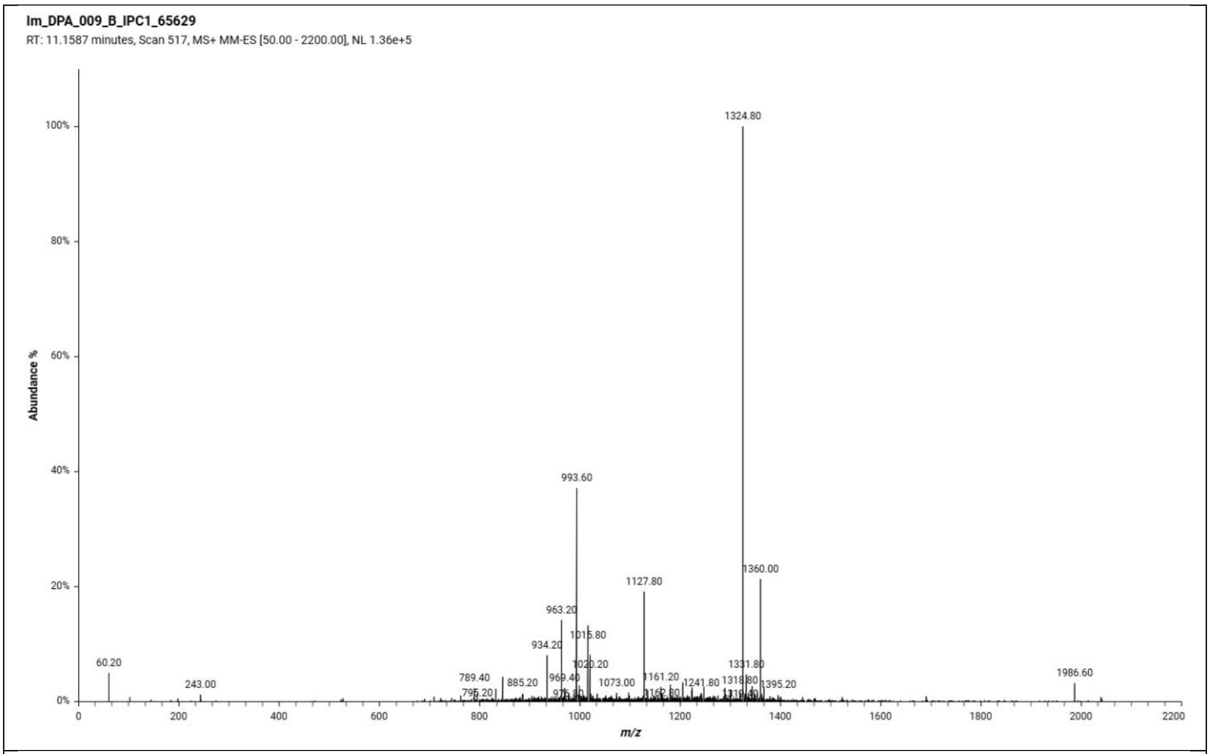

Im_DPA_009_B_IPC1_65629
RT: 11.1587 minutes, Scan 517, MS+ MM-ES [50.00 - 2200.00], NL 1.36e+5
Abundance %
m/z
1324.80
993.60
1360.00
1127.80
963.20
1015.80
934.20
1020.20
60.20
1331.80
1986.60
789.40
885.20
969.40
1073.00
1161.20
1241.80
1318.80
1395.20
243.00
795.20

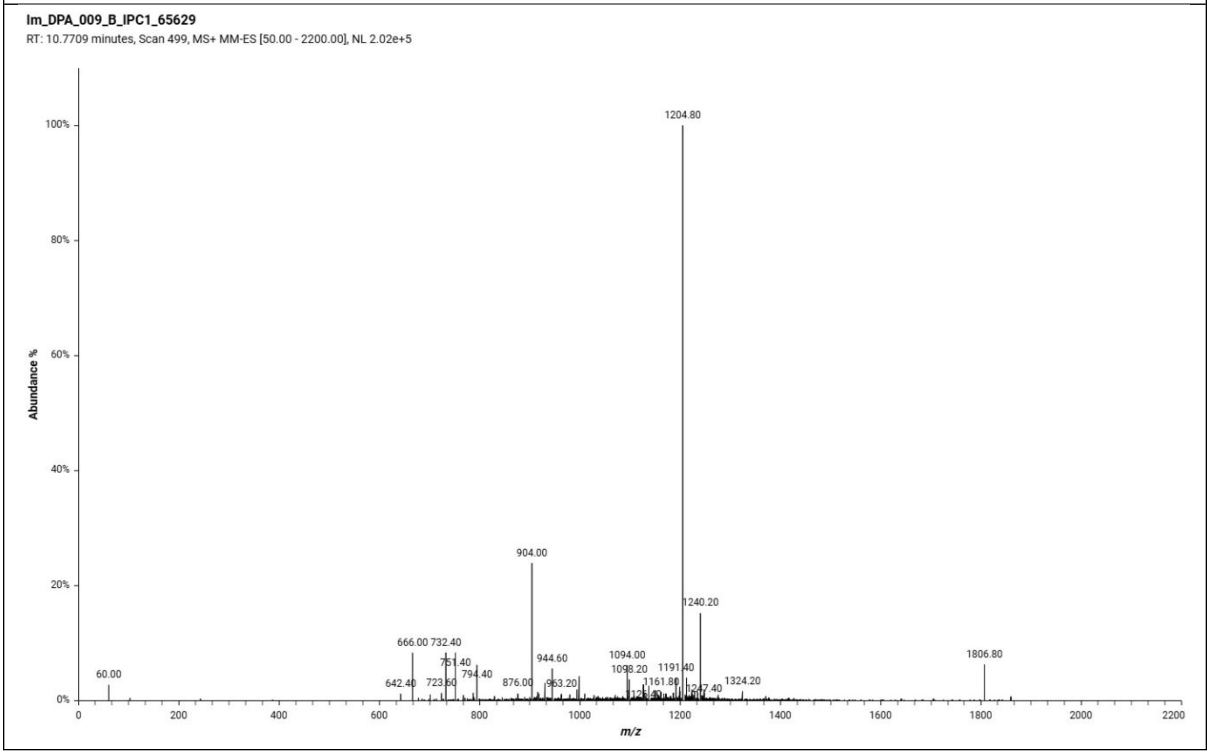

Im_DPA_009_B_IPC1_65629
RT: 10.7709 minutes, Scan 499, MS+ MM-ES [50.00 - 2200.00], NL 2.02e+5
Abundance %
m/z
1204.80
904.00
1240.20
666.00
732.40
1094.00
1806.80
751.40
944.60
1098.20
1191.40
794.40
60.00
642.40
723.60
876.00
963.20
1161.80
1247.40
1324.20

Figure S13: UV chromatograms (at 210, 280 nm) and mass spectra from the LC–MS analysis of the mini-cleavage product of the peptidyl resin obtained after the lipidation step (step 10), as reported in Table 4, entry 2 (third coupling).

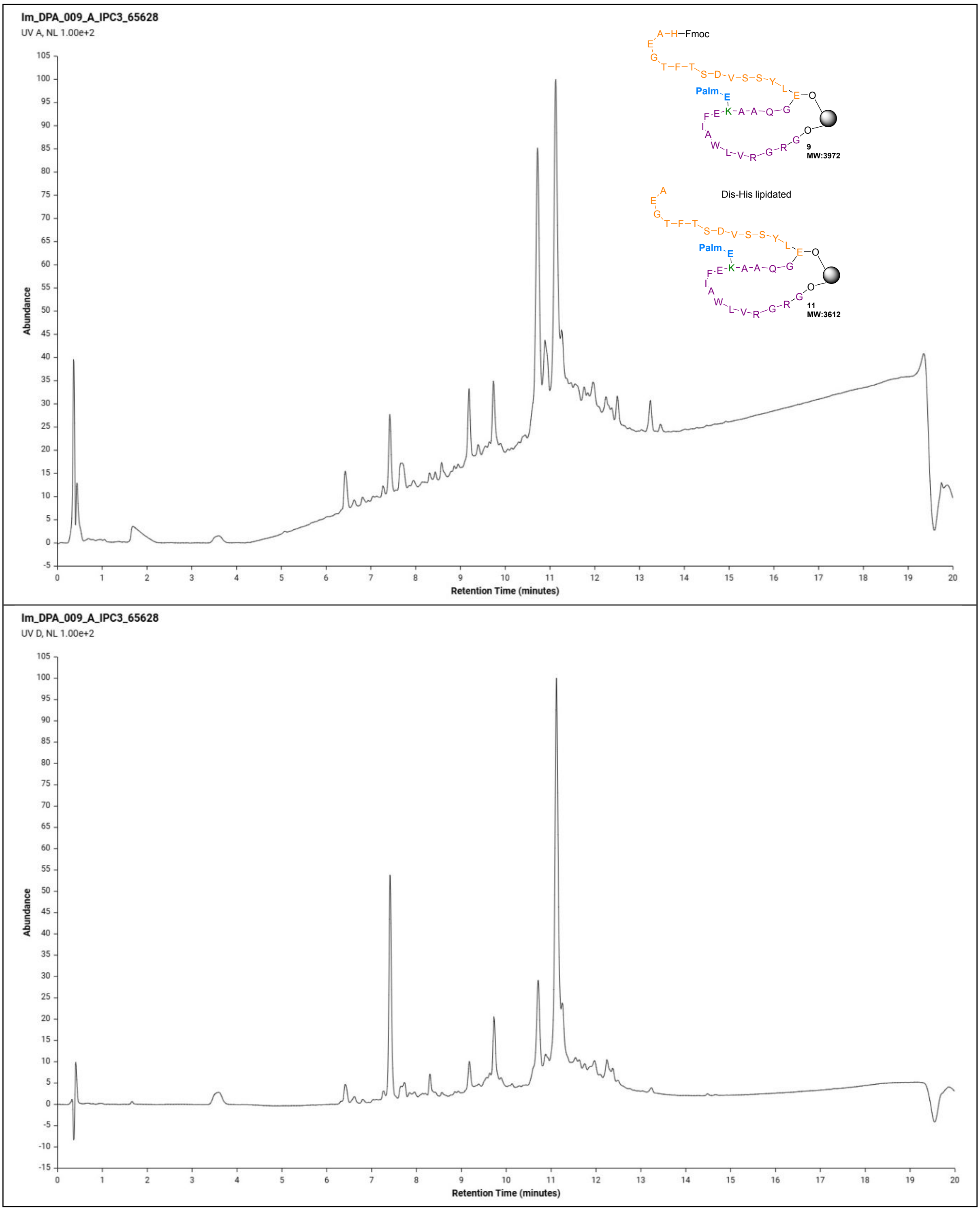

Im_DPA_009_A_IPC3_65628
RT: 11.1589 minutes, Scan 517, MS+ MM-ES [50.00 - 2200.00], NL 2.46e+5

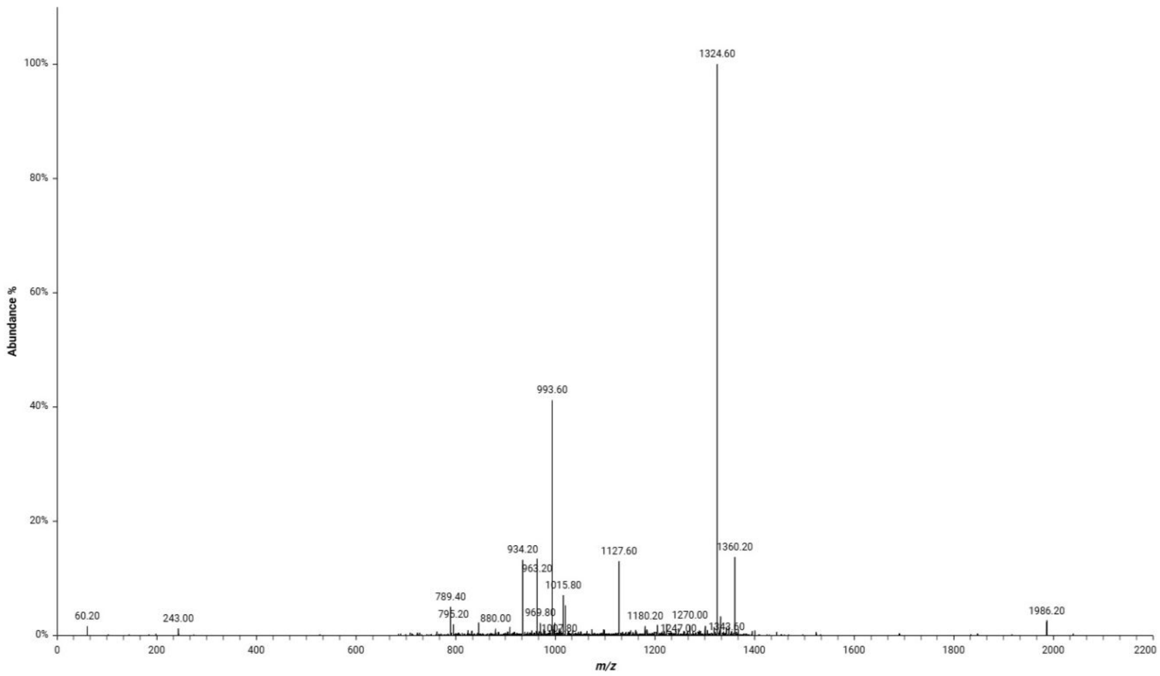

Im_DPA_009_A_IPC3_65628
RT: 10.7280 minutes, Scan 497, MS+ MM-ES [50.00 - 2200.00], NL 2.29e+5

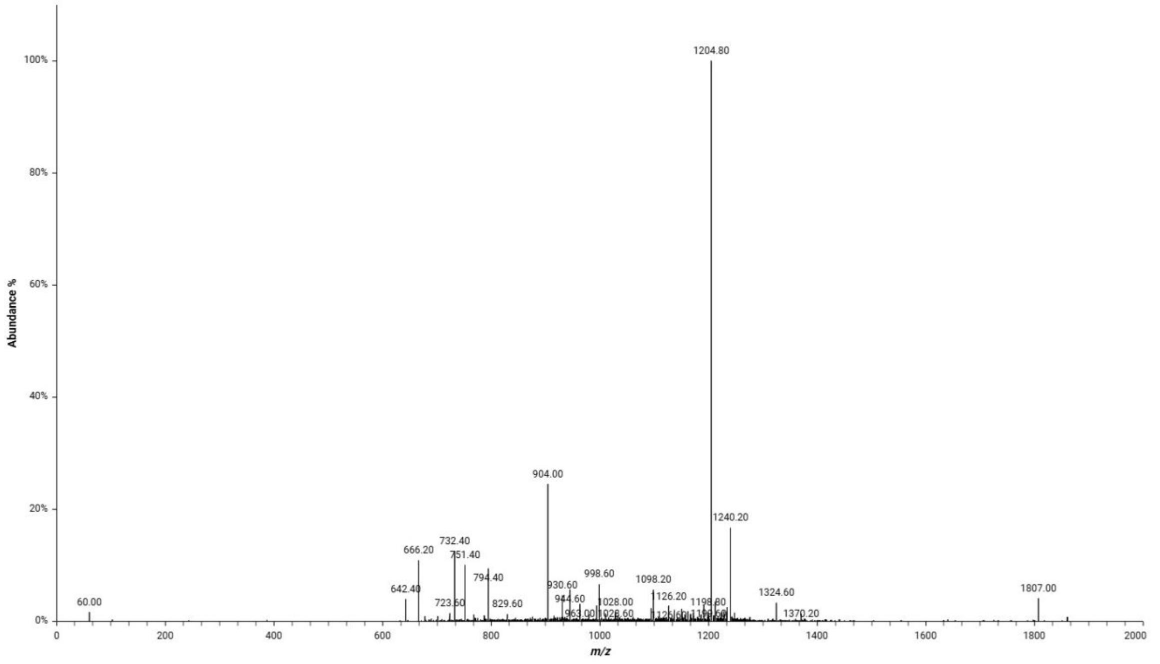

Figure S14: UV chromatograms (at 210, 280 nm) and mass spectra from the LC–MS analysis of the mini-cleavage product of the peptidyl resin obtained after the lipidation step (step 10), as reported in Table 4, entry 3

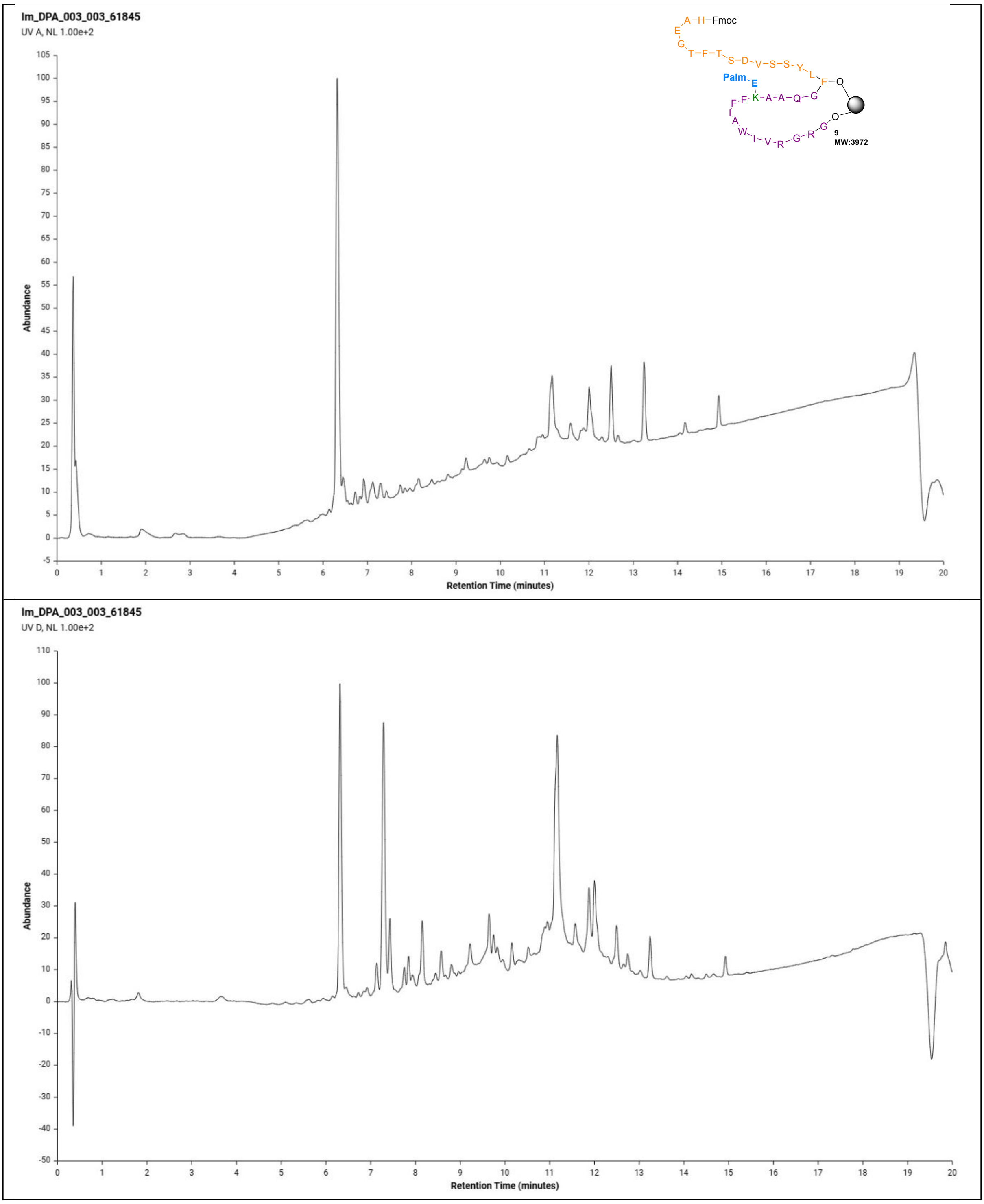

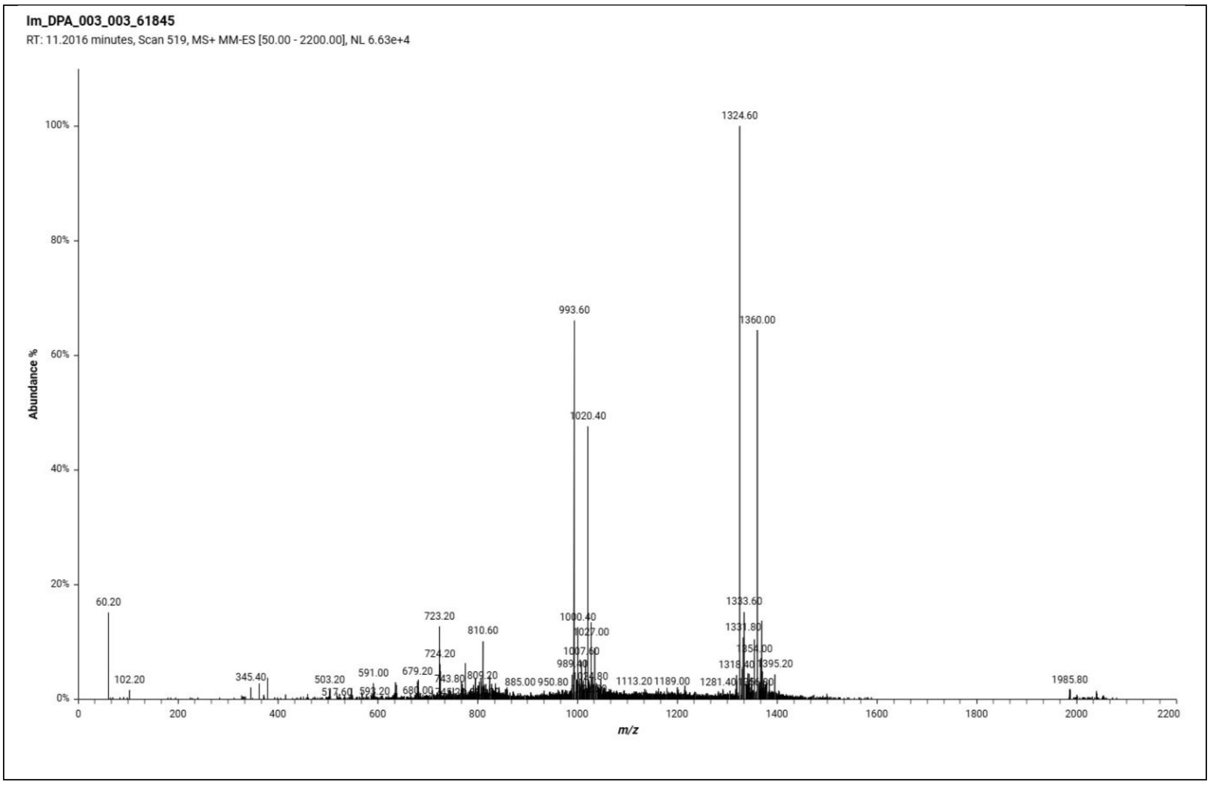
Im_DPA_003_003_61845
RT: 11.2016 minutes, Scan 519, MS+ MM-ES [50.00 - 2200.00], NL 6.63e+4
Abundance %
m/z
1324.60
993.60
1360.00
1020.40
60.20
102.20
345.40
503.20
591.00
679.20
723.20
724.20
810.60
885.00
950.80
1113.20
1189.00
1281.40
1333.60
1395.20
1985.80

Figure S15: UV chromatograms (at 210, 280 nm) and mass spectra from the LC–MS analysis of the mini-cleavage product of the peptidyl resin obtained after the lipidation step (step 10), as reported in Table 4, entry 4 (third coupling).

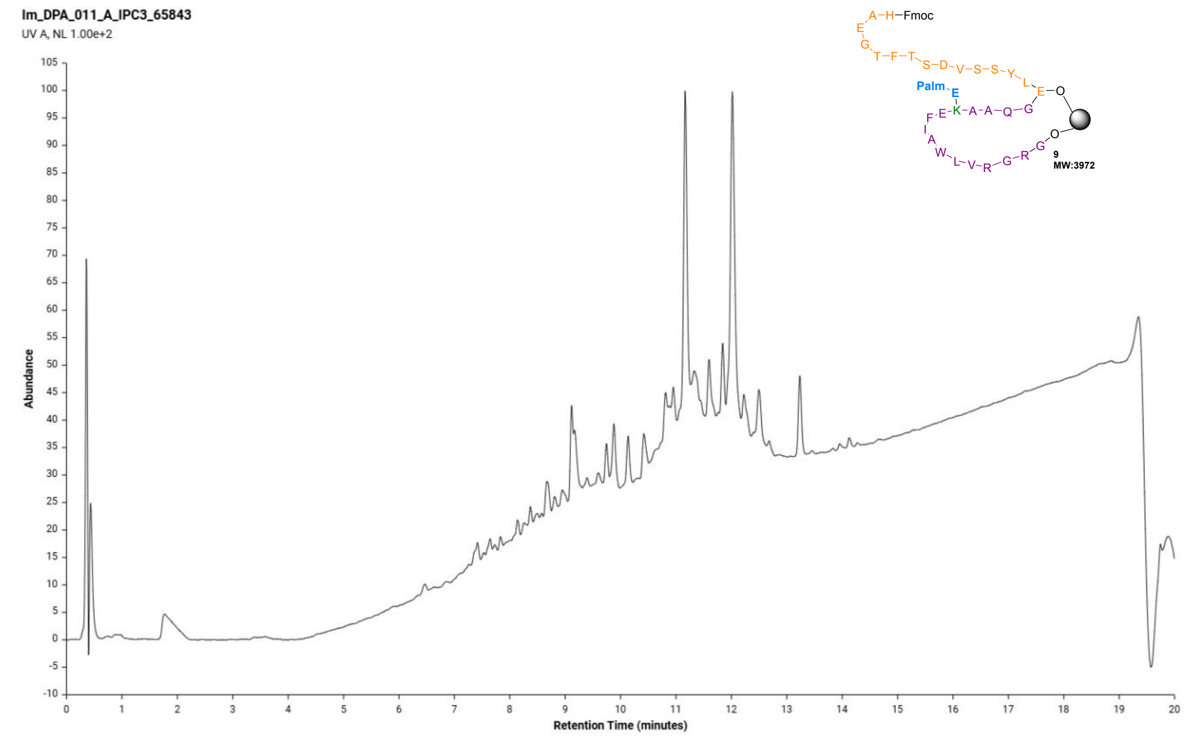

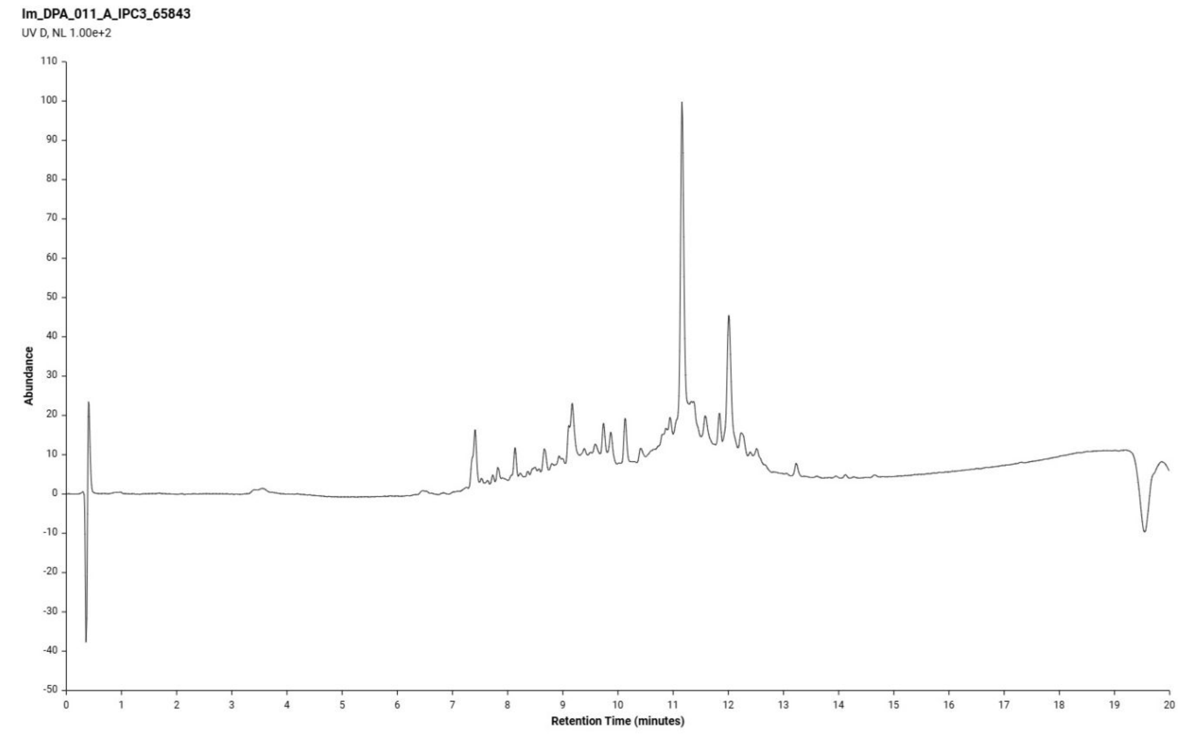

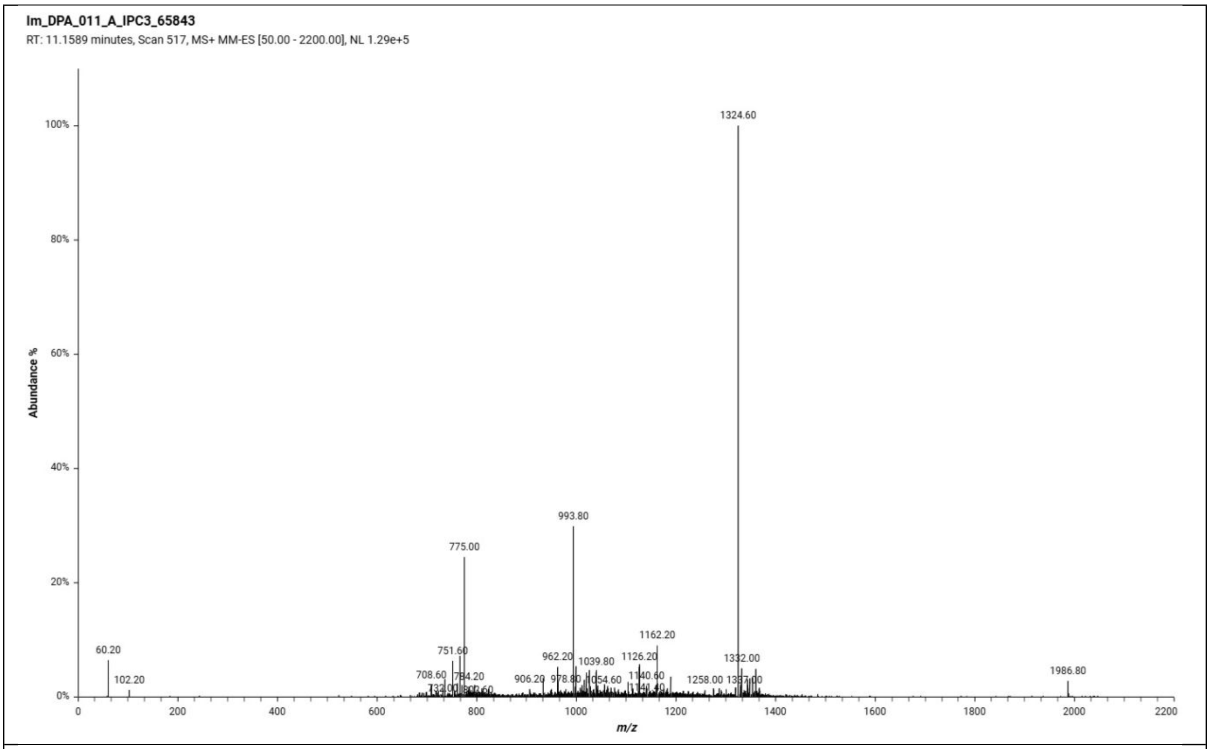
Im_DPA_011_A_IPC3_65843
RT: 11.1589 minutes, Scan 517, MS+ MM-ES [50.00 - 2200.00], NL 1.29e+5
Abundance %
m/z
1324.60
993.80
775.00
1162.20
60.20
751.60
962.20
1039.80
1126.20
1332.00
1986.80
708.60
102.20
906.20
978.80
1140.60
1258.00

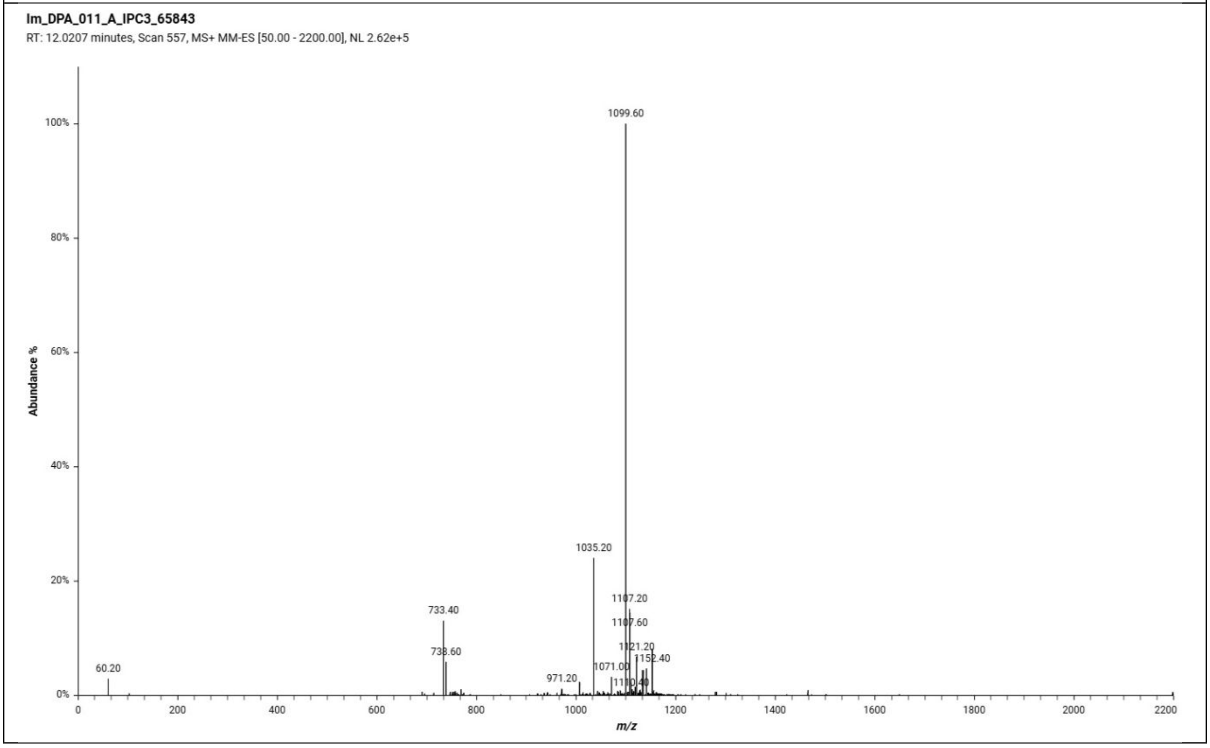
Im_DPA_011_A_IPC3_65843
RT: 12.0207 minutes, Scan 557, MS+ MM-ES [50.00 - 2200.00], NL 2.62e+5
Abundance %
m/z
1099.60
1035.20
733.40
738.60
60.20
971.20
1071.00
1152.40

Figure S16: UV chromatograms (at 210, 280 nm) and mass spectra from the LC–MS analysis of the mini-cleavage product of the peptidyl resin obtained after the lipidation step (step 10), as reported in Table 4, entry 5 (third coupling).

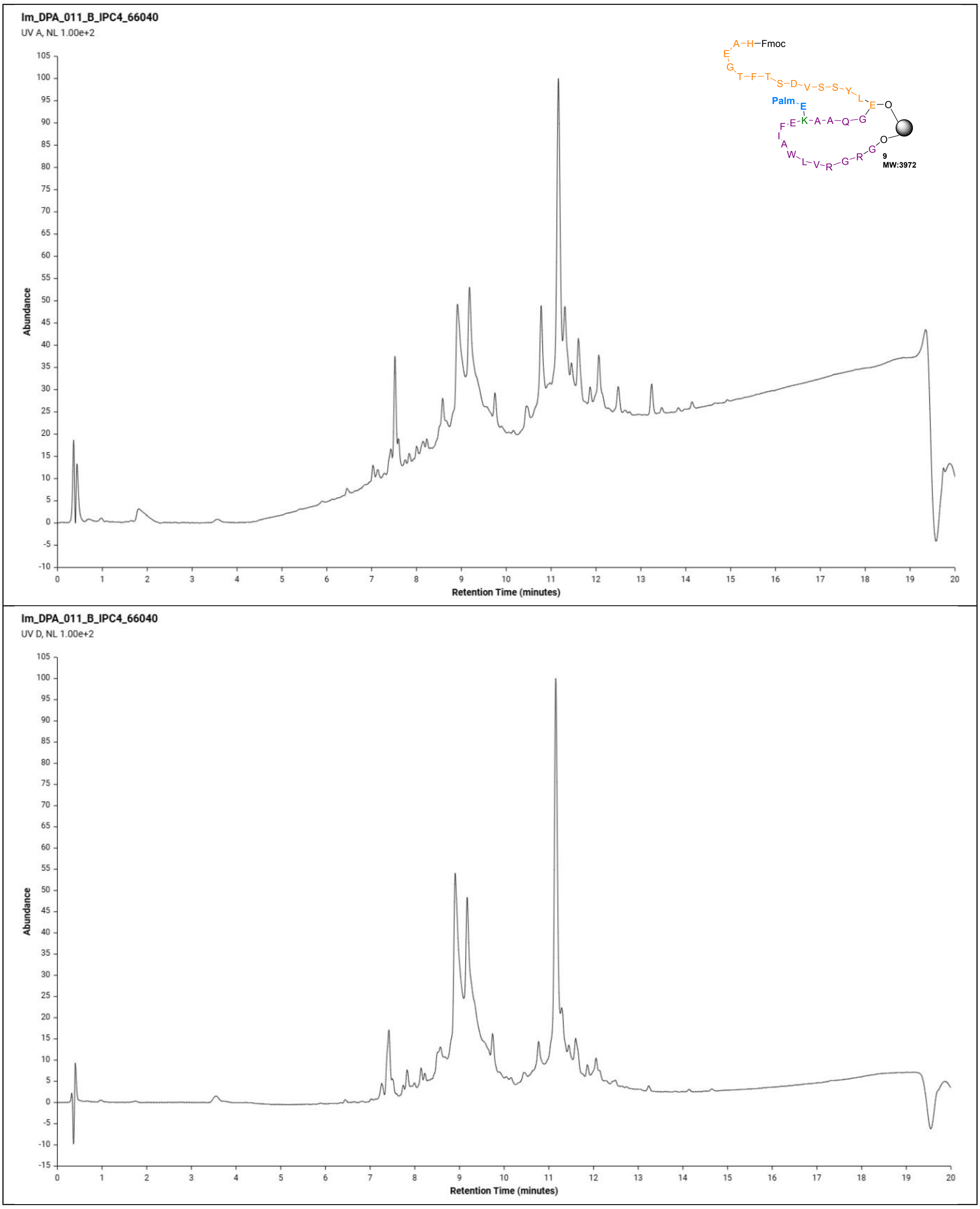

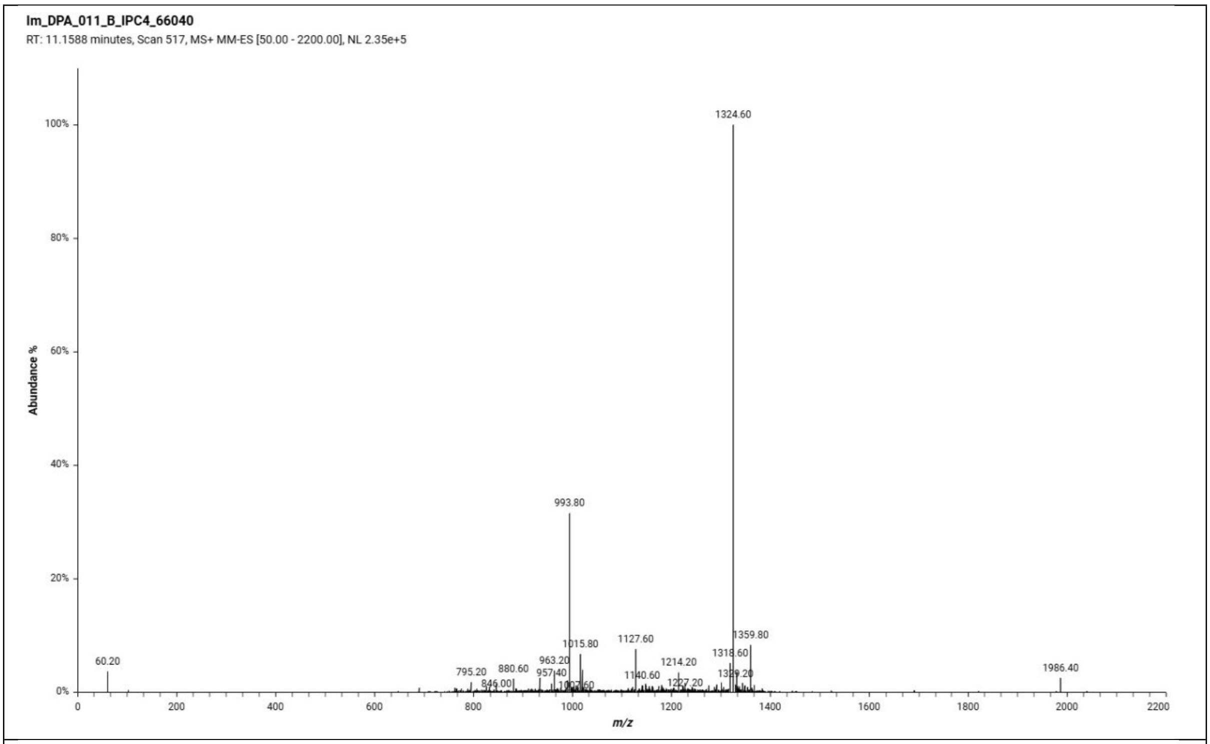
Im_DPA_011_B_IPC4_66040
RT: 11.1588 minutes, Scan 517, MS+ MM-ES [50.00 - 2200.00], NL 2.35e+5
Abundance %
m/z
1324.60
993.80
60.20
1015.80
1127.60
1359.80
1318.60
1214.20
1986.40
795.20
880.60
963.20

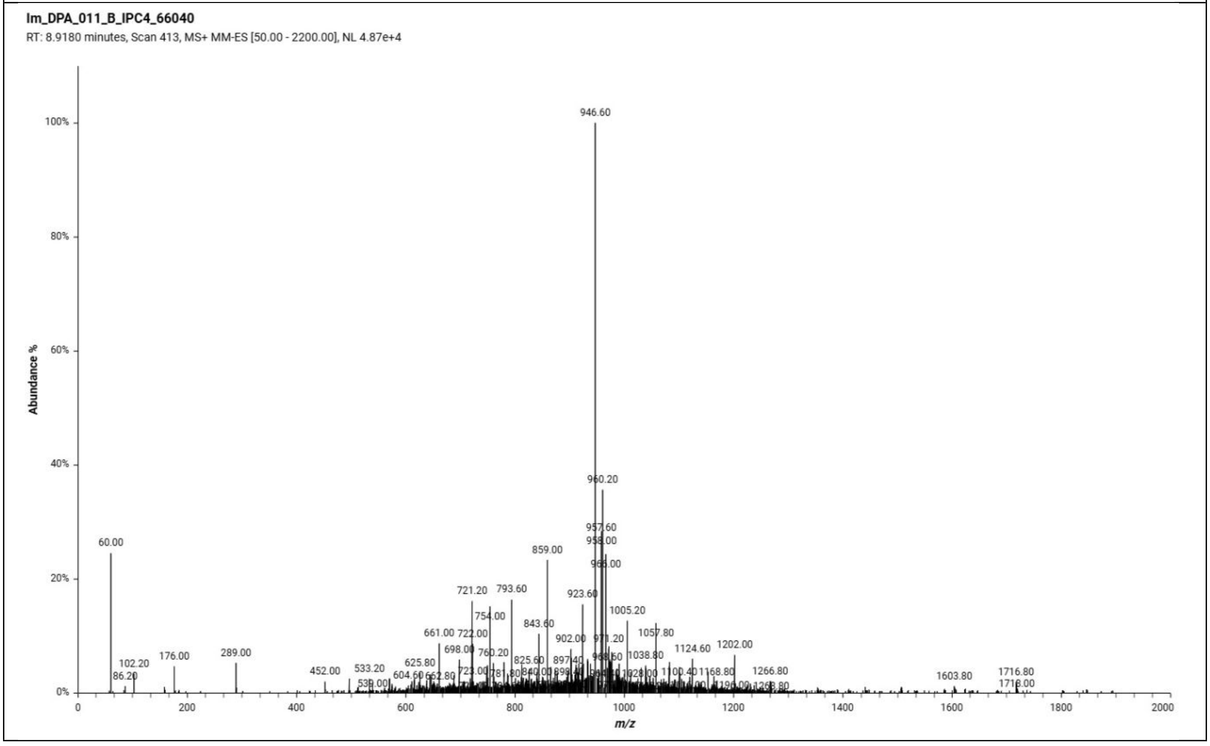
Im_DPA_011_B_IPC4_66040
RT: 8.9180 minutes, Scan 413, MS+ MM-ES [50.00 - 2200.00], NL 4.87e+4
Abundance %
m/z
946.60
960.20
957.60
859.00
60.00
721.20
793.60
754.00
923.60
1005.20
843.60
1057.80
1202.00
1124.60
176.00
289.00
102.20
452.00
533.20
1266.80
1603.80
1716.80

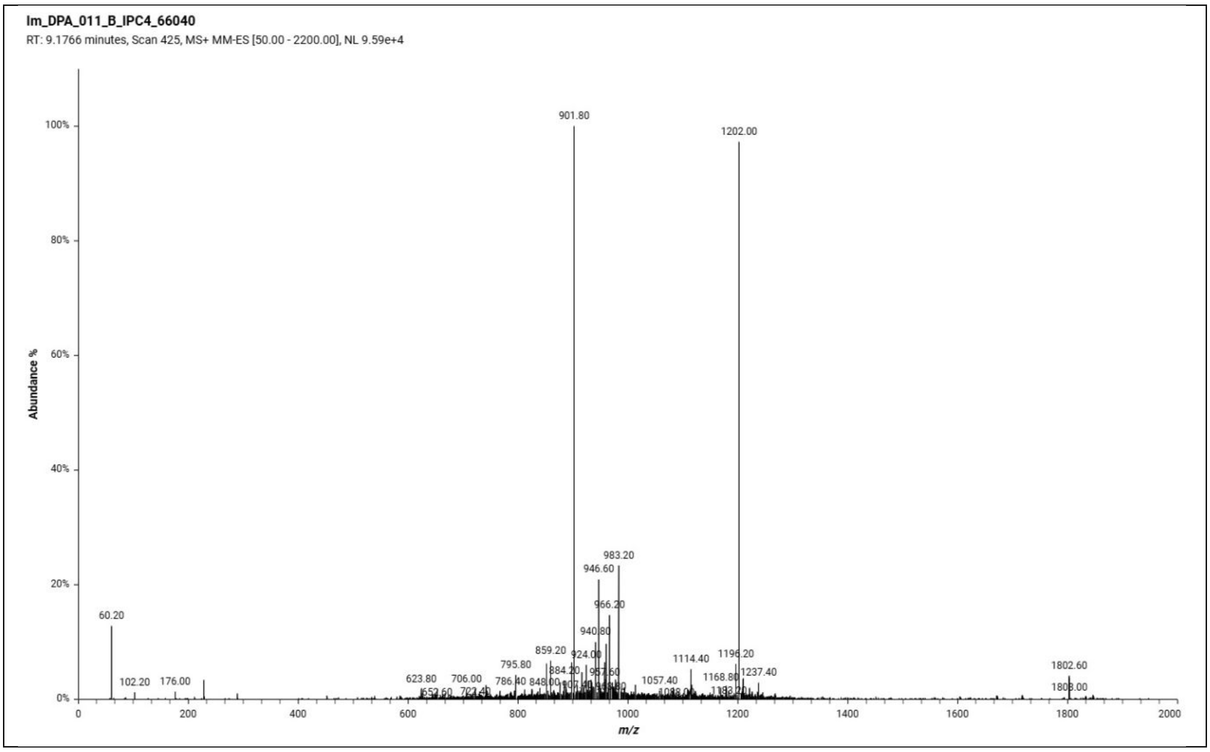
Im_DPA_011_B_IPC4_66040
RT: 9.1766 minutes, Scan 425, MS+ MM-ES [50.00 - 2200.00], NL 9.59e+4
Abundance %
m/z
901.80
1202.00
983.20
946.60
966.20
940.80
60.20
859.20
924.00
1114.40
1196.20
795.80
1802.60
1237.40
102.20
176.00
623.80
706.00
786.40
1057.40
1168.80

Figure S17: UV chromatograms (at 210) and mass spectra from the LC–MS analysis of pNZ-Glu(OAll)-OH

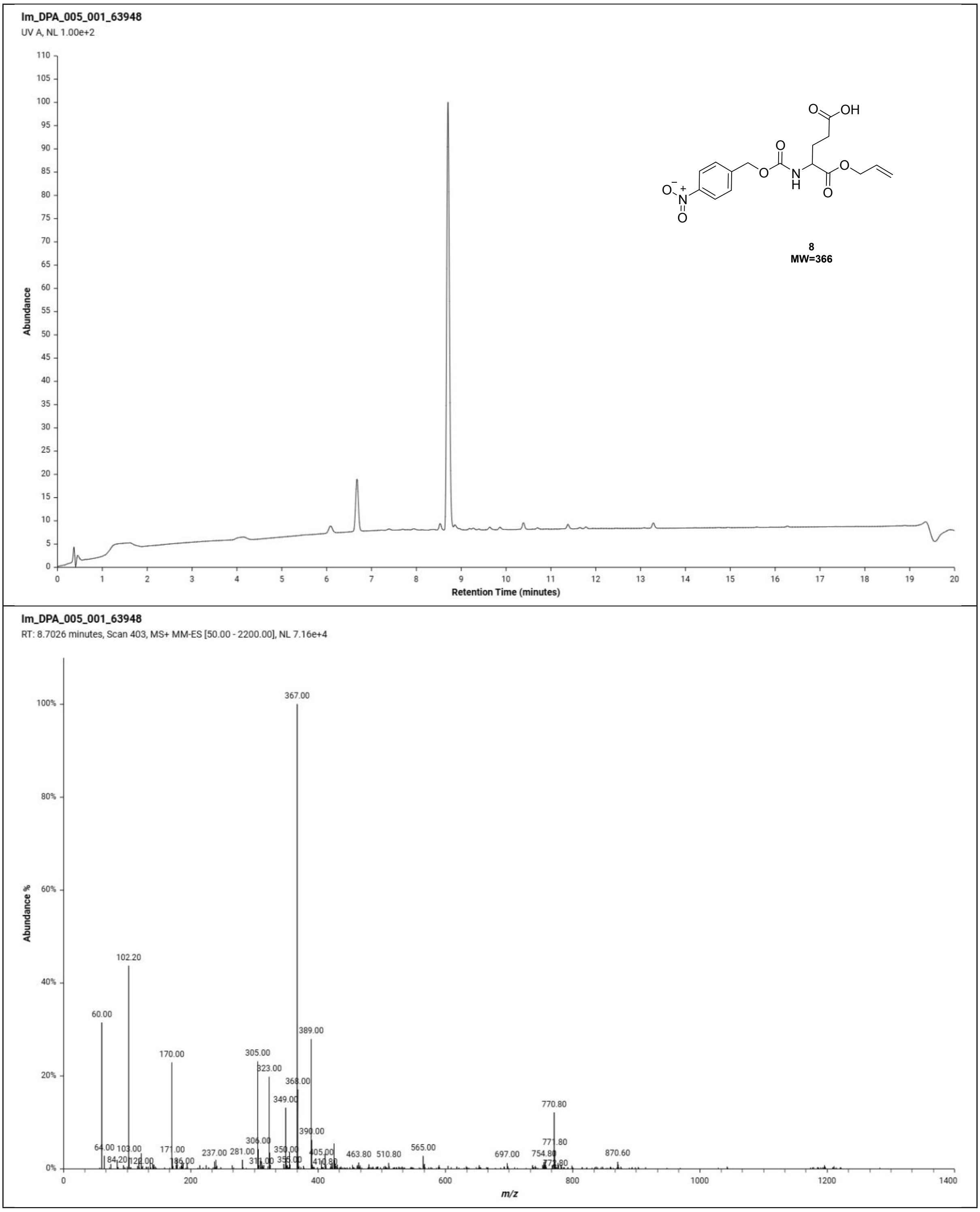

Figure S18: ^{1}H-NMR, ^{13}C-NMR, COSEY, HSQC NMR analysis of pNZ-Glu(OAll)-OH

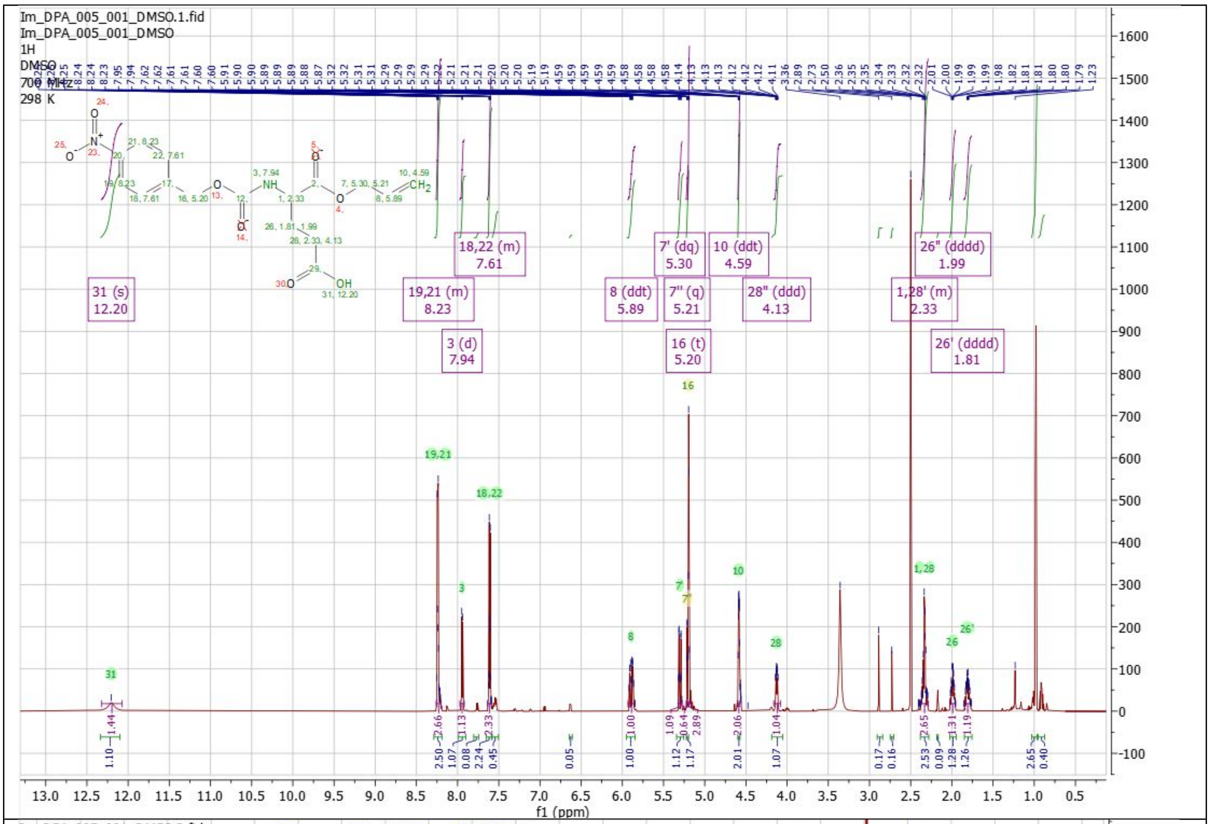

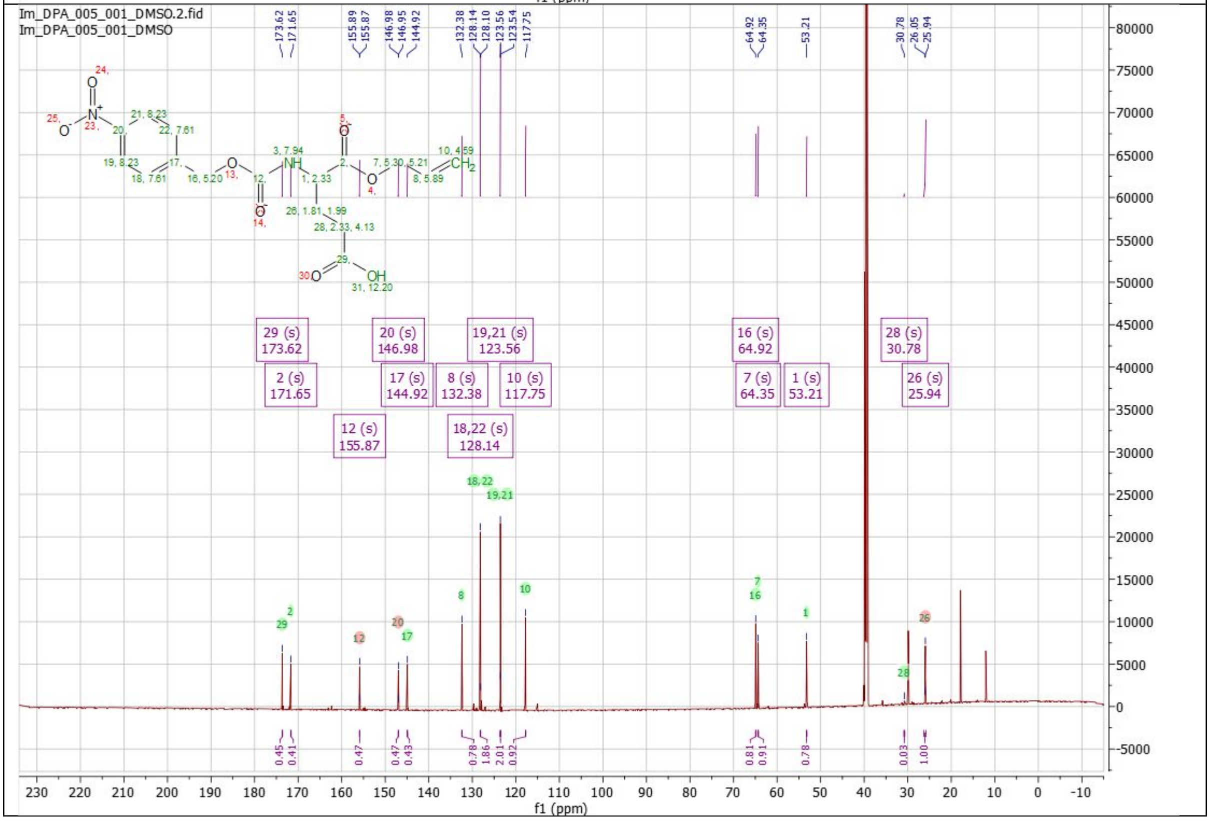

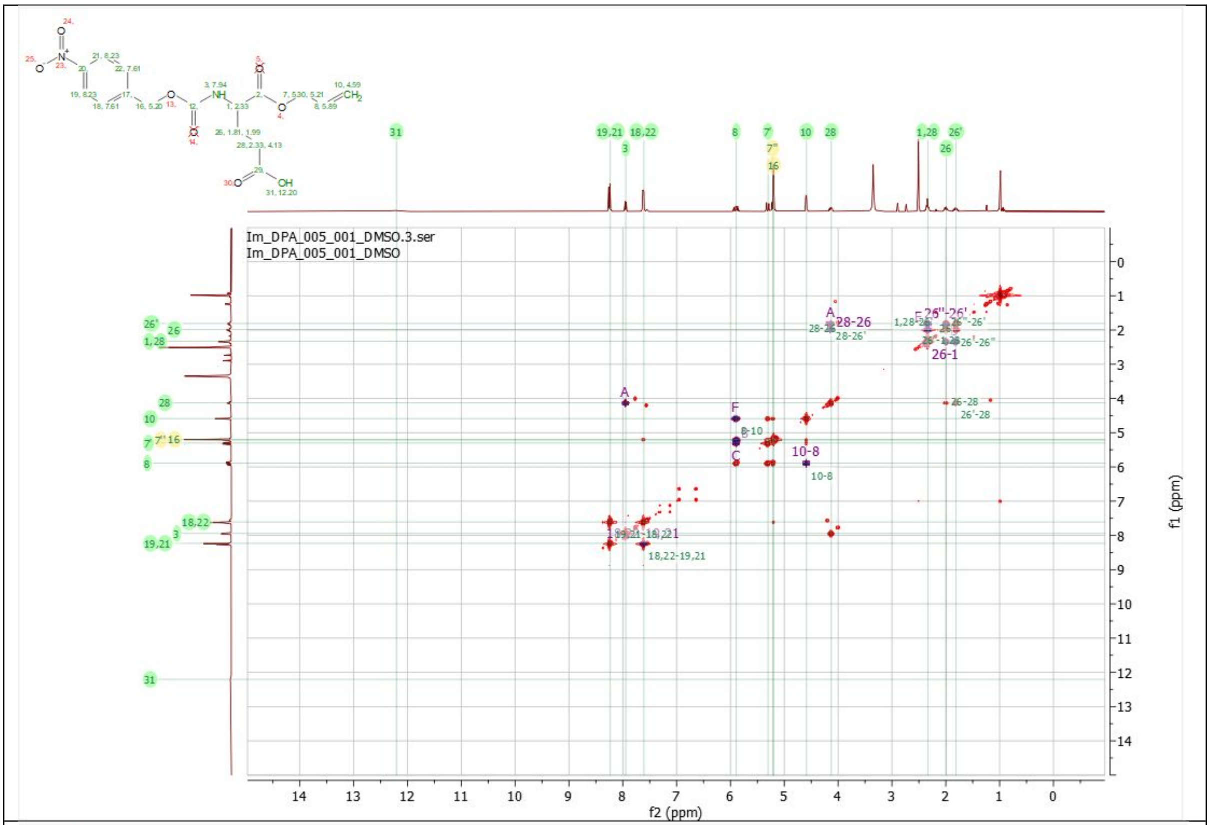
Im_DPA_005_001_DMSO.3.ser
Im_DPA_005_001_DMSO
f2 (ppm)
f1 (ppm)

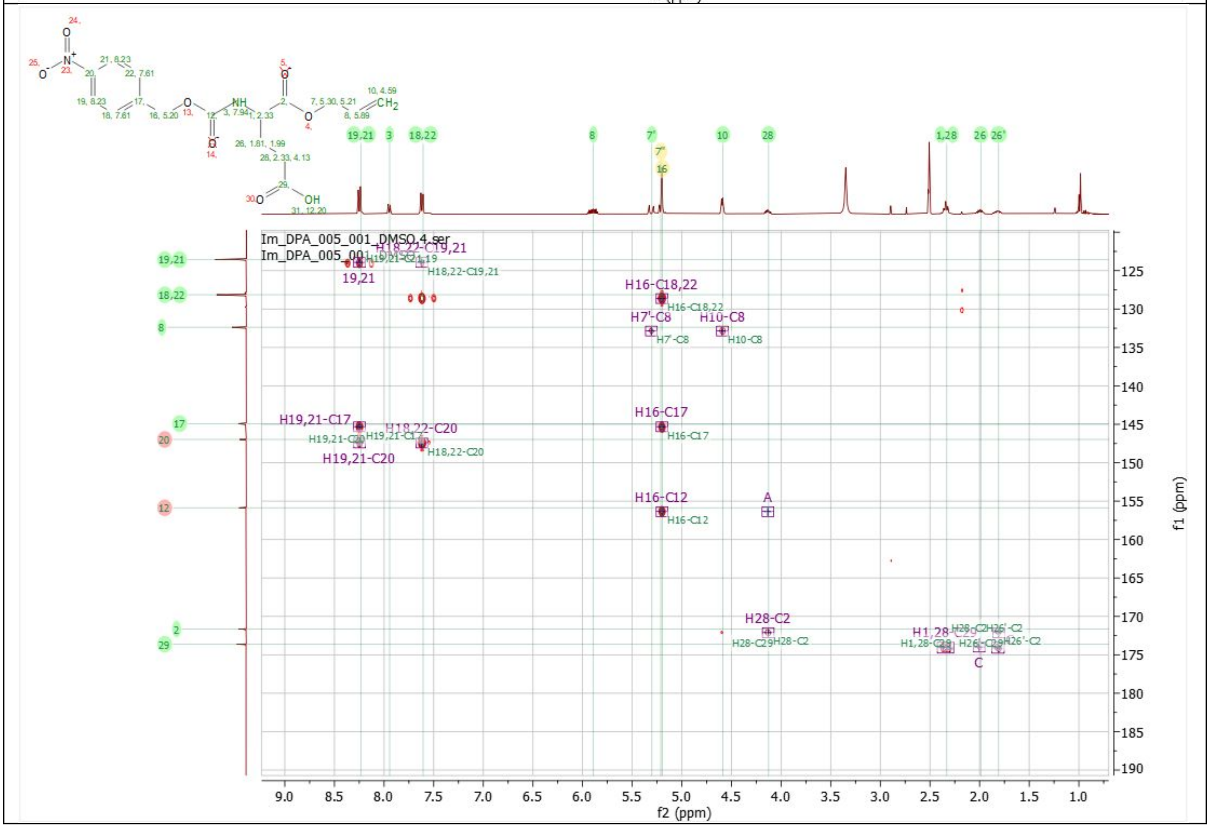
Im_DPA_005_001_DMSO.4.ser
f2 (ppm)
f1 (ppm)

Table S1. Alloc/Allyl removal trials in original and modified RSE: automation vs. manual (% conversion shown).

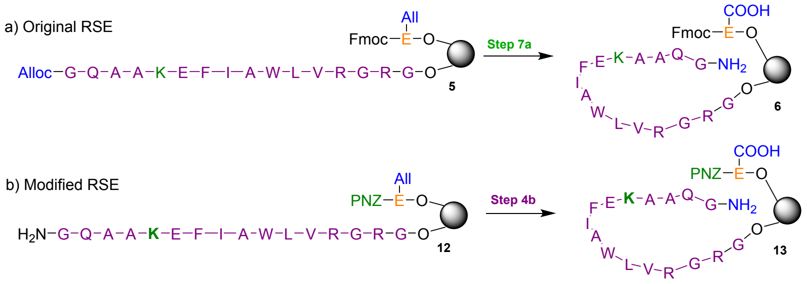

Trial	Route		Conditions<br>**[Pd(PPh$_3$)$_4$]:PhSiH$_3$**	Conversion to desired product
1	Original RSE	Manual<br>Step 7a	0.1eq : 10 eq in DCM<br>RT, 2x30 min.	100%
2	Modified RSE	Synthesizer<br>Step 4a	0.1eq : 10 eq in dry DMF<br>RT, 2x30 min.	NA but following steps suggests >70% removal
3	Original RSE	synthesizer	0.05eq : 5 eq/Alloc DMF<br>**50 °C**, 2x30 min.<br>Then<br>0.25eq : 15 eq/Alloc dry DMF<br>**40 °C**, 2x5 min.	(**24%** removal)<br><br>Then (from **24% to 31%** removal (~7%))
4			0.1eq : 10 eq<br>**RT**, 2x30 min.+1x15min. dry DMF	NA FAILED

Table S2. pNZ removal trials in original and modified RSE: automation vs. manual (% conversion shown).

Trial	Route		Conditions	Conversion to desired product
1	Original RSE	Manual	**SnCl$_2$: HCl (step 6b)**	2M: 1.6 mM<br>RT, 2x30 min.<br>(N.A.)<br>Assumed complete considering the loading experiment and the absence of excess chains
2	Modified RSE	synthesizer	2M: 1.6 mM<br>RT, 2x30 min.	(60% removal)<br>It was supposed to use 6M
3			**3**M: 1.6 mM<br>**RT**, **3**x30 min.	NA due to failed previous Alloc removal step

Table S3. On-resin cyclization (Step 5b) under RSE conditions showing reaction parameters and conversion to the cyclized product.

Trial	Route		Scale (mmol)	Conditions **DIC:Oxyma** (step 5b)	Conversion to desired product
1	RSE	Manual	0.1	5eq : 5 eq<br>RT, 9 hrs	(~90% conversion)
2	Modified RSE	Synthesizer	0.1	5eq : 5 eq<br>50 °C, 60 min.	(~70% conversion)
3	Modified RSE	Synthesizer	0.1	5eq : 5 eq<br>50 °C, 75 min.	NA due to failed previous Alloc removal step
4	RSE	Hybrid	0.1	5eq : 5 eq<br>50 °C, 90 min	~40% cyclised product, with 29% excess unreacted chain 1 was observed
5	RSE	Hybrid	1.0	5eq : 5 eq<br>50 °C, 90 min	36% cyclised product, many method and scale-related by-products observed, with 21% excess unreacted chain 1
